# Exploring the Dermocosmetic Value of Synthetic Aminopyrimidine-Thioethers

**DOI:** 10.3390/antiox15070841

**Published:** 2026-07-03

**Authors:** Inês C. C. Costa, Joana Silva, Isabel Oliveira Abreu, Juliana Antunes Gaspar, Susete Pinteus, Celso Alves, Maria L. S. Cristiano, Rui Pedrosa

**Affiliations:** 1 Centro de Ciências do Mar do Algarve, CCMAR, Campus de Gambelas, University of Algarve, 8005-139 Faro, Portugal; a52917@ualg.pt; 2 Department of Chemistry and Pharmacy, Faculty of Sciences and Technology, Campus de Gambelas, University of Algarve, 8005-139 Faro, Portugal; 3 MARE—Marine and Environmental Sciences Center/ARNET—Aquatic Research, ESTM, Polytechnic University of Leiria, 2520-630 Peniche, Portugal; joana.m.silva@ipleiria.pt (J.S.); ana.o.abreu@ipleiria.pt (I.O.A.); juliana.gaspar@ipleiria.pt (J.A.G.); susete.pinteus@ipleiria.pt (S.P.);

**Keywords:** skin, aminopyrimidine–arylthioether conjugates, antioxidant activity, antibacterial activity, photoprotective activity, anti-enzymatic activity

## Abstract

Skin functionalities are instrumental in four main domains: protection, regulation, sensation, and support. However, excessive exposure to ultraviolet (UV) radiation can compromise skin integrity and, in turn, affect its functions, by generating reactive oxygen species (ROS). Aiming to protect skin from UV radiation, sunscreens incorporate UV filters and antioxidants that absorb/reflect UV rays and neutralise free radicals, respectively. Nevertheless, undesired side and ecological effects of conventional UV filters have spurred the search for safer alternatives. Among synthetic antioxidants, thioethers have attracted attention for their redox power and potential medicinal properties. In this context, a library of aminopyrimidine–arylthioether conjugates was synthesised and evaluated for their antioxidant, enzyme-inhibitory and antibacterial activities, as well as for their cytotoxicity in HaCaT cells and potential photoprotective properties. Among the aminopyrimidine-thioethers studied, compound **C5** stood out for its antioxidant potential, exhibiting a value of 566.39 mM FeSO_4_ equivalents per mM of the compound, while compound **C2** showed the highest anti-enzymatic potential, inhibiting elastase (45.58%) and tyrosinase activities (34.66%). Regarding photoprotective activity, compound **C13** reduced by 33.74% the ROS production induced by UV radiation exposure, at 100 μM, a non-cytotoxic concentration. Finally, compound **C7** inhibited the growth of *Staphylococcus epidermidis*, *Staphylococcus hominis* and *Cutibacterium acnes*, at 30 μM. These preliminary results demonstrate that aminopyrimidine–arylthioethers constitute a new class of compounds warranting further investigation for skin protection. Compound C5 showed antioxidant activity in the FRAP assay, comparable to that of the positive control, BHT.

## 1. Introduction

With an annual income of 85.40 billion euros, the cosmetics business is a thriving and growing sector of the global economy, largely driven by multinational corporations [1]. In Europe, consumers allocate around 135 euros per year to cosmetic products, with consumption increasing with age. In highly developed nations such as Switzerland and Norway, expenses can increase up to 225 euros [2].

The cosmetics field encompasses a wide range of products, including skincare, haircare, makeup, fragrances and hygiene toiletries, such as deodorants, soaps and oral care products. Among all product domains, skincare has been in the spotlight of the cosmetics industry for its significant impact on beauty and well-being, economic returns, and, most importantly, skin health [3,4].

Pinpointed as the most extensive organ in the human body and a component of the integumentary system, the skin encompasses several roles. Mainly, it forms a protective barrier separating the body from the exterior, thereby preventing the entry of harmful particles and dangerous pathogens, and blocking ultraviolet (UV) light [5,6]. Among its functions, the skin controls temperature, sustains electrolyte balance and provides structural support [5].

Excessive exposure to harmful substances or environmental factors can lead to damage in skin health, ranging from skin irritations and burns to skin cancer. Among the causes, sunlight is the most significant factor contributing to these health issues. Specifically, the ultraviolet (UV) radiation that penetrates the skin may generate free radicals, mainly reactive oxygen species (ROS), that can impact health negatively in several ways, including by reacting with DNA, breaking down collagen, causing burns and inflammation, accelerating skin ageing and dramatically increasing the risk of cancer [7].

To minimise the effects of UV radiation, several measures can be taken, such as limiting exposure during the sunniest parts of the day and regularly applying a sunscreen. Sunscreens protect the skin because they incorporate chemical systems acting as filters, which absorb or reflect UV radiation [8]. Additionally, they may include antioxidants, which do not block UV rays directly but, instead, neutralise the free radicals that are generated on the skin upon UV exposure.

While commercial sunscreens have improved over time, there are rising concerns about the safety of UV filters, due to toxic effects on humans, including photoallergic reactions, contact sensitivity and endocrine level fluctuations. In addition, their accumulation in the environment, especially in aquifers, raises concerns for their impact on aquatic organisms. Specifically, some of the currently used UV filters, such as Avobenzone, are known to undergo rapid photoisomerisation concurrently with photodegradation under radiation exposure, increasing the concentration of the keto tautomer. Through a Norrish type I cleavage, phenacyl and benzoyl radicals may be formed [9,10]. These and other by-products, such as chlorinated acetophenones, are recognised as toxic to humans and the environment [10,11]. Octocrylene, another UV filter, can undergo retro-aldol condensation to form a phototoxic benzophenone derivative known for its carcinogenic properties, also acting as an endocrine disruptor [10,12]. To circumvent these downsides, the discovery and development of natural antioxidants have been in the spotlight. Commonly used natural antioxidants include vitamins C and E, polyphenols (such as those found in green tea, resveratrol, and ferulic acid), niacinamide, coenzyme Q10, carotenoids, and enzymatic antioxidants [7].

**Figure 1:**
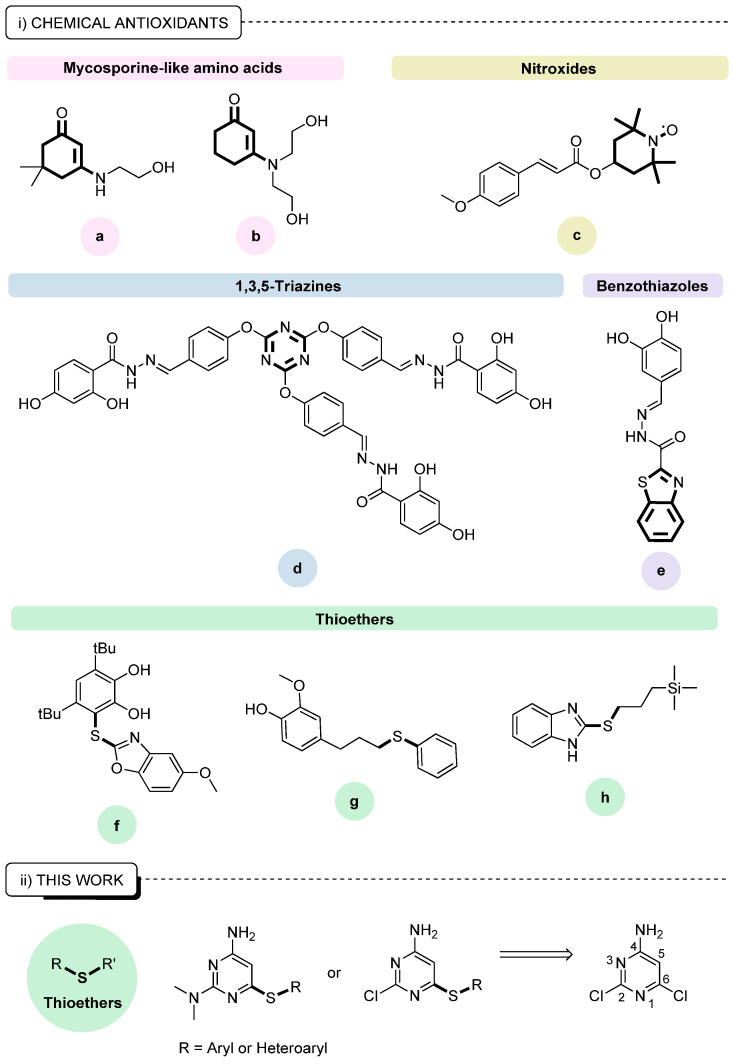
(**i**) Selected chemotypes with antioxidant properties: mycosporine-like amino acids (compounds **a** and **b**) [13], nitroxides (compound **c**) [14], 1,3,5-triazines (compound **d**) [15], benzothiazoles (compound **e**) [16], and thioethers (compounds **f**, **g** and **h**) [17,18,19]. (**ii**) Schematic representation of the synthesis pathway to access the aminopyrimidine-thioethers reported in this work.

Additionally, to broaden the range of antioxidants suitable for use in sunscreens, researchers have examined compounds featuring other chemical motifs, such as mycosporine-like amino acids (Figure 1i, compounds **a** and **b**) [13], nitroxide derivatives (Figure 1i, compound **c**) [14], 1,3,5-triazines (Figure 1i, compound **d**) [15], benzothiazoles (Figure 1i, compound **e**) [16], and thioethers (Figure 1i, compound **f** [17], **g** [18], and **h** [19]).

The antioxidant properties of sulphur-based compounds have attracted particular attention in the context of medicinal chemistry. Sulphur-based moieties are often found in the structural skeleton of molecules, featuring antiparasitic [20,21,22,23], anti-inflammatory [24], antiviral [25], antibacterial [26] and anticancer [27] drugs and drug candidates.

In view of these findings and the growing interest in thiolated compounds, we undertook the design and synthesis of a library of aminopyrimidine-thioethers (Figure 1ii) for further evaluation of their potential as antioxidants and of their inhibitory properties against collagenase, elastase, tyrosinase, and hyaluronidase enzyme activity. Moreover, we disclose the *in vitro* antibacterial activity of all compounds against the bacteria *Staphylococcus aureus*, *Staphylococcus epidermidis*, *Staphylococcus hominis*, and *Cutibacterium acnes*, their cytotoxicity in an immortalised human keratinocyte cell line (HaCaT cells), and their photoprotective properties.

## 2. Materials and Methods

### 2.1. Chemicals

All reagents used in the synthetic approaches applied were purchased from commercial entities and were not purified before used. Analytical thin-layer chromatography (TLC) was conducted using Merck TLC Silica gel 60F254 aluminium sheets (Darmstadt, Germany) and visualised under UV light, or with the appropriate stain. In this case, p-anisaldehyde and potassium permanganate were most commonly used. Column chromatography was executed using Sigma Aldrich technical-grade silica gel (Darmstadt, Germany) with a pore size of 60 Å, 230–400 mesh particle size, and 40–63 μm particle size.

### 2.2. Equipment

^1^H and ^13^C nuclear magnetic resonance (NMR) spectra were recorded on a 500 MHz JEOL system (Peabody, MA, USA) supplied with a Royal HFX probe, using the deuterated solvents pinpointed in each experimental procedure. The chemical shifts (δ) are written in parts per million (ppm), measured downfield from an internal standard of tetramethylsilane (TMS). Melting points (°C) were determined on an SMP30 melting point apparatus and are reported uncorrected. High-resolution mass spectrometry (HRMS) data were obtained with a Thermo Scientific Orbitrap Elite HRMS instrument (Waltham, MA, USA), capable of performing MSn up to *n* = 10. The NMR and HRMS facilities used are available at the Centre of Marine Sciences (CCMAR) analytical services, in the Algarve, Portugal.

### 2.3. Synthesis

***tert*****-Butyl -*****tert*****-butyl(2,6-dichloro-4-pyrimidinyl)(oxycarbonylamino)formylate** (**1**) **and *****tert*****-Butyl (2,6-dichloro-4-pyrimidinyl)carbamate** (**2**)

For the preparation *tert*-Butyl-*tert*-butyl(2,6-dichloro-4-pyrimidinyl)(oxycarbonylamino)formylate (**1**) and tert-Butyl (2,6-dichloro-4-pyrimidinyl)carbamate (**2**), the procedure described by Peng et al. [28] was adapted with slight modifications. 2,6-Dichloro-4-pyrimidinylamine (10.0 g, 61.0 mmol) and *N*,*N*-dimethylpyridin-4-amine (DMAP; 0.7 g, 6.1 mmol) were dissolved in dichloromethane (236.0 mL) and triethylamine (12.3 g, 17.0 mL, 122.0 mmol) was added, followed by addition of di-tert-butyldicarbonate (29.3 g, 134.2 mmol). The mixture was stirred overnight, at 0 °C, then distilled water was added. The organic layers were separated, washed with brine, dried over anhydrous MgSO_4_, and concentrated under reduced pressure. The obtained residue was purified by silica gel flash chromatography (ethyl acetate–hexane, 1:99, *v*/*v*), affording two compounds: compound 1 (white solid, 11.5 g, 52%); compound 2 (colourless crystals, 7.2 g, 45%). *tert-Butyl-tert-butyl(2,6-dichloro-4-pyrimidinyl)(oxycarbonylamino)formylate (**1**)*: M.p., 58–60 °C. (EtOAc-hexane, 1:99, *v*/*v*). ^1^H NMR (500 MHz, DMSO-*d*_6_): δ 7.85 (s, 1H), 1.50 (s, 18H) ppm. ^13^C{^1^H} NMR (126 MHz, DMSO-*d*_6_): δ 161.8, 159.8, 157.9, 148.6, 109.8, 85.5, 27.2 ppm. HRMS (ESI^+^, *m*/*z*) calcd for C_14_H_19_Cl_2_N_3_O_4_K (M + K)^+^: 402.03842; found: 402.03836. Diff: 0.15 ppm. *tert-butyl (2,6-dichloro-4-pyrimidinyl)carbamate (**2**)*: M.p., 120–122°C. (EtOAc-hexane, 1:99, *v*/*v*). ^1^H NMR (500 MHz, DMSO-*d*_6_): δ 11.15 (s, 1H), 7.83 (s, 1H), 1.47 (s, 9H) ppm. ^13^C{^1^H} NMR (126 MHz, DMSO-*d*_6_): δ 161.7, 161.1, 158.4, 151.9, 106.3, 81.9, 27.7 ppm. HRMS (ESI^+^, *m*/*z*) calcd for C_9_H_11_Cl_2_N_3_O_2_Na (M+Na)^+^: 286.01205; found: 286.01245. Diff: −1.40 ppm.

**General procedure 1: S-C cross coupling reaction through Buchwald–Hartwig methodology.** The syntheses of aminopyrimidine-thioethers were conducted in accordance with the procedure described by Colloti et al. [29], with slight modifications. To a stirring solution of bis(dibenzylideneacetone)palladium (Pd_2_(dba)_3_, 0.01 mmol), 1,1′-bis(diphenylphosphino)ferrocene (DPPF, 0.02 mmol), N,N-diisopropylethylamine (DIPEA, 1.0 mmol) and the thiol (1.0 mmol), in anhydrous dimethylformamide (DMF, 5.0 mL), compound **1** or **2** (1.0 mmol) was added. Depending on the thiol used, the mixture was stirred and refluxed for 6 h, 24 h, or 48 h. Then, the mixture was cooled to room temperature, treated with distilled water (50.0 mL) and extracted with dichloromethane (DCM) (3 × 50.0 mL). The organic layers were combined, washed with brine, dried over anhydrous MgSO_4_ and concentrated under reduced pressure. The obtained crude was purified through flash chromatography using an acetone–hexane gradient, yielding the desired pure aminopyrimidine-thioethers.

*2-Chloro-6-(p-fluorophenylthio)-4-pyrimidinylamine* (**C1**). This compound was synthesised following ***general procedure 1***, from reaction of compound **1** with *p*-fluorobenzenethiol, over 24 h. Purification by flash chromatography (acetone–hexane, 20:80, *v/v*) provided a solid. Recrystallisation from methanol yielded colourless crystals (0.3 g, 48% yield). M.p., 208–210 °C. ^1^H NMR (500 MHz, DMSO-*d*_6_): δ 7.72–7.68 (m, 2H), 7.43–7.38 (m, 2H), 7.31 (s, 2H), 5.65 (s, 1H) ppm. ^13^C{^1^H} NMR (126 MHz, DMSO-*d*_6_): δ 170.7, 164.8, 164.4, 162.4, 158.9, 138.4, 138.3, 123.2, 123.2, 117.5, 117.3, 97.2 ppm. HRMS (ESI^+^, *m/z*) calcd C_10_H_8_ClFN_3_S (M+H)^+^: 256.01060; found 256.01047. Diff: 0.51 ppm.

*6-(Benzylthio)-2-chloro-4-pyrimidinylamine* (**C2**). This compound was synthesised following ***general procedure 1***, from reaction of compound **1** with 4-phenylmethanethiol, over 24 h. Purification by flash chromatography (acetone–hexane, 15:85, *v/v*) yielded a solid. Recrystallisation from methanol yielded white crystals (0.2 g, 15% yield). M.p., 174–176 °C. ^1^H NMR (500 MHz, DMSO-*d*_6_): δ 7.42–7.39 (m, 2H), 7.34–7.29 (m, 4H), 7.27–7.24 (m, 1H), 6.24 (s, 1H), 4.31 (s, 2H) ppm. ^13^C{^1^H} NMR (126 MHz, DMSO-*d*_6_): δ 167.9, 164.4, 158.9, 137.0, 128.9, 128.5, 127.3, 98.0, 32.9 ppm. HRMS (ESI^+^, *m/z*) calcd C_11_H_11_ClN_3_S (M+H)^+^: 252.03567; found 252.03558. Diff: 0.36 ppm.

*2-Chloro-6-(5-methyl-1,3,4-thiadiazol-2-ylthio)-4-pyrimidinylamine* (**C3**). This compound was synthesised following ***general procedure 1***, from reaction of compound **1** with 5-methyl-1,3,4-thiadiazole-2-thiol, over 24 h. Purification by flash chromatography (acetone–hexane, 15:85, *v/v*) yielded a white solid (0.1 g, 15% yield). M.p., 250–252 °C (dec.). ^1^H NMR (500 MHz, DMSO-*d*_6_): δ 7.71–7.55 (m, 2H), 6.31 (s, 1H), 2.79 (s, 3H) ppm. ^13^C{^1^H} NMR (126 MHz, DMSO-*d*_6_): δ 170.1, 164.8, 163.3, 159.1, 156.9, 99.5, 15.5 ppm. HRMS (ESI^+^, *m/z*) calcd C_7_H_7_ClN_5_S_2_ (M+H)^+^: 259.98259; found 259.98218. Diff: 1.58 ppm.

*2-Chloro-6-(1-methyl-1H-tetraazol-5-ylthio)-4-pyrimidinylamine *(**C4**). This compound was synthesised following ***general procedure 1***, from reaction of compound **1** with 1-methyl-1*H*-tetrazole-5-thiol, over 24 h. Purification by flash chromatography (acetone–hexane, 15:85, *v/v*) yielded a white solid (0.1 g, 14% yield). M.p., 234–236 °C (dec.). ^1^H NMR (500 MHz, DMSO-*d*_6_): δ 7.62 (d, *J* = 48.2 Hz, 2H), 6.15 (s, 1H), 4.06 (s, 3H) ppm. ^13^C{^1^H} NMR (126 MHz, DMSO-*d*_6_): δ 164.9, 162.7, 159.2, 147.2, 99.0, 34.7 ppm. HRMS (ESI^+^, *m/z*) calcd C_6_H_6_ClN_7_NaS (M+Na)^+^: 265.99861; found 265.99857. Diff: 0.15 ppm.

*6-(p-Aminophenylthio)-2-chloro-4-pyrimidinamine * (**C5**). This compound was synthesised following ***general procedure 1***, from reaction of compound **1** with *p*-aminobenzenethiol, over 24 h. Purification by flash chromatography (acetone–hexane, 26:74, *v/v*) yielded a white solid (0.5 g, 20% yield). M.p., 181–183 °C. ^1^H NMR (500 MHz, DMSO-*d*_6_): δ 7.26–7.19 (m, 4H), 6.65 (d, *J* = 8.1 Hz, 2H), 5.70 (s, 2H), 5.63 (s, 1H) ppm. ^13^C{^1^H} NMR (126 MHz, DMSO-*d*_6_): δ 173.6, 164.7, 158.7, 151.0, 137.1, 115.0, 110.1, 96.5 ppm. HRMS (ESI^+^, *m/z*) calcd C_10_H_10_ClN_4_S (M+H)^+^: 253.03092; found 253.03084. Diff: 0.32 ppm.

*N-[p-(6-Amino-2-chloro-4-pyrimidinylthio)phenyl]acetamide* (**C6**). This compound was synthesised following ***general procedure 1***, from reaction of compound **1** with *N-p*-Mercaptophenylacetamide, over 24 h. Purification by flash chromatography (acetone–hexane, 18:82, *v/v*) afforded a light-yellow solid. Recrystallisation from ethyl acetate and hexane yielded light-yellow crystals (1.0 g, 45% yield). M.p., 214–216 °C. ^1^H NMR (500 MHz, DMSO-*d*_6_): δ 10.24 (s, 1H), 7.77–7.73 (m, 2H), 7.56–7.53 (m, 2H), 5.63 (s, 1H), 2.08 (s, 3H) ppm. ^13^C{^1^H} NMR (126 MHz, DMSO-*d*_6_): δ 171.6, 168.9, 164.7, 158.9, 141.3, 136.6, 120.1, 96.9, 24.1 ppm. HRMS (ESI^+^, *m/z*) calcd C_12_H_11_ClN_4_OSNa (M+Na)^+^: 317.02343; found 317.02313. Diff: 0.95 ppm.

*N-[p-(6-Amino-2-chloro-4-pyrimidinylthio)phenyl]bromoacetamide* (**C7**). The procedure described by Stampolaki et al. [30] was followed, with slight modifications. To a stirred solution of compound **C5** (2.0 g, 7.9 mmol), K_2_CO_3_ (1.4 g, 10 mmol), water (14 mL) and tetrahydrofuran (THF, 40 mL), at 0 °C, was added dropwise to a solution of bromoacetyl chloride (1.4 g, 0.7 mL, 8.7 mmol) in THF (22 mL). The final mixture was then left to stir at room temperature, until consumption of the starting material. The final reaction mixture was washed with DCM (3 × 20 mL), then the organic layers were combined and concentrated under reduced pressure. The resulting solid was dissolved in diethyl ether and washed with aqueous NaHCO_3_ (10% *w/v),* water, aqueous HCl 3%, water, and brine. The organic layers were concentrated under reduced pressure, affording a colourless solid (2.7 g, 92% yield). M.p., 186–188 °C. (dec.) ^1^H NMR (500 MHz, DMSO-*d*_6_): δ 10.57 (s, 1H), 7.65 (d, *J* = 7.9 Hz, 2H), 7.51 (dd, *J* = 8.7, 2.3 Hz, 2H), 7.26 (s, 2H), 6.17 (s, 1H), 4.06 (s, 2H) ppm. ^13^C{^1^H} NMR (126 MHz, DMSO-*d*_6_): δ 171.4, 165.7, 164.9, 158.1, 140.1, 136.5, 123.7, 120.2, 99.8, 30.9 ppm. HRMS (ESI^+^, *m/z*) calcd C_12_H_10_BrClN_4_OSK (M+K)^+^: 412.90583; found 412.90546. Diff: 0.90 ppm.

*2-Chloro-6-(2-pyridylthio)-4-pyrimidinylamine* (**C8**). This compound was synthesised following ***general procedure 1***, from reaction of compound **2** with 2-pyridinethiol, over 6 h. Purification by flash chromatography (acetone–hexane, 10:90, *v/v*) gave a solid. Recrystallisation from ethyl acetate and hexane yielded the required product as colourless crystals (0.6 g, 44% yield). M.p., 183–185 °C. ^1^H NMR (500 MHz, DMSO-*d*_6_): δ 8.65 (dd, *J* = 4.9, 1.8 Hz, 1H), 7.90 (td, *J* = 7.8, 1.9 Hz, 1H), 7.70 (d, *J* = 7.9 Hz, 1H), 7.46 (dd, *J* = 7.5, 4.8 Hz, 1H), 7.40 (s, 2H), 6.17 (s, 1H) ppm. ^13^C{^1^H} NMR (126 MHz, DMSO-*d*_6_): δ 167.1, 164.8, 159.0, 152.0, 150.8, 138.3, 128.9, 123.8, 99.9 ppm. HRMS (ESI^+^, *m/z*) calcd for C_9_H_8_ClN_4_S (M+H)^+^: 239.01527; found: 239.01546. Diff: 0.79 ppm.

*4-Amino-2-(dimethylamino)-6-(phenylthio)pyrimidine* (**C9**). This compound was synthesised following ***general procedure 1***, from reaction of compound **1** with benzenethiol, over 48 h. Purification by flash chromatography (acetone–hexane, 20:80, *v/v*) afforded a powder. Recrystallisation from ethyl acetate yielded colourless crystals (0.2 g, 17% yield). M.p., 116–118 °C. ^1^H NMR (500 MHz, DMSO-*d*_6_): δ 7.57 (ddt, *J* = 5.5, 2.7, 1.5 Hz, 2H), 7.50–7.46 (m, 3H), 6.29 (s, 2H), 5.16 (s, 1H), 2.96 (s, 6H) ppm.^13^C{^1^H} NMR (126 MHz, DMSO-*d*_6_): δ 168.0, 163.6, 161.3, 135.5, 129.6, 129.4, 129.2, 88.6, 36.4 ppm. HRMS (ESI^+^, *m/z*) calcd for C_12_H_15_N_4_S (M+H)^+^: 247,10119; found: 247.10086. Diff: 1.34 ppm.

*4-Amino-2-(dimethylamino)-6-(2-pyridylthio)pyrimidine* (**C10**). This compound was synthesised following ***general procedure 1***, from reaction of compound **1** with 2-pyridinethiol, over 48 h. Purification by flash chromatography (acetone–hexane, 10:90, *v/v*) gave a solid. Recrystallisation from ethyl acetate yielded yellow crystals (0.2 g, 18% yield). M.p., 171–173 °C. ^1^H NMR (500 MHz, DMSO-*d*_6_): δ 8.57–8.54 (m, 1H), 7.81 (td, *J* = 7.7, 2.0 Hz, 1H), 7.67 (dd, *J* = 7.9, 1.1 Hz, 1H), 7.35 (ddd, *J* = 7.4, 4.8, 1.1 Hz, 1H), 6.42 (s, 2H), 5.58 (s, 1H), 2.96 (s, 6H) ppm. ^13^C{^1^H} NMR (126 MHz, DMSO-*d*_6_): δ 164.4, 163.8, 161.5, 154.3, 150.1, 137.4, 128.4, 122.7, 91.4, 36.4 ppm. HRMS (ESI^+^, *m/z*) calcd for C_11_H_14_N_5_S (M+H)^+^: 248.09644; found: 248.09593. Diff: 2.06 ppm.

*5-(6-Amino-2-chloro-4-pyrimidinylamino)-1,3,4-thiadiazole-2-thiol* (**C12**). This compound was synthesised following ***general procedure 1***, from reaction of compound **1** with 5-amino-1,3,4-thiadiazole-2-thiol, over 24 h. Purification by flash chromatography (acetone–hexane, 20:80, *v/v*) afforded a beige solid (0.4 g, 9%). M.p., 244–246 °C. (dec.) ^1^H NMR (500 MHz, DMSO-*d*_6_): δ 8.59 (s, 1H), 7.60 (d, *J* = 10.5 Hz, 2H), 7.11 (s, 1H), 6.32 (s, 1H) ppm. ^13^C{^1^H} NMR (126 MHz, DMSO-*d*_6_): δ 164.5, 161.6, 160.5, 160.3, 157.3, 152.9, 100.7 ppm. HRMS (ESI^+^, *m/z*) calcd C_6_H_5_ClN_6_S_2_K^+^ (M+K)^+^: 298,93372; found 298.93433. Diff: −2.04 ppm.

*2-Chloro-6-(phenylthio)-4-pyrimidinylamine* (**C13**). This compound was synthesised following ***general procedure 1***, from reaction of compound **1** with benzenethiol, over 24 h. Purification by flash chromatography (acetone–hexane, 20:80, *v/v*) gave a solid residue. Recrystallisation from ethyl acetate and hexane yielded colourless crystals of the required product (1.0 g, 77% yield). M.p., 229–231 °C. ^1^H NMR (500 MHz, DMSO-*d*_6_): δ 7.64–7.62 (m, 2H), 7.57–7.53 (m, 3H), 7.32 (s, 2H), 5.69 (s, 1H) ppm. ^13^C{^1^H} NMR (126 MHz, DMSO-*d*_6_): δ 170.7, 164.7, 158.9, 135.6, 130.4, 130.3, 127.4, 97.2 ppm. HRMS (ESI^+^, *m/z*) calcd for C_10_H_9_ClN_3_S (M+H)^+^: 238.02002; found: 238.01993. Diff: 0.38 ppm.

**Antioxidant capacity assessment aminopyrimidine-thioether conjugates**

The antioxidant capacity of the aminopyrimidine-thioethers under investigation was evaluated according to Luz et al. (2025) [31], using different approaches, namely the 2,2-diphenyl-1-picrylhydrazyl (DPPH) radical scavenging ability, the ferric reducing antioxidant power (FRAP) and the superoxide scavenging radical activity. DPPH and FRAP assays used 2,6-di*tert*-butyl-4-methylphenol butylated hydroxytoluene, (BHT; 100 μM), as the standard and superoxide scavenging radical activity used quercetin (100 μM).

### 2.4. 2,2-Diphenyl-1-Picrylhydrazyl (DPPH) Radical Scavenging Activity

The ability of aminopyrimidine-thioethers (100 μM) to neutralize the DPPH free radical was assessed using the method described by Brand-Williams et al. (1995) [32]. Following 30 min of dark incubation, at room temperature, with a DPPH solution (0.1 mM), the absorbance was recorded at 517 nm. DMSO served as a negative control. Results were shown as a percentage compared to the control.

### 2.5. Ferric Reducing Antioxidant Power

The FRAP assay was conducted following the method described by Benzie and Strain, [33,34] with small modifications. The FRAP reagent (300 mM acetate buffer, pH 3.6, 10 mM TPTZ in 40 mM HCl, and 20 mM FeCl_3_ in a 10:1:1 ratio) was held at 37 °C. Samples were mixed with the reagent and kept in the dark, at room temperature for 30 min. Absorbance was measured at 593 nm, using a microplate reader (Epoch Microplate Spectrophotometer, BioTek®, Winooski, V T, USA). A standard curve of FeSO_4_ (0–10 mM) was used, and outcomes were reported as μM FeSO_4_ equivalents per μM of compound.

### 2.6. Superoxide Scavenging Radical

The activity of scavenging superoxide radicals was assessed using NADH (557 µM), PMS (450 µM), and NBT (108 µM) in 1.6 × 10^4^ µM Tris-HCl buffer (pH 8). The aminopyrimidine-thioethers (100 μM) were combined with the reagents and incubated for 5 min, after which absorbance was recorded at 560 nm (Epoch Microplate Reader, BioTek^®^). DMSO served as the negative control. Results were present as a percentage compared to the control.

**Enzymatic inhibitory activity of synthetic aminopyrimidine-thioether conjugates**

The inhibitory effects of aminopyrimidine-thioethers on the activity of collagenase (Type IV), elastase, tyrosinase and hyaluronidase enzymes were evaluated according with Susano et al., 2021[35] .

### 2.7. Collagenase and Elastase Activity

The inhibitory effects of the aminopyrimidine-thioethers (100 μM) on collagenase and elastase activities were assessed using the *EnzChek® Gelatinase/Collagenase Assay Kit* (E12055, Invitrogen™, ThermoFisher Scientific, Waltham, MA, USA) and the *EnzChek® Elastase Assay Kit* (E12056, Invitrogen™, ThermoFisher Scientific, Waltham, MA, USA), respectively, in line with the guidelines provide by the manufacturer’s instructions. Enzyme activities were calculated from the slope of the fluorescence kinetics from fluorescein-conjugated substrate cleavage. Epigallocatechin gallate (EGCG; 10.5 μM and 262 μM, respectively) served as a positive control, and results were expressed as a percentage relative to the control.

### 2.8. Tyrosinase Activity

Tyrosinase inhibition was assessed following Lee et al. (2009) [35] and Sezer Senol et al. (2016) [36] using aminopyrimidine-thioethers (100 μM) in potassium phosphate buffer (0.5 mM, pH 6.8) and L-DOPA (1 mM) at 37 °C for 5 min, followed by tyrosinase addition (50 U/mL). Absorbance at 475 nm was measured for 15 min. Kojic acid (141 μM) was used as positive control, and results were expressed as tyrosinase activity (% of control).

### 2.9. Hyaluronidase Activity

The inhibitory activity of the aminopyrimidine-thioethers (100 μM) against hyaluronidase activity was assessed based on Yahaya and Don (2012) [37], with minor modifications. Compounds were pre-incubated with hyaluronidase (7 U/mL) for 10 min at 37 °C, followed by the addition of hyaluronic acid to initiate the reaction. After incubation (45 min at 37 °C), unreacted substrate was precipitated using an acidic albumin solution, during 10 min at room temperature, and absorbance was measured at 600 nm. Epigallocatechin gallate (EGCG; 262 μM) was used as a positive control, and results were expressed as a percentage of the control.

***In vitro*** **photoprotective activity of the aminopyrimidine-thioether conjugates**

### 2.10. Cell Culture Maintenance

Human keratinocytes (HaCaT-300493; Cytion biobank) were cultured in high-glucose DMEM with phenol red, supplemented with 10% foetal bovine serum (FBS) and 1% antibiotic/antimycotic solution (Biowest, Nuaillé, France), under standard conditions (37 °C, 5% CO_2_, 95% humidity). Cells were subcultured at 80–85% confluence using 0.05% EDTA (10 min), followed by 0.05% trypsin (0.05%)−0.025% EDTA (5 min). After centrifugation (5 min, 200 g, 25 °C), cells were resuspended in fresh medium and seeded into culture flasks or 96-well plates for subsequent assays.

### 2.11. Cytotoxic Activity

The cytotoxicity of aminopyrimidine-thioethers was evaluated using the 3-[4,5-dimethylthiazol-2-yl]-2,5-diphenyltetrazolium bromide (MTT) colorimetric assay [38]. HaCaT cells seeded in 96-well plates were treated with the compounds (30–100 μM) for 24 h. DMSO (1%) and saponin (2 mg/mL) were used as negative and positive controls for cell death, respectively. Cells were then incubated with MTT solution (Sigma-Aldrich, Steinheim, Germany) (100 μL; 1.2 mM) for 1 h under formazan crystals solubilised with DMSO and incubated in the dark at room temperature. Absorbance was measured at 570 nm using a microplate reader (Epoch Microplate Spectrophotometer, BioTek,Winooski, VT, USA), and results were expressed as a percentage of untreated control cells.

### 2.12. Photoprotective Capacity

The photoprotective potential of compounds was assessed by measuring intracellular ROS levels in HaCaT cells using the fluorophore 2′,7′-dichlorodihydrofluorescein diacetate (H_2_DCFDA) [39]. HaCaT cells were pre-treated with non-cytotoxic concentrations of the compounds for 1 h, followed by exposure to UVA-B radiation (7.9 mW/cm^2^; 15 min). After radiation, cells were washed with Hank’s balanced salt solution and incubated with 10 μM H_2_DCFDA, for 30 min in the dark. ROS levels were determined by fluorescence measurement (Multimodal Synergy H1, BioTek^®^ Instruments, Winooski, VT, USA) at excitation of 495 nm and emission of 527 nm wavelengths. Ascorbic acid (100 μM) was used as positive control, and results were expressed as a percentage of the control.

**Antimicrobial activity of aminopyrimidine-thioether conjugates against skin disorder-related microorganisms**

The antimicrobial activity of the aminopyrimidine-thioethers was evaluated against representative skin microbiota, including Gram-positive bacteria (*Staphylococcus aureus* DSM 1104, *Staphylococcus hominis* DSM 20328, *Staphylococcus epidermidis* DSM 1798) and *Cutibacterium acnes* (DSM 1897). Bacterial strains were cultured under optimal conditions: *S. aureus*, *S. hominis* and *S. epidermidis* in tryptic soy broth supplemented with 0.3% yeast extract (TSYE) at 37 °C; and *C. acnes* in tryptic soy broth under anaerobic conditions at 37 °C. Bacterial suspension were adjusted to 0.5 McFarland and incubated with compounds (30–100 μM). Antimicrobial activity was assessed during the exponential growth phase through optical density measurement at 600 nm (Epoch Microplate Spectrophotometer, BioTek, USA). DMSO was used as a negative control and oxytetracycline as a positive control. Growth inhibition was expressed as percentage relative to the negative control (100% growth) [40].

### 2.13. Data Statistical Analysis

All experiments were performed at least three times independently, with each condition tested in triplicate, and results are expressed as mean ± standard error of the mean (SEM). Statistical significance was set at *p*-value < 0.05. Differences between treated groups and the control were assessed using one-way analysis of variance (ANOVA) followed by Dunnett’s post hoc test. Data distribution and variance homogeneity were verified using Shapiro–Wilk and Levene test, respectively. Half-maximal effective concentration EC50 and half maximal inhibition concentration (IC50) values were calculated by nonlinear regression using GraphPad Prism (version 10.01), applying a sigmoidal dose-response model. All statistical analyses and graphical outputs were generated using GraphPad Prism software v.9.1 (GraphPad Software, La Jolla, CA, USA).

## 3. Results

### 3.1. Synthesis

**Scheme 1:**
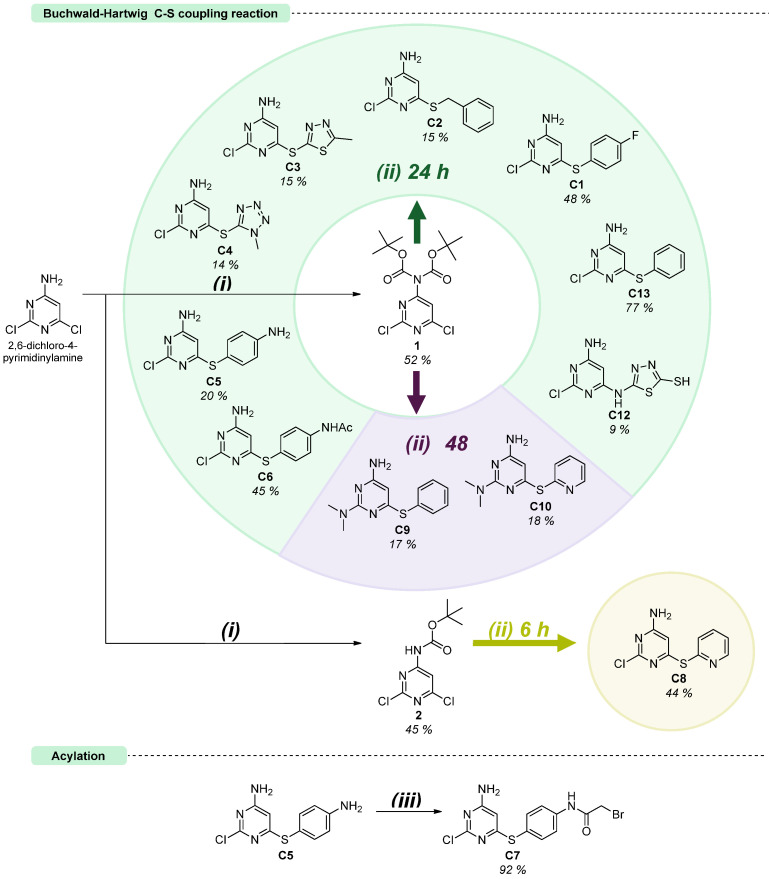
Approach to the synthesis of aminopyrimidine-thioether conjugates. [Reaction conditions: (i) Boc_2_O, DMAP, TEA, DCM, 0 °C, on.; and (ii) HS-R, Pd_2_(dba)_3_, DPPF, DIPEA, DMF, reflux (iii) ClCOCH_2_Br, K_2_CO_3_, DCM, H_2_O, 0 °C-r.t., o./n].

The synthetic strategy followed in the preparation of the aminopyrimidine-thioethers is depicted in Scheme 1 and has been reported, together with unanticipated reactivity features encountered along the synthesis [41]. The chosen synthetic approach focused on varying the heterocyclic moiety in the right arm of the thioether while maintaining an aminopyrimidine core structure on the left arm. The introduction of variations on the right side of the final targets enables us to get information regarding the effect of the heterocyclic group on the antioxidant activity, providing a basis for further optimisation. It involves a Buchwald–Hartwig C-S cross-coupling reaction, using DPPF as a bidentate phosphine ligand and the catalyst Pd_2_(dba)_3_ as a palladium source [29,42]. Aminopyrimidine-thioether synthesis departed from 2,6-dichloro-4-pyrimidinylamine as the initial substrate, starting with protection of the amino group by reaction with di-tert-butyl dicarbonate (Boc_2_O). This step prevented the dimerisation of the initial pyrimidine and enhanced the solubility of the Boc-protected derivatives in DMF, compared to the starting material, 2,6-dichloropyrimidin-4-amine. Two products were isolated from this step, containing one or two tert-butyloxycarbonyl (Boc) protecting groups (**1** and **2**, Scheme 1), which were used as building blocks in the coupling with the selected thiols. To optimise the conditions for a Buchwald–Hartwig reaction, we evaluated the effect of amine protection on the yield of compound C13, which was synthesised by reacting thiophenol with three different derivatives: the unprotected aminopyrimidine and the monoBoc- and diBoc-protected derivatives (compounds 2 and 1, respectively), over 24 h. Compound C13 was obtained with yields of 15% from the unprotected aminopyrimidine, 35% from compound 2, and 77% from compound 1. The higher yield observed when using substrate 1 can be attributed to the electron-withdrawing effects of the two Boc groups, which decrease the electron density of the pyrimidine ring, thereby increasing the susceptibility of the C6 position to nucleophilic attack by thiophenol. Given these observations, compound 1 was mostly used for further Buchwald–Hartwig reactions. Overall, the prior amine protection strategy notably improved the yields of the target molecules. Of note, the conditions employed for the coupling reaction also mediate the removal of the Boc groups, thus eliminating the need for an additional deprotection step before isolating the desired product. Noteworthily, during these reactions, we observed the formation of different products depending on the reaction time. After 24 h of reaction, we obtained the product from coupling of 2,6-dichloro-4-pyrimidinylamine with the thiol at C6. However, leaving the reaction for over 48 h afforded an aryl sulphide product where the chloride attached to C2 was displaced by the dimethylamino moiety C9, supplied by DMF, although such observation was not transversal to all compounds. For the reaction of compound 1 with 2-mercaptopyridine, over 24 h, we observed the formation of both products (C8 and C10). Then, to obtain only compound C8 (with the chloride attached to C2), we used compound 2 as the starting material, reducing the reaction time to 6 h, since the C2 position in compound 2 appears to be less susceptible to nucleophilic attack than in compound 1. To obtain the corresponding compound with a tertiary amine at C2 (compound C10), we reacted compound 1 with 2-mercaptopyridine, over 48 h. Moreover, the preparation of compound C6 demanded a prior amine protection of the *p*-aminobenzenethiol (data not shown, achieved by acetylation. On the other hand, compound C5 underwent acylation to yield compound C7. Structurally, C7 contains a carbon with high electrophilic character attached to the bromide atom and thus is highly susceptible to nucleophilic attack. From an antimicrobial perspective, this feature could enhance its effectiveness. An enzymatic nucleophilic attack could readily displace the bromide, forming a covalent adduct that could interfere with the normal activity of the bacterial enzyme. The twelve conjugates obtained, as depicted in Scheme 1, were used in the present work. Characterisation details of the compounds are available as Supplementary Information.

### 3.2. Cytotoxic Activities on HaCaT Cells

**Figure 2:**
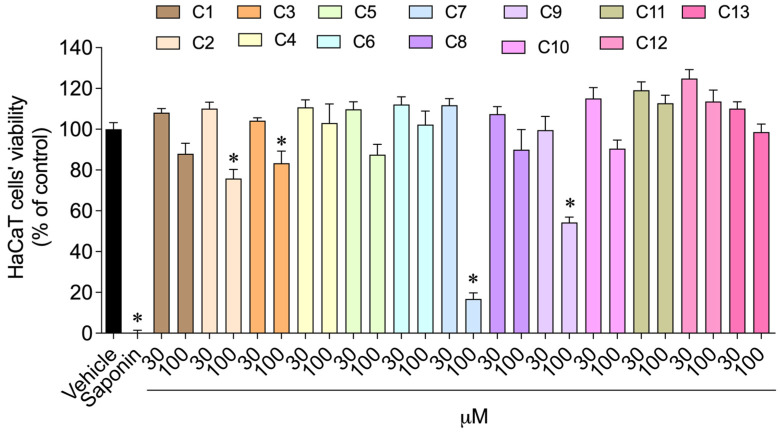
Cytotoxicity of compounds (**C1**–**C13**) (30–100 μM) on HaCaT cells’ viability, after 24 h exposure. Values in each column represent the mean ± SEM of three independent experiments carried out in triplicate. Symbol (*) represents significant differences (ANOVA, Dunnet’s test, *p* < 0.05) when compared to the vehicle.

The cytotoxicity of aminopyrimidine-thioethers (C1–C13) on HaCaT cells’ viability was estimated using the MTT method, and the results are presented in Figure 2.

As observed in Figure 2, compounds **C2**, **C3**, **C7** and **C9** significantly reduced HaCaT cells’ viability by 20–80%, at a concentration of 100 μM. On the other hand, the remaining compounds did not exhibit a significant toxicity effect, compared to the vehicle. In view of these results, non-toxic concentrations were subsequently employed in the photoprotective assay, ensuring safety while maintaining reliable results.

### 3.3. Antioxidant Capacity

To evaluate the antioxidant capacity of the aminopyrimidine-thioether compounds, a set of *in vitro* assays (DPPH, FRAP, and SRSA) were performed. First, the 2,2-diphenyl-1-picrylhydrazyl (DPPH) radical scavenging assay quantifies the compound’s ability to scavenge free radicals [43,44,45]. In DPPH radicals, the single electron on the nitrogen atom is reduced to the corresponding hydrazine (DPPH-H) upon accepting a hydrogen atom from the antioxidants [43,44,45]. Overall, in the presence of hydrogen donors, the formed DPPH-H generates a violet/purple colour that can be quantified photometrically [43,44,45]. Unlike the DPPH assay, the FRAP method does not involve scavenging added radicals. Instead, it relies on a redox reaction that converts ferric-tripyridyltriazine (Fe^3+^-TPTZ) mediated by the compound to ferrous-tripyridyltriazine (Fe^2+^-TPTZ). During the assay, once the ferric iron is reduced, a blue colour emerges through the presence of the FRAP indicator, which can be measured photometrically [44,45]. Finally, the superoxide anion (O_2_^•−^) is a prior ROS that serves as a precursor to a vast range of ROS responsible for cellular damage and is generated by the addition of one electron to oxygen [46,47]. The generation of O_2_^•-^ can be measured by the reduction of phenazine methosulfate (PMS) in the presence of NADH. The formed radical, in turn, reduces the nitroblue tetrazolium (NBT) indicator to a blue-coloured formazan that can be measured photometrically [46,47]. Noteworthily, in the presence of compounds that can scavenge O_2_^•−^, less formazan will be formed [46,47]. Results are presented in Table 1.

Table 1 discloses the results of antioxidant evaluations of aminopyrimidine-thioethers employing the DPPH, FRAP, and SRSA assays. Considering the data from DPPH and superoxide scavenging capacity, none of the compounds demonstrated antioxidant activity in any of the assays employed. However, data from the FRAP assay has shown that compound **C5** exhibited the highest antioxidant potential, with a value of 566.39 ± 29.87 mM FeSO_4_ equivalents per mM of the compound.

### 3.4. Enzymatic Activity

The inhibitory potential of compounds on the enzymes collagenase, elastase, tyrosinase and hyaluronidase was also evaluated for non-cytotoxic concentrations, and the results are shown in Table 2.

As evidenced in Table 2, compound **C2** exhibited the highest anti-enzymatic potential, by significantly inhibiting the activity of elastase (45.58 ± 7.07%) and tyrosinase (34.66 ± 2.97%). However, these inhibitory effects were lower than those of the reference standards, EGCG (100.00 ± 11.80%) and kojic acid (55.09 ± 2.91%), respectively. Compounds **C4** and **C5** also reduced tyrosinase activity by 31.32% and 27.20%, respectively. Concerning hyaluronidase activity, compounds **C13**, **C1**, and **C6** showed inhibitory capacities ranging from 24.00% to 28.00%. Finally, none of the compounds inhibited collagenase activity.

### 3.5. Photoprotective Activity

**Figure 3:**
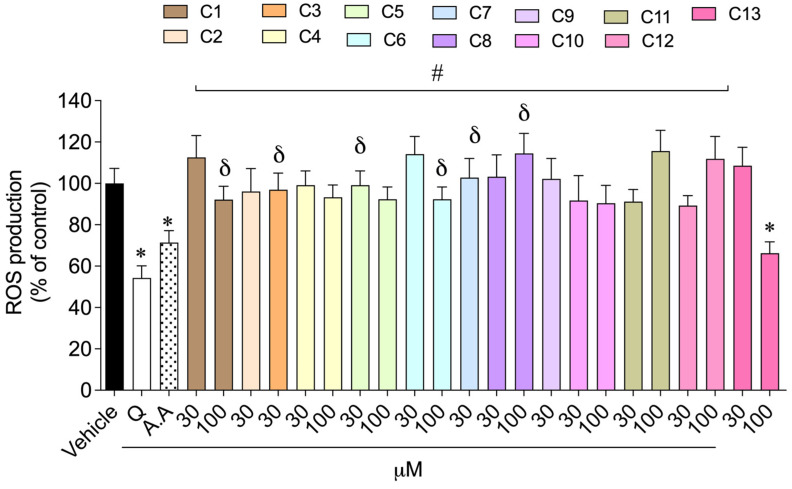
Production of reactive oxygen species by HaCaT cells exposed to UV radiation (7.9 mW/cm^2^, 15 min), in the presence of compounds (**C1**–**C13**) (non-toxic concentrations; 30–100 μM). Values in each column represent the mean ± SEM of three independent experiments carried out in triplicate. Symbols represent significant differences (ANOVA, Dunnet’s test, *p* < 0.05) when compared (*) to the vehicle, (#) to the quercetin (Q, 100 μM) and (δ) to the ascorbic acid (A.A, 100 μM).

The photoprotective capacity of synthetic aminopyrimidine-thioethers (**C1**–**C13**) on HaCaT cells exposed to UV radiation was assessed through the production of ROS, at non-cytotoxic concentrations, and the results are depicted in Figure 3.

As shown in Figure 3, compound **C13** reduced the ROS production induced by UV radiation exposure by 33.74% of ROS, at 100 μM.

### 3.6. Antimicrobial Activity Against Skin Microorganisms

**Figure 4:**
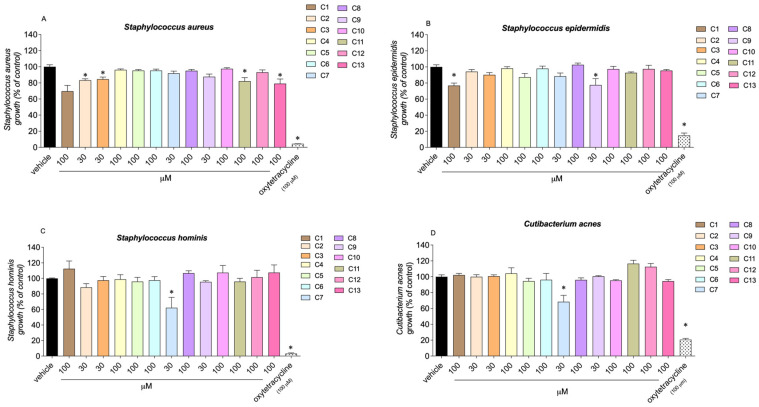
Antimicrobial activity of compounds (C1–C13) (30–100 μM) against *Staphylococcus aureus* (**A**), *Staphylococcus epidermidis* (**B**), *Staphylococcus hominis* (**C**) and *Cutibacterium acnes* (**D**). Values in each column represent the mean ± SEM of three independent experiments carried out in triplicate. Symbol (*) represents significant differences (ANOVA, Dunnet’s test, *p* < 0.05) when compared to the vehicle.

The antimicrobial effects of synthetic aminopyrimidine-thioethers (**C1**–**C13**) were analysed against four microorganisms related to skin disorders, at non-cytotoxic concentrations (30–100 μM): Gram (+) bacteria, *S. aureus*, *S. hominis*, *S. epidermidis* and *C. acnes*. Results are presented in Figure 4.

Compound **C7** exhibited the highest antimicrobial activity, inhibiting the growth of *S. hominis*, and *C. acnes* at 30 µM (Figure 4). Compounds **C2** and **C3** showed the greatest capacity to reduce *S. aureus* growth, at 30 µM (83.30 ± 1.76% and 84.55 ± 2.65%, respectively). Compounds **C1** (76.78 ± 3.14%) and **C9** (77.47 ± 7.95%) reduced bacterial growth of *S. epidermidis* by 25–30%, at 100 µM and 30 µM, respectively. In the case of *S. hominis*, only compound **C7** was able to reduce bacterial growth at 30 µM (62.15 ± 13.24%) by 40%. Regarding *C. acnes*, only compound **C7** reduced its growth by 40%, at 30 µM (66.23 ± 8.14%).

## 4. Discussion

The antioxidant properties of sulphur-based compounds have garnered significant interest in medicinal chemistry, attributed to the redox nature of the sulphur atom. In this work we report the synthesis and characterisation of a library of aminopyrimidine-thioethers, four of which have already been disclosed [41]. The thioethers were prepared by coupling di- or mono-Boc-protected 2,6-dichloro-4-pyrimidinylamine (compounds **1** and **2**, respectively) with an aryl or heteroaryl thiol, using the Buchwald–Hartwig C-S cross-coupling methodology. Two particularities were found in these reactions: the first corresponds to the dimethylamino-substitution of chloride at C2 of the pyrimidine scaffold after 48 h of reaction, whereas within 24 h the chloride linked to that position is conserved; the second relates to the need to adjust reaction conditions, based on the thiol’s reactivity. For almost all the target compounds bearing the chloride atom at C2, we used compound **1** and a reaction time not exceeding 24 h. Of note, employing the same conditions for the thiol 2-mercaptopyridine led to the formation of a mixture of two products. To obtain only compound **C8**, with a chloride substituent at C2, we used milder conditions, reacting compound **2** for 6 h. On the other hand, to obtain compound **C10**, bearing a dimethylamino substituent at C2, we used compound **1** and extended the reaction for 48 h.

With the library of aminopyrimidine-thioethers in hand we proceeded to further in vitro studies, starting with a cytotoxicity test in HaCaT cells. This viability test allows the determination of the safe concentration range for each compound to be used in subsequent tests. Most of the thioethers tested proved non-toxic at the higher concentration of 100 µM. The exceptions were compounds **C2**, **C3**, **C7**, and **C9**, which reduced HaCaT cell viability within a range of 20.00% to 80.00%, at 100 μM. Among these compounds **C7** was the most toxic, probably due to the presence of a bromide substituent within its structure that could act as a good nucleofuge, easily removed upon attack by nucleophilic moieties of biomolecules, e.g., proteins.

The antioxidant activity was scrutinised through three assays: DPPH, FRAP, and SRSA. The first assay evaluates the DPPH radical scavenging activity; the second evaluates the ferric reducing antioxidant power; and the third assesses the superoxide anion radical scavenging activity. All thioethers were tested at non-toxic concentrations: 100 µM for all compounds, except **C2**, **C3**, **C7**, and **C9**, which were tested at 30 µM. Hydroxytoluene (BHT) was used as a positive control. Results showed that, generally, the compounds demonstrated no antioxidant activity in any of the assays employed. The exception is compound **C5**, which exhibited the highest antioxidant potential via the FRAP assay, with a value of 566.39 mM FeSO_4_ equivalents per mM of the compound. Structurally, compound **C5** is a thioether containing an aminothiophenol moiety. Its redox-active sulphur atoms and its electron-rich heterocyclic system may explain the compound’s ability to reduce Fe^3+^ to Fe^2+^. Indeed, all compounds contain a sulphur atom in their structure. However, in the case of **C5** the amine can improve the reducing power by exerting an electron-donating effect, thereby increasing electron density on the aromatic ring and, in turn, making the molecule easier to oxidise. Additionally, sulphur is highly polarisable, also stabilising oxidised species. Overall, the electron-donating effect of the amino group, together with the stabilising effect of the sulphur atom, corroborate the higher reducing power of **C5**. In fact, Jun Bo He et al. [48] reported a study on the antioxidant activity of sulphur-containing diphenylamines, proposing an intramolecular synergistic mechanism between amino and sulphur moieties that accounts for the antioxidant activity of these structures.

Equally, the inhibitory potential of the thioethers on the enzymes collagenase, elastase, tyrosinase, and hyaluronidase was evaluated. From a cosmetic perspective, these are important enzymes to consider due to their roles in skin ageing and antioxidant stress. Collagenase [49] and elastase [50] degrade collagen and elastin fibres, respectively, leading to loss of skin structural integrity. Hyaluronidase [51] degrades hyaluronic acid, reducing skin hydration, while tyrosinase [52] plays a central role in melanin biosynthesis and pigmentation disorders. Therefore, inhibiting these enzymes is an important strategy in anti-ageing and dermatological research. Unfortunately, no compound stood out by exhibiting strong inhibitory effects on any of these four enzymes, and all compounds showed effects below those of the positive controls, kojic acid and epigallocatechin gallate. Of note, compound **C2** exhibited the highest anti-enzymatic potential, inhibiting elastase by 45.58%, yet it was below the 100.00% inhibition observed with kojic acid.

To gather additional data on the antioxidant effects of this bank of thioethers, assessments of ROS production in HaCaT cells exposed to UV radiation were performed. Compound **C13** demonstrated the greatest reduction in ROS production after UV irradiation at 100 μM, achieving a 66.26% ROS production, comparable to the effect observed with the positive control, quercetin. As mentioned above, the sulphur atom plays an essential role in redox processes. Apart from electron transfer, thereby reducing iron, sulphur can also act as a ROS scavenger. In other words, sulphur can undergo oxidation into sulfoxide or sulfone during ROS decomposition. These findings align with those of Davies et al. [53], who demonstrated that biological sulphur-containing residues, such as methionine, were oxidised in the presence of singlet oxygen.

Finally, the antimicrobial activity of all compounds against four Gram (+) bacteria associated with skin disorders, *S. aureus*, *S. hominis*, *S. epidermidis* and *C. acnes*, was assessed for non-cytotoxic concentrations defined in HaCaT human cells. Among the thioethers, compound **C7** exhibited the highest antimicrobial activity, particularly against *S. hominis* and *C. acnes*. As mentioned for cytotoxic studies, the activity of compound **C7** may be ascribed to the high electrophilic character of the carbon atom attached to the bromide, which favours its action against several molecular host targets in a non-selective way. On the other hand, *S. epidermidis* growth was inhibited by compounds **C1** and **C9**, which can be related to the presence of a *para*-fluorine atom at the benzyl group (**C1**) and a tertiary amine functionality in position C2 of the aminopyrimidine scaffold (**C9**). Indeed, Bhoi et al. [54] reported the synthesis and in silico evaluation of the antiplasmodial and antimicrobial activities of a set of thymol-based benzimidazoles, and their analysis of structure–activity relationships highlighted that fluorine substitution at the *para* position of the aromatic ring is associated with enhanced antimicrobial and antiplasmodial potency [54,55]. In addition, tertiary amines have been shown to exhibit antibacterial activity by damaging organelles, specifically mitochondria and the membrane. Dong et al. [56] published a study on the antimicrobial activity of a tertiary amine-based chitosan. In their haemolysis rate evaluations, they observed that primary amines are more cytotoxic than tertiary amines and also disclosed that tertiary amines displayed enhanced antimicrobial activity against a set of Gram-positive and Gram-negative bacteria and fungi. This enhanced activity may be attributed to easier protonation of tertiary amines, which favours retention, especially in acidic organelles where they exert their function. Further mechanistic studies revealed that tertiary amines induce ROS accumulation and enhance mitochondrial membrane depolarisation in *C. albicans* cells, pointing to the mitochondria as a target. Concerning *S. aureus*, compounds **C2** and **C3** were able to reduce this microorganism’s growth. In the case of compound **C2**, the inclusion of a -CH_2_- linker provides conformational flexibility, which may enhance solubility, increasing activity [57,58]. On the other hand, compound **C3** contains a methylthiadiazole group, known for its antibacterial properties [59].

## 5. Conclusions

Give the redox properties of the sulphur atom, sulphur-based compounds have been under scrutiny for their potential antioxidant properties, of significant interest in medicinal chemistry. In this context, we report the synthesis and characterisation of a library of aminopyrimidine-thioethers obtained through Buchwald–Hartwig C-S cross-coupling reactions. Succinctly, reaction outcomes depended on thiol reactivity and time of reaction. Shorter reaction times (up to 24 h) allowed for retention of the C2-chloro substituent, whereas prolonged reactions (48 h) promoted chloride substitution by a dimethylamino group provided by the solvent, dimethylformamide. Most target compounds retaining chlorine at C2 were obtained from precursor **1**, after 24 h. Exceptionally, the Buchwald–Hartwig C-S cross-coupling reaction with 2-mercaptopyridine required tailored conditions. The reaction of compound **2** for 6 h afforded compound **C8**, while formation of the dimethylamino-based analogue (**C10**) required extending the reaction of precursor **1** for 48 h. Based on the antioxidant character of the sulphur atom, preliminary studies were conducted to evaluate the cytotoxicity, as well as the antioxidant, anti-enzymatic and antimicrobial activities of these thiolates, envisaging applications in the skincare domain. In general, cytotoxicity assessments proved the safety of this library of thioethers. However, compounds **C2**, **C3**, **C7** and **C9** reduced HaCaT cells’ viability by 20.00–80.00% at a concentration of 100 μM. Among these four compounds, **C7** proved to be the most toxic, probably due to the presence of an electrophilic sp^3^ carbon linked to bromide, highly susceptible to nucleophilic attack. Hence, DPPH, FRAP, and SRSA assays were performed to determine the antioxidant activity of the thioethers. Additionally, these compounds were evaluated for their enzyme inhibition activity (collagenase, elastase, tyrosinase, and hyaluronidase), antibacterial activity (*S. aureus*, *S. hominis*, *S. epidermidis*, and *C. acnes*), and potential for photoprotective properties. Although none of the synthesised compounds outperformed the positive controls in the biological assays, this library of aminopyrimidine–thioethers displayed antioxidant potential. In particular, compound **C13** reduced UV-induced ROS production (33.74%), showing an effect comparable to quercetin, thus corroborating the relevance of this chemotype in photoprotective antioxidant responses. Also, compound **C5** showed comparable reducing activity to the positive control, hydroxytoluene, supporting the ability of these compounds to act also as iron-reducing agents. Structurally, **C5** and **C13** share a common aminopyrimidine–thioether skeleton, suggesting that the sulphur core is in the spotlight for antioxidant activity. Moreover, **C5** features a particularity that justifies its pronounced reducing activity: the incorporation of a para-amino group on the benzyl moiety. Such a substituent enhances electronic density, facilitating iron reduction. In comparison, **C13** equilibrates lipophilicity through the unsubstituted benzyl moiety. Overall, these findings indicate that balancing lipophilicity and incorporating electron-donating substituents may contribute to enhancing antioxidant activity, probably by increasing redox potential, radical stabilisation, and cellular permeability. Further structure–activity optimisations should concentrate on two major aspects. Firstly, enhancing the iron-reducing potential, which may be achieved through functionalisation of the benzyl group with stronger electron donors, such as -OH, -OR, and substituted amines. As mentioned earlier, these groups are expected to increase electronic density at the sulphur atom, which, in turn, favours electron transfer, culminating in the reduction of iron. On the other hand, modulating lipophilicity through benzyl functionalisation or linker variation may improve cellular uptake, thereby boosting ROS scavenging efficacy. In conclusion, none of the produced aminopyrimidine–thioether compounds showed the ability to scavenge superoxide or DPPH radicals. However, compound C5 exhibited an iron-reducing power (FRAP) comparable to that of the positive control, BHT. Overall, the promising antioxidant properties of C5 support the inclusion of the aminopyrimidine–thioether chemotype in further studies to identify optimised molecules that could be developed as potential skincare candidates.

## Figures and Tables

**Table 1:** Antioxidant capacity of synthetic organic aminopyrimidine-thioether compounds (**C1**–**C13**) estimated by 2,2 diphenyl-1-picrylhydrazyl (DPPH) radical scavenging activity, ferric reducing antioxidant power (FRAP), and superoxide anion radical scavenging activity assays. All samples were tested at non-toxic concentrations, which was 100 µM for all the compounds except **C2**, **C3**, **C7** and **C9**, which were tested at 30 µM. BHT and quercetin were used as positive control.

Compound	DPPH Radical Scavenging Activity ^a^ (μM)	FRAP ^b^ (mM FeSO_4_/mM of Compound)	Superoxide Radical Scavenging Activity ^a^ (μM)
**C1**	>100	29.86 ± 2.10 ***	>100
**C2**	>100	31.72 ± 1.74 ***	>100
**C3**	>100	31.52 ± 1.73 ***	>100
**C4**	>100	31.65 ± 1.70 ***	>100
**C5**	>100	566.39 ± 29.87 ***	>100
**C6**	>100	33.53 ± 2.17 ***	>100
**C7**	>100	34.96 ± 2.53 ***	>100
**C8**	>100	31.75 ± 2.14 ***	>100
**C9**	>100	32.52 ±1.60 ***	>100
**C10**	>100	32.82 ± 1.96 ***	>100
**C11**	>100	32.92 ± 1.35 ***	>100
**C12**	>100	35.52 ± 2.61 ***	>100
**C13**	>100	32.85 ± 2.10 ***	>100
**BHT**	>100	768.16 ± 19.56	-
**Quercetin**	-	-	52.60 (45.66–61.21)

^a^ Radical scavenging activity (EC_50_ μM). ^b^ mM FeSO_4_/mM of compound; BHT and quercetin was used as positive control. Symbols represent significant differences when compared to positive control in FRAP (Mann–Whitney: *** *p* < 0.001).

**Table 2:** Inhibitory activity (% of inhibition) of compounds (**C1**–**C13**) and positive controls epigallocatechin gallate (EGCG) against collagenase (10.5 µM), elastase (262 µM), and hyaluronidase activities (262 µM) and kojic acid against tyrosinase (141 µM). All samples were tested at non-toxic concentrations, which was 100 µM for all the compounds except **C2**, **C3**, **C7** and **C9**, which were tested at 30 µM (*).

Compound	Elastase (% of Inhibition)	Collagenase (% of Inhibition)	Tyrosinase (% of Inhibition)	Hyaluronidase (% of Inhibition)
**C1**	27.68 ± 15.71	4.9 ± 4.27	3.32 ± 0.73	25.70 ± 8.15
C2 *	45.58 ± 7.07	1.73 ± 1.80	34.66 ± 2.97	0.00 ± 7.79
C3 *	0.00 ± 3.10	5.15 ± 3.56	0.00 ± 4.80	10.45 ± 1.09
**C4**	0.00 ± 3.93	7.24 ± 5.75	31.32 ± 2.71	23.7 ± 8.14
**C5**	0.00 ± 5.60	4.16 ± 4.88	27.20 ± 5.63	22.90 ± 6.31
**C6**	0.00 ± 2.11	6.18 ± 5.64	0.00 ± 16.42	24.10 ± 2.94
C7 *	15.30 ± 6.60	6.07 ± 2.71	0.00 ± 5.40	6.33 ± 1.16
**C8**	0.00 ± 5.85	13.5 ± 3.19	1.92 ± 0.58	2.21 ± 1.48
C9 *	0.00 ± 11.68	6.04 ± 2.74	0.00 ± 7.09	21.10 ± 1.59
**C10**	0.00 ± 3.21	5.43 ± 3.13	1.14 ± 4.12	19.6 ± 2.67
**C11**	0.00 ± 2.15	2.41 ± 1.02	24.63 ± 2.68	0.55 ± 1.76
**C12**	0.00 ± 3.46	4.85 ± 2.12	23.04 ± 1.90	2.15 ± 3.18
**C13**	11.13 ± 7.20	1.04 ± 0.60	2.51 ± 2.02	28.00 ± 4.72
**Kojic acid**	-	-	55.09 ± 2.91	-
**EGCG**	100.00 ± 11.80	98.13 ± 0.60	-	92.82 ± 5.07

## Data Availability

The original contributions presented in this study are included in the article/Supplementary Materials. Further inquiries can be directed to the corresponding author(s).

## Supplementary Materials

The following supporting information can be downloaded at: https://www.mdpi.com/article/10.3390/antiox15070841/s1, S1: ^1^H, ^13^C{^1^H} NMR spectra of the synthesised compounds. S2: HRMS spectra of the synthesised compounds. Figure S1: ^1^H NMR spectrum (500 MHz) of compound 1 in DMSO-*d_6_*. Figure S2: ^13^C{^1^H} NMR spectrum (126 MHz) of compound 1 in DMSO-*d_6_*. Figure S3: ^1^H NMR spectrum (500 MHz) of compound 2 in DMSO-*d_6_*. Figure S4: ^13^C{^1^H} NMR spectrum (126 MHz) of compound 2 in DMSO-*d_6_*. Figure S5: ^1^H NMR spectrum (500 MHz) of compound C1 in DMSO-*d_6_*. Figure S6: ^13^C{^1^H} NMR spectrum (126 MHz) of compound C1 in DMSO-*d_6_*. Figure S7: ^1^H NMR spectrum (500 MHz) of compound C2 in DMSO-*d_6_*. Figure S8: ^13^C{^1^H} NMR spectrum (126 MHz) of compound C2 in DMSO-*d_6_*. Figure S9: ^1^H NMR spectrum (500 MHz) of compound C3 in DMSO-*d_6_*. Figure S10: ^13^C{^1^H} NMR spectrum (126 MHz) of compound C3 in DMSO-*d_6_*. Figure S11: ^1^H NMR spectrum (500 MHz) of compound C4 in DMSO-*d_6_*. Figure S12: ^13^C{^1^H} NMR spectrum (126 MHz) of compound C4 in DMSO-*d_6_*. Figure S13: ^1^H NMR spectrum (500 MHz) of compound C5 in DMSO-*d_6_*. Figure S14: ^13^C{^1^H} NMR spectrum (126 MHz) of compound C5 in DMSO-*d_6_*. Figure S15: ^1^H NMR spectrum (500 MHz) of compound C6 in DMSO-*d_6_*. Figure S16: ^13^C{^1^H} NMR spectrum (126 MHz) of compound C6 in DMSO-*d_6_*. Figure S17: ^1^H NMR spectrum (500 MHz) of compound C7 in DMSO-*d_6_*. Figure S18: ^13^C{^1^H} NMR spectrum (126 MHz) of compound C7 in DMSO-*d_6_*. Figure S19: ^1^H NMR spectrum (500 MHz) of compound C8 in DMSO-*d_6_*. Figure S20: ^13^C{^1^H} NMR spectrum (126 MHz) of compound C8 in DMSO-*d_6_*. Figure S21: ^1^H NMR spectrum (500 MHz) of compound C9 in DMSO-*d_6_*. Figure S22: ^13^C{^1^H} NMR spectrum (126 MHz) of compound C9 in DMSO-*d_6_*. Figure S23: ^1^H NMR spectrum (500 MHz) of compound C10 in DMSO-*d_6_*. Figure S24: ^13^C{^1^H} NMR spectrum (126 MHz) of compound C10 in DMSO-*d_6_*. Figure S25: ^1^H NMR spectrum (500 MHz) of compound C12 in DMSO-*d_6_*. Figure S26: ^13^C{^1^H} NMR spectrum (126 MHz) of compound C12 in DMSO-*d_6_*. Figure S27: ^1^H NMR spectrum (500 MHz) of compound C13 in DMSO-*d_6_*. Figure S28: ^13^C{^1^H} NMR spectrum (126 MHz) of compound C13 in DMSO-*d_6_*.
