# Supplementary material for: Exploring the Dermocosmetic Value of Synthetic Aminopyrimidine-Thioethers
Source: Antioxidants (Basel). 2026 Jul 3;15(7):841. doi: 10.3390/antiox15070841 (PMC13405498; doi:10.3390/antiox15070841)

Supporting Information

Uncovering the Dermocosmetic Value of Synthetic Aminopyrimidine-Thioethers

Inês C. C. Costa[1,2], Joana Silva[3], Isabel Oliveira Abreu[3], Juliana Gaspar[3], Susete Pinteus[3], Celso Alves[3], Maria L. S. Cristiano[1,2]* and Rui Pedrosa[3,*]

1 Centro de Ciências do Mar do Algarve, CCMAR, Campus de Gambelas, University of Algarve, 8005-139 Faro, Portugal
2 Department of Chemistry and Pharmacy, Faculty of Sciences and Technology, Campus de Gambelas, University of Algarve, 8005-139, Faro, Portugal
3 MARE – Marine and Environmental Sciences Center /ARNET – Aquatic Research, ESTM, Polytechnic University of Leiria, 2520 – 630, Peniche, Portugal
* Correspondence: rui.pedrosa@ipleiria.pt; mcristi@ualg.pt

Table of contents

S1. ^{1}H, $^{13}C\{^{1}H\}$ NMR spectra of the synthesised compounds S2
S2. HRMS spectra of the synthesised compounds S32

S1. ^1H, $^{13}C\{^1H\}$ NMR spectra of the synthesised compounds

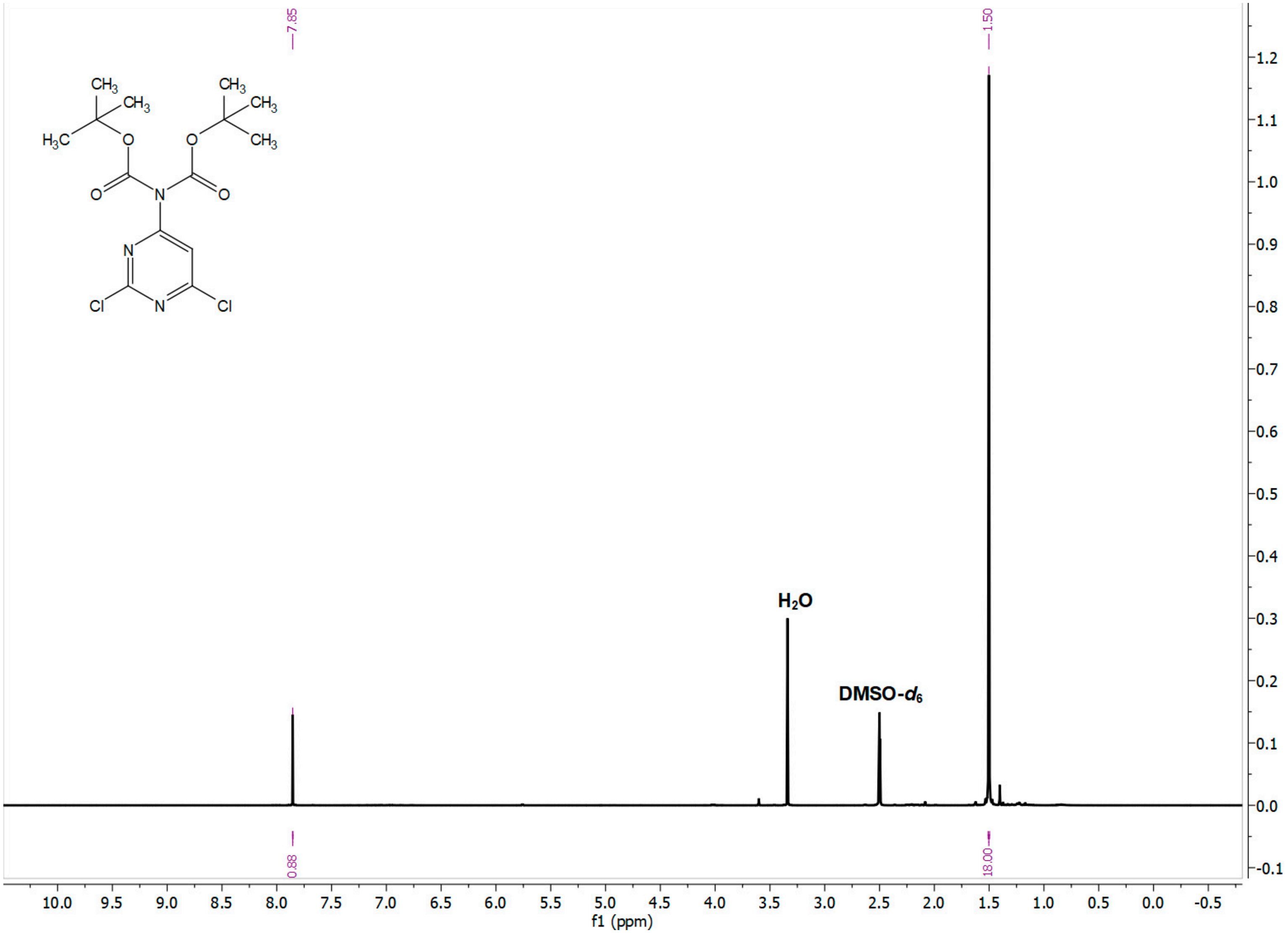

Figure S1. ^1H NMR spectrum (500 MHz) of compound **1** in DMSO-d_6.

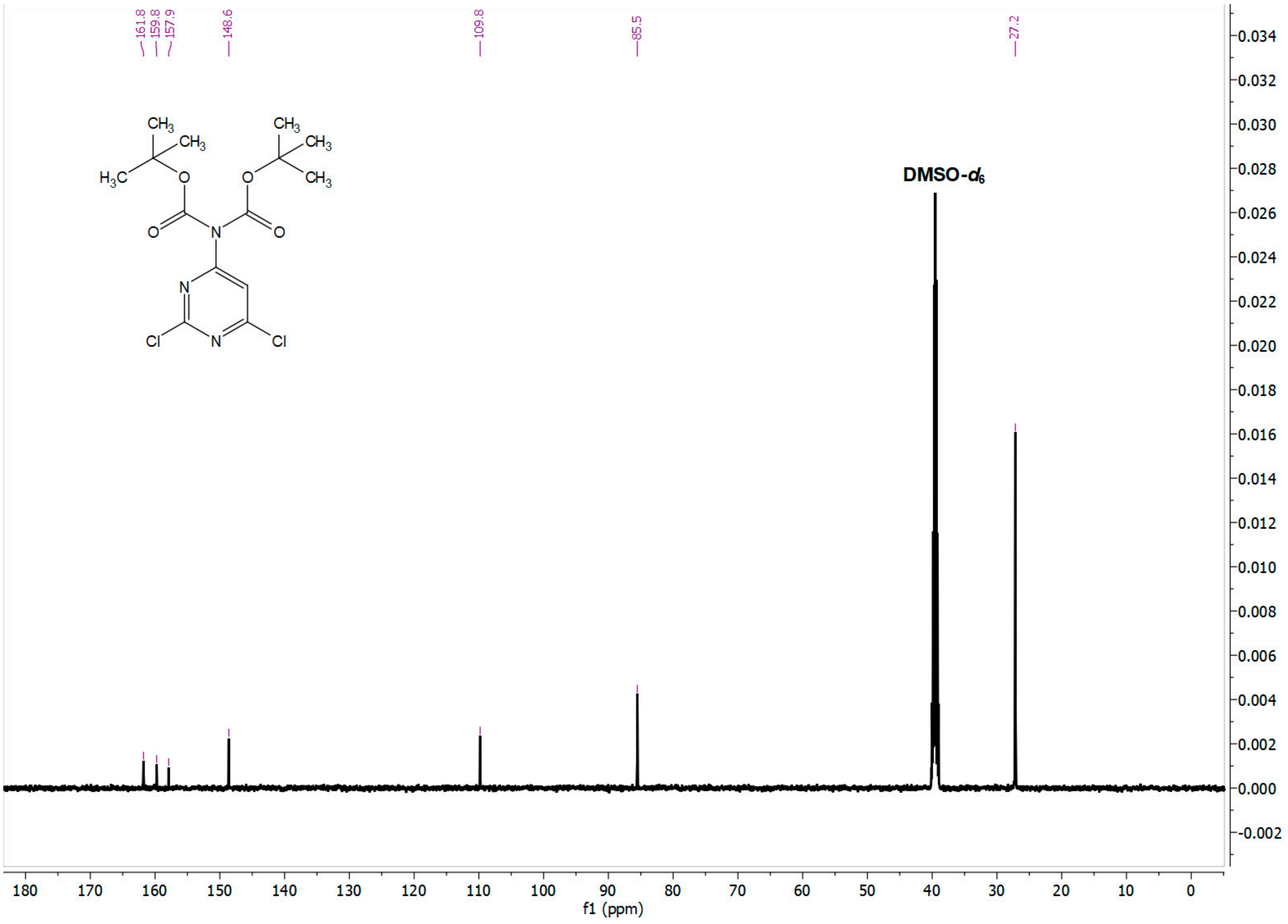

Figure S2. $^{13}C\{^1H\}$ NMR spectrum (126 MHz) of compound **1** in DMSO-*d*6.

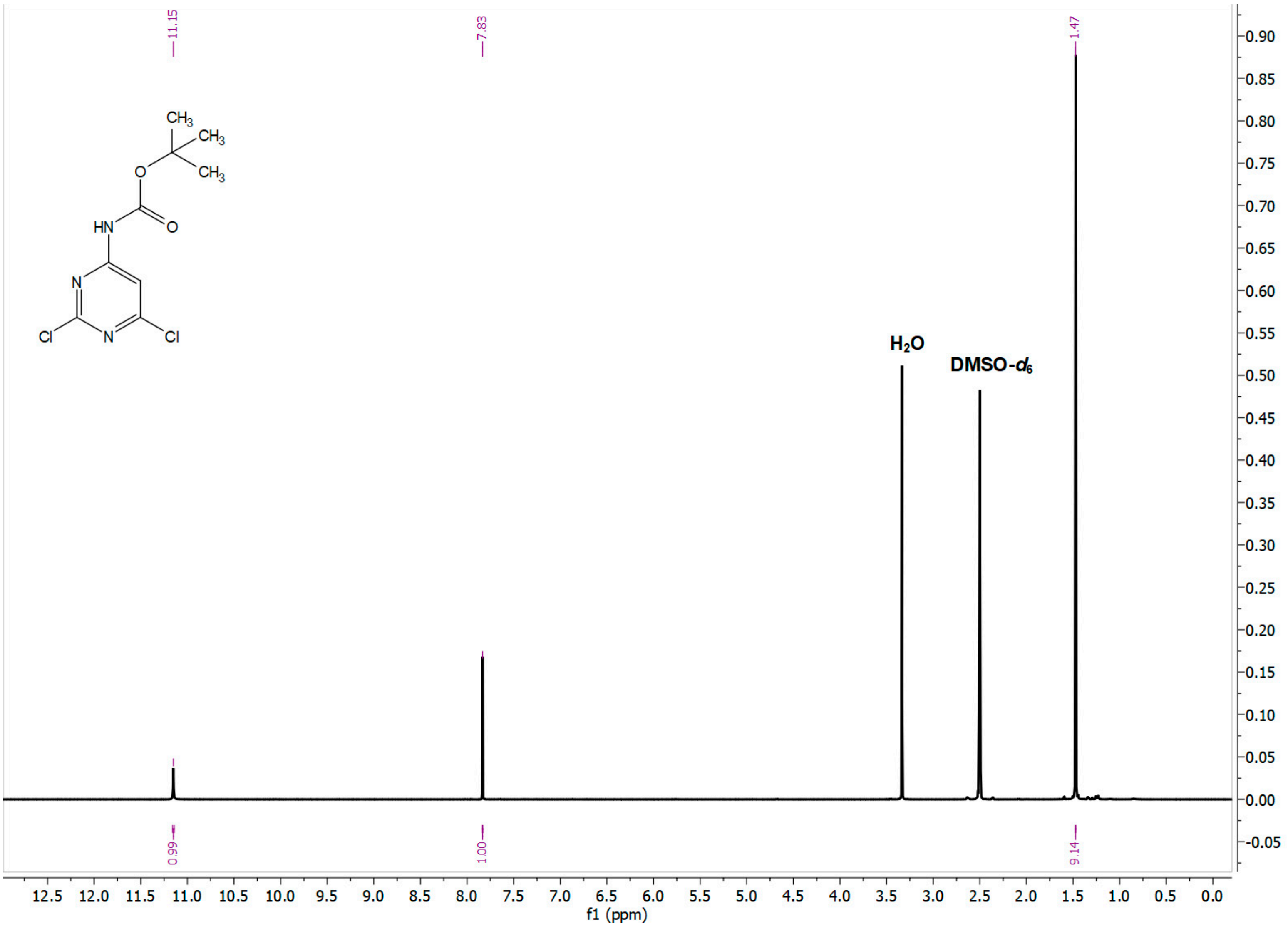

Figure S3. ^{1}H NMR spectrum (500 MHz) of compound **2** in DMSO-*d*$_6$.

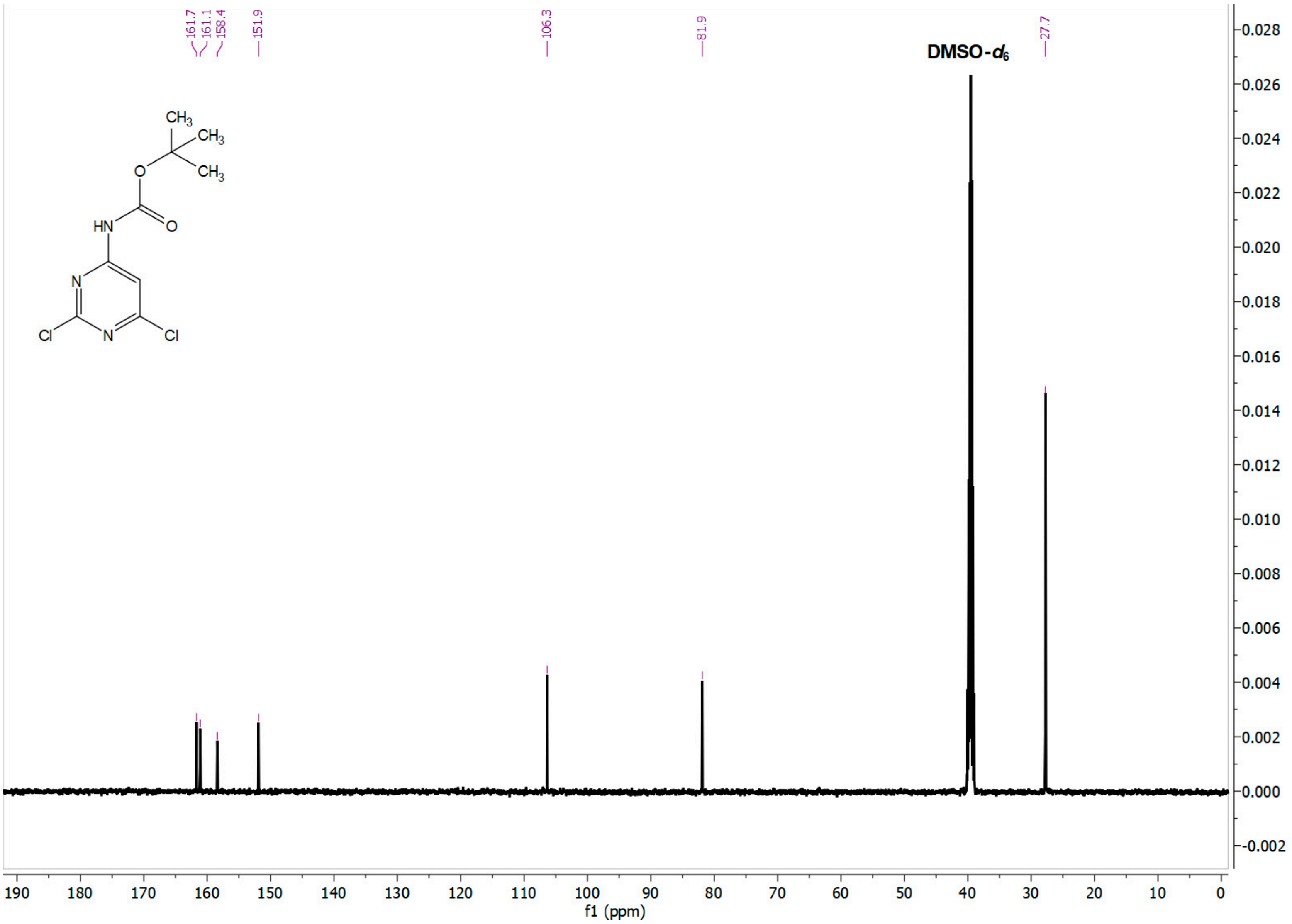

Figure S4. $^{13}C\{^1H\}$ NMR spectrum (126 MHz) of compound **2** in DMSO-d_6.

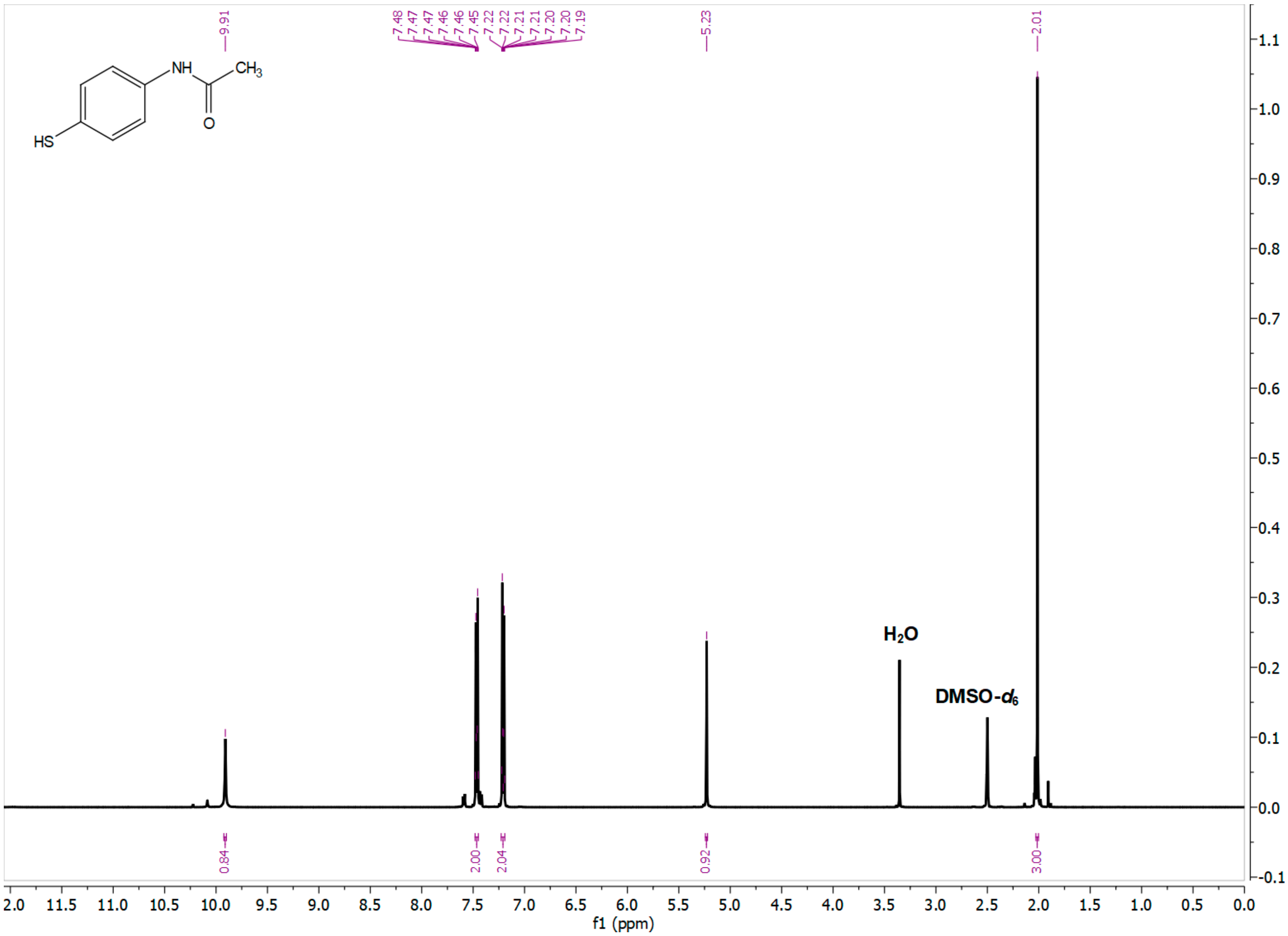

Figure S5. ^{1}H NMR spectrum (500 MHz) of compound **3** in DMSO-*d*6.

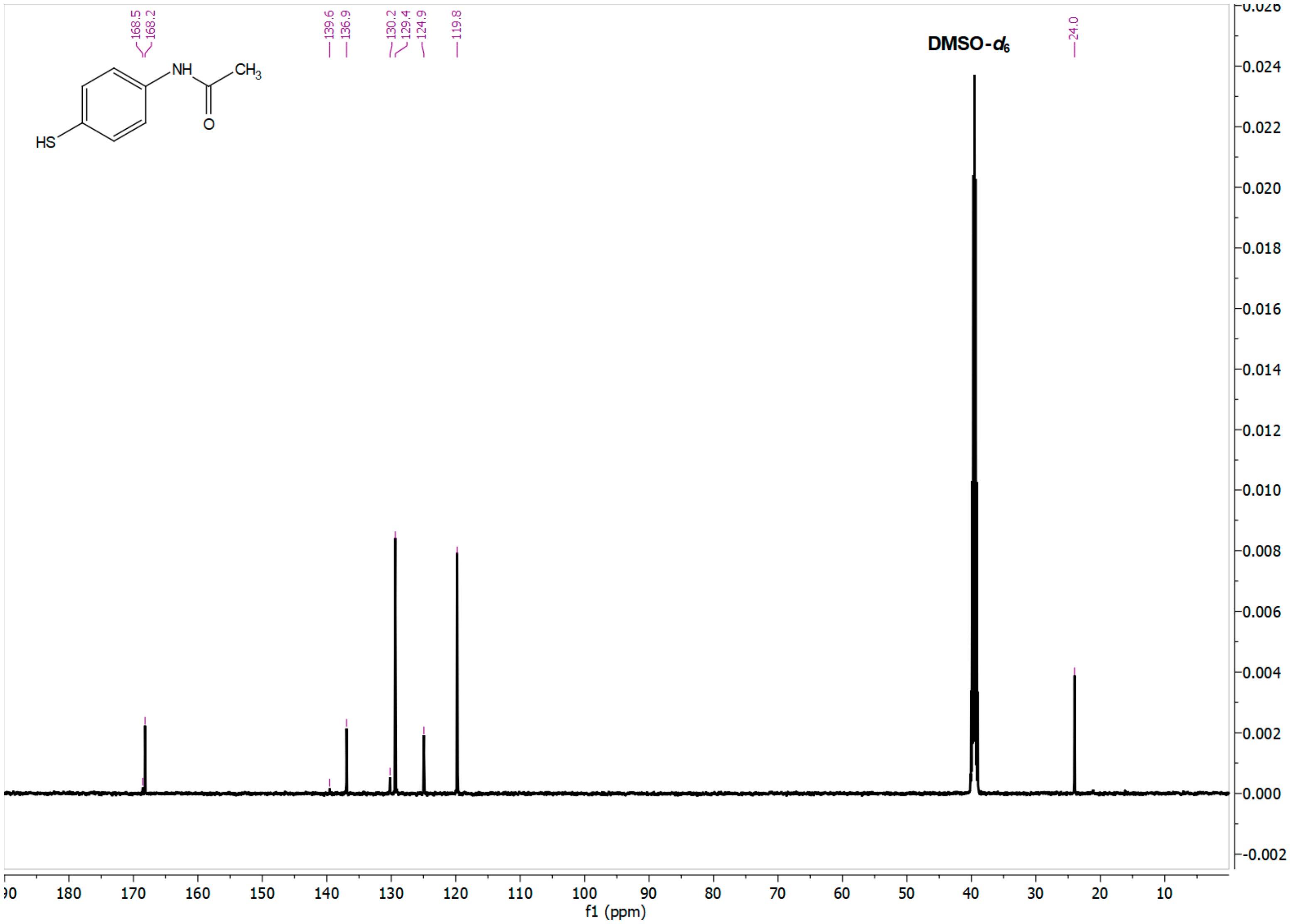

Figure S6. $^{13}C\{^1H\}$ NMR spectrum (126 MHz) of compound **3** in DMSO-*d*6.

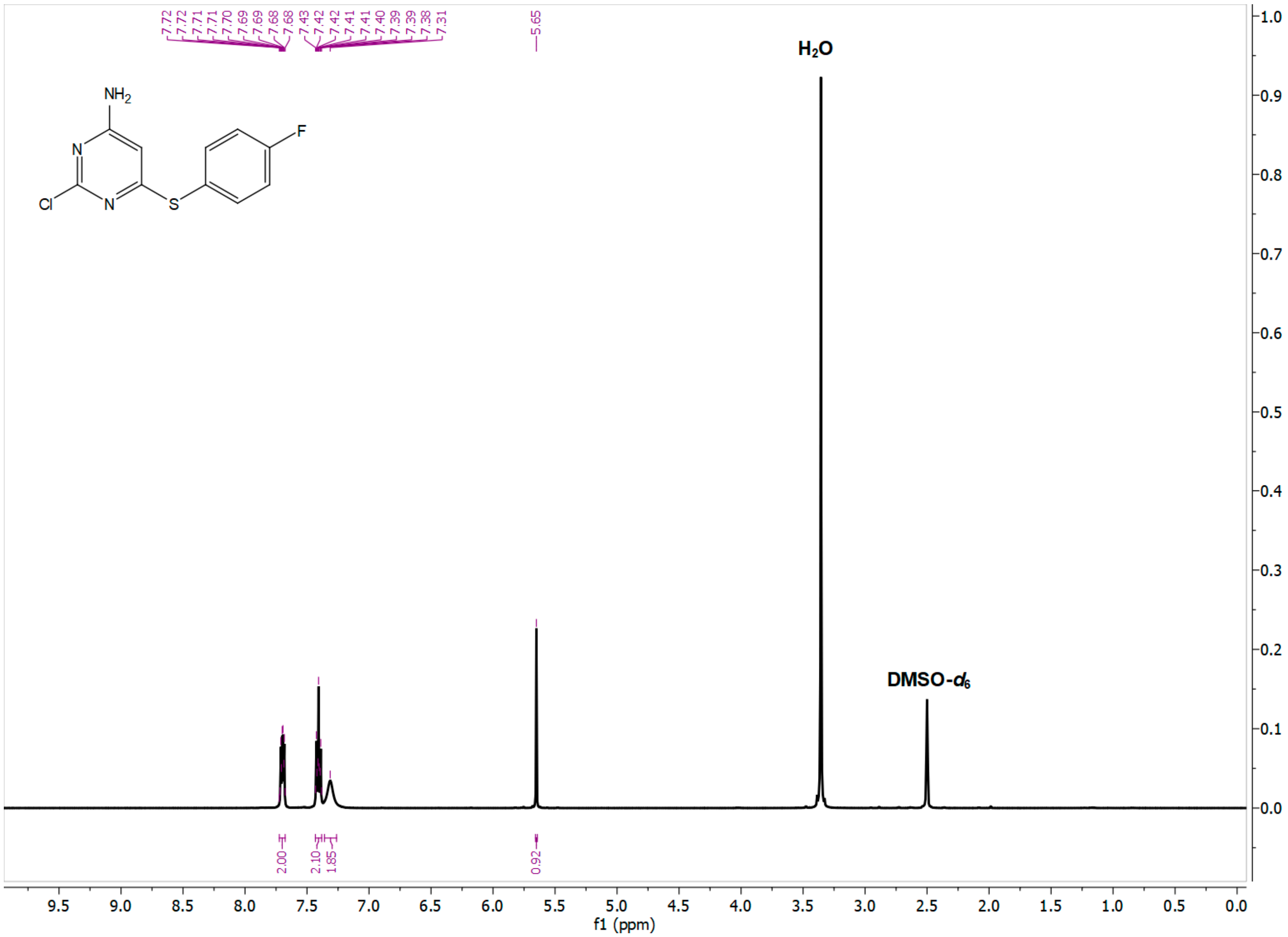

Figure S7. ^{1}H NMR spectrum (500 MHz) of compound **C1** in DMSO-*d*6.

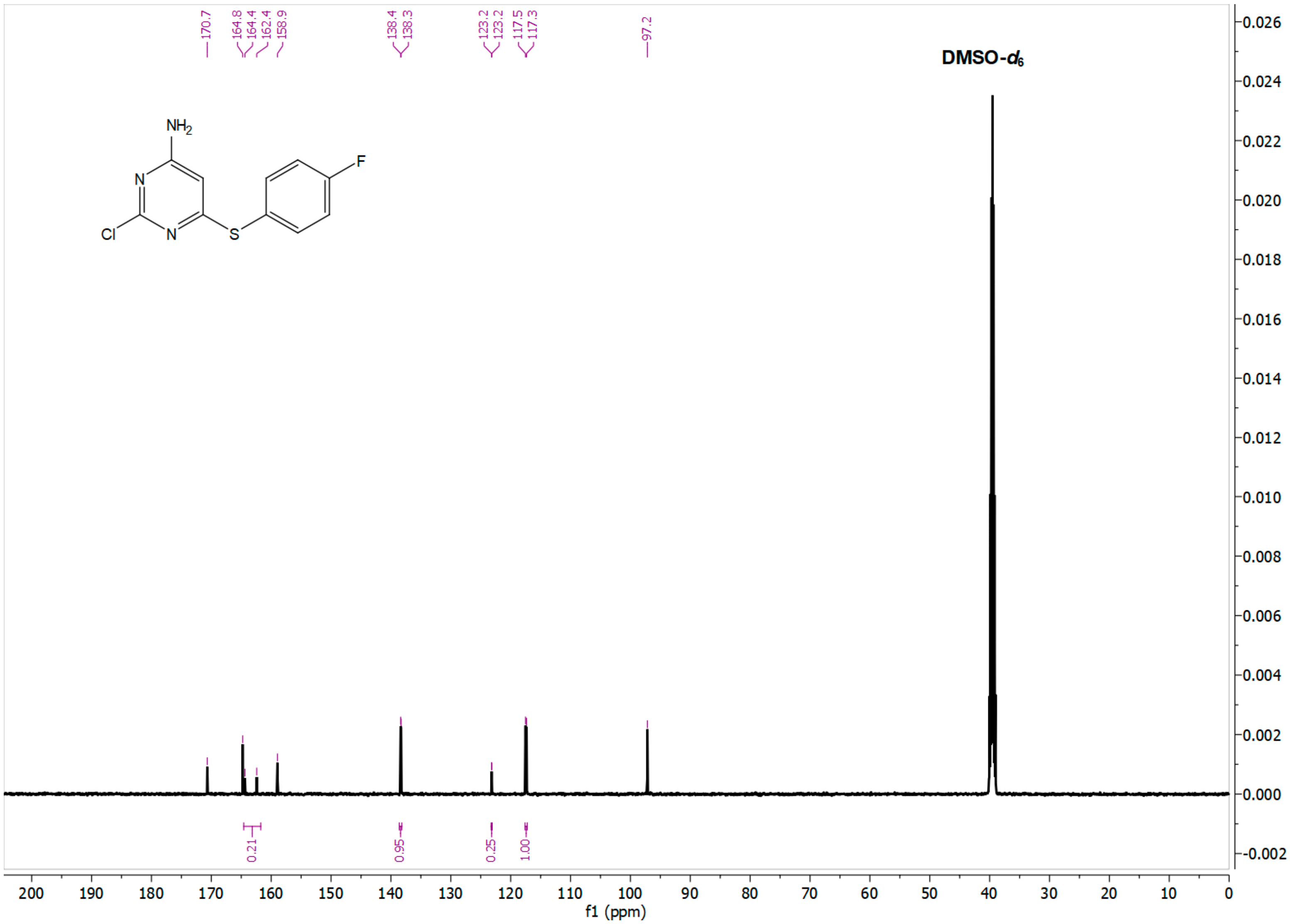

Figure S8. $^{13}C\{^{1}H\}$ NMR spectrum (126 MHz) of compound **C1** in DMSO-d_6.

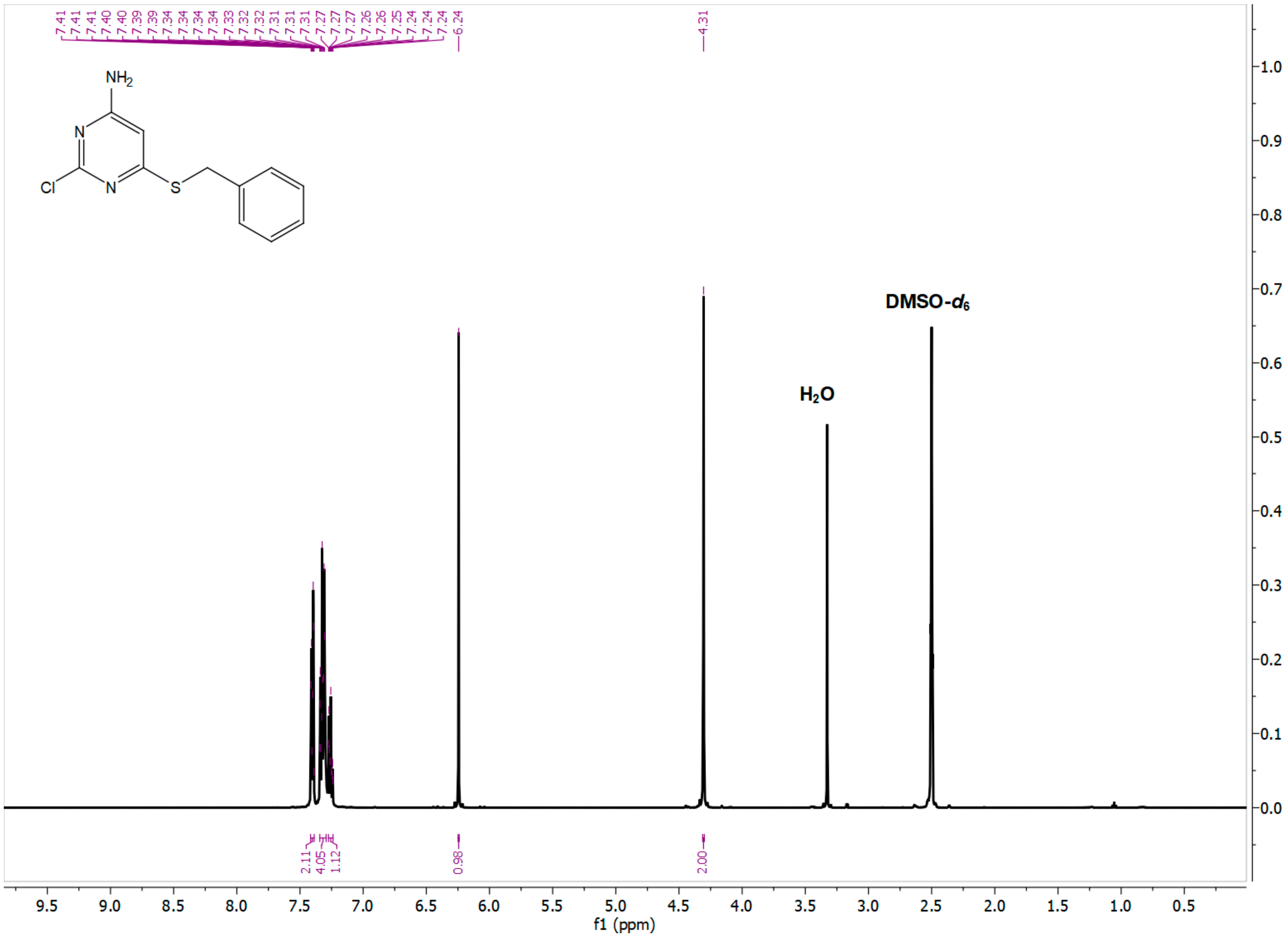

Figure S9. ^{1}H NMR spectrum (500 MHz) of compound **C2** in DMSO-*d*6.

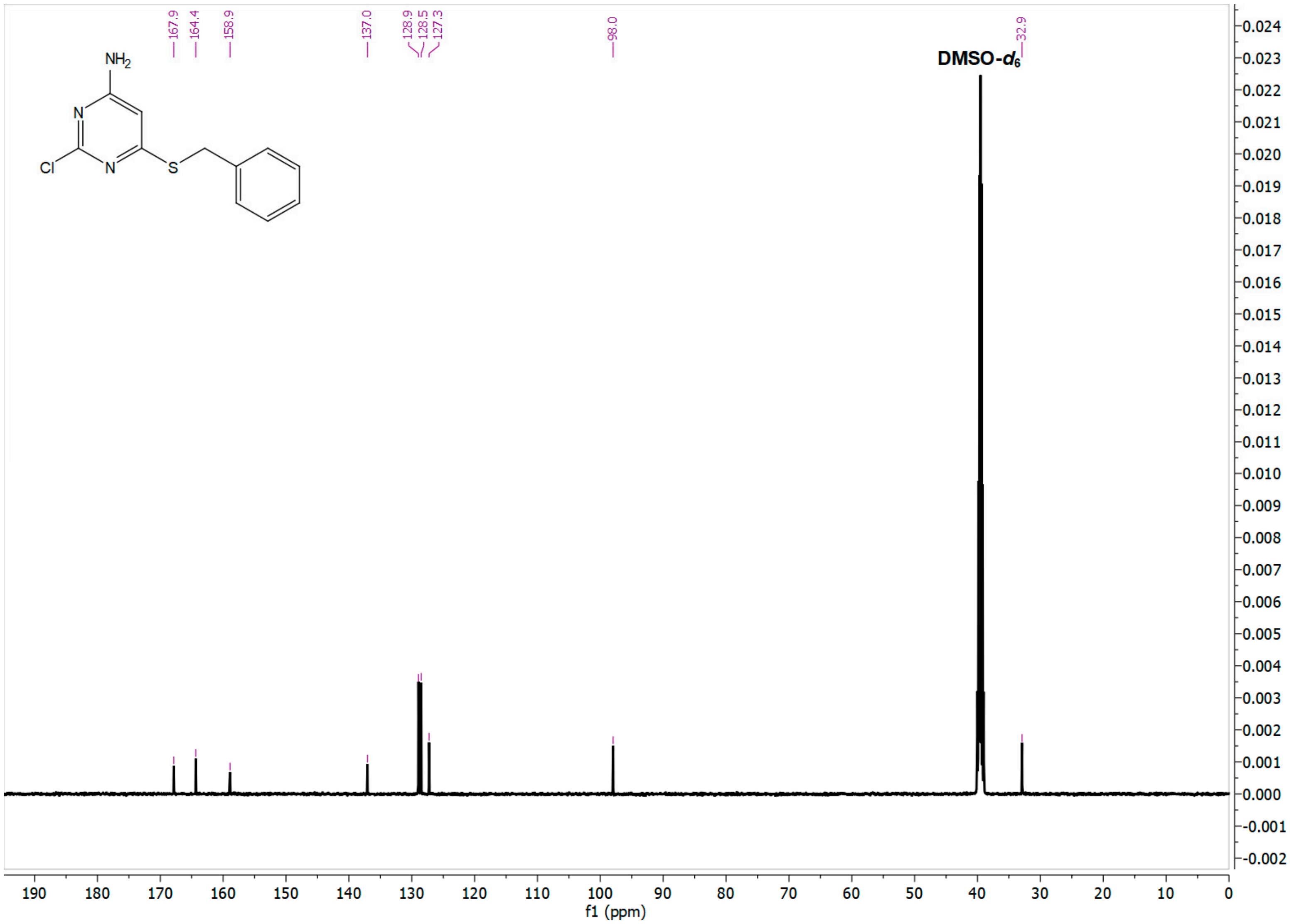

Figure S10. $^{13}C\{^1H\}$ NMR spectrum (126 MHz) of compound **C2** in DMSO-*d*6.

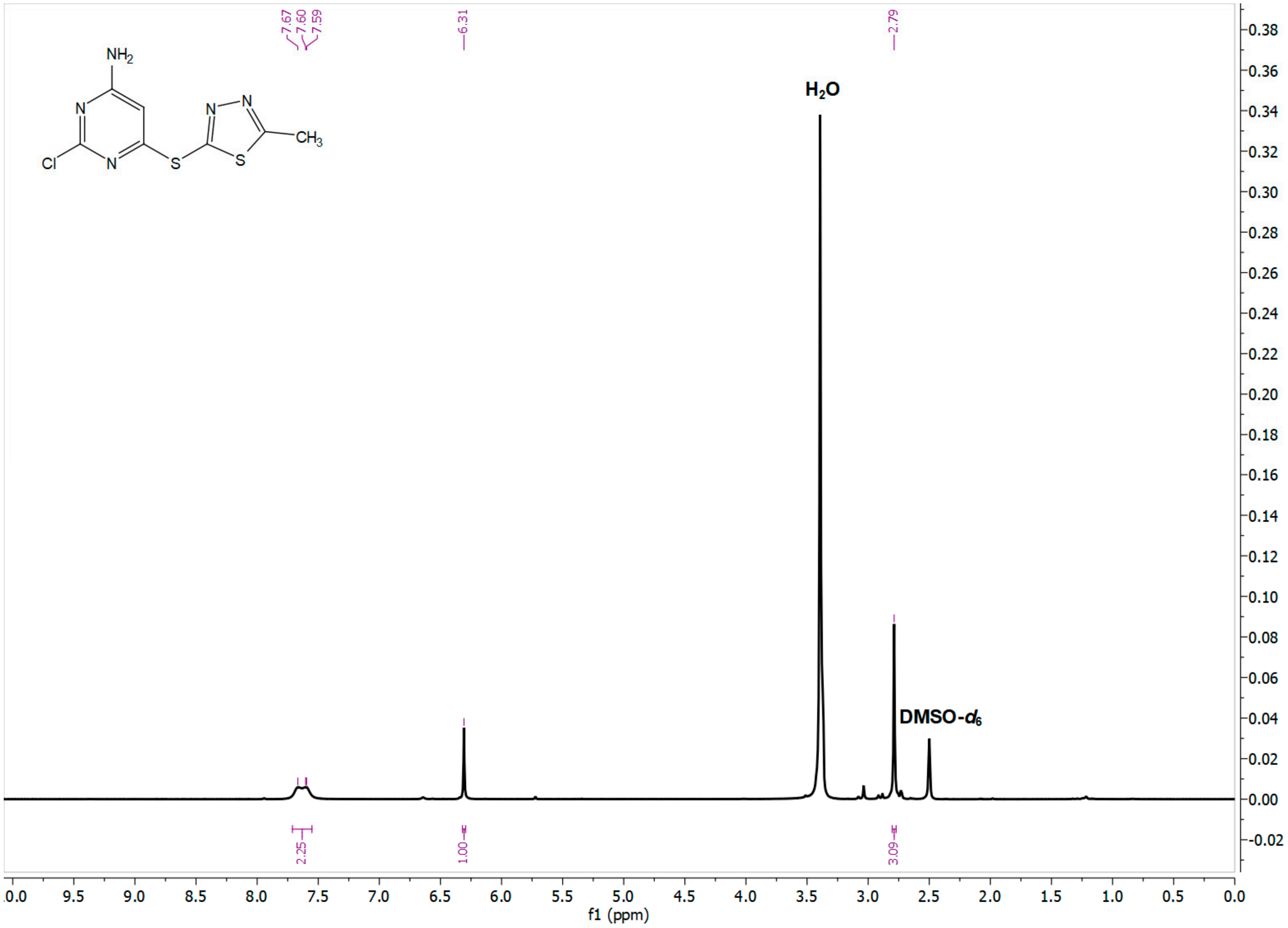

Figure S11. ^{1}H NMR spectrum (500 MHz) of compound **C3** in DMSO-d_6.

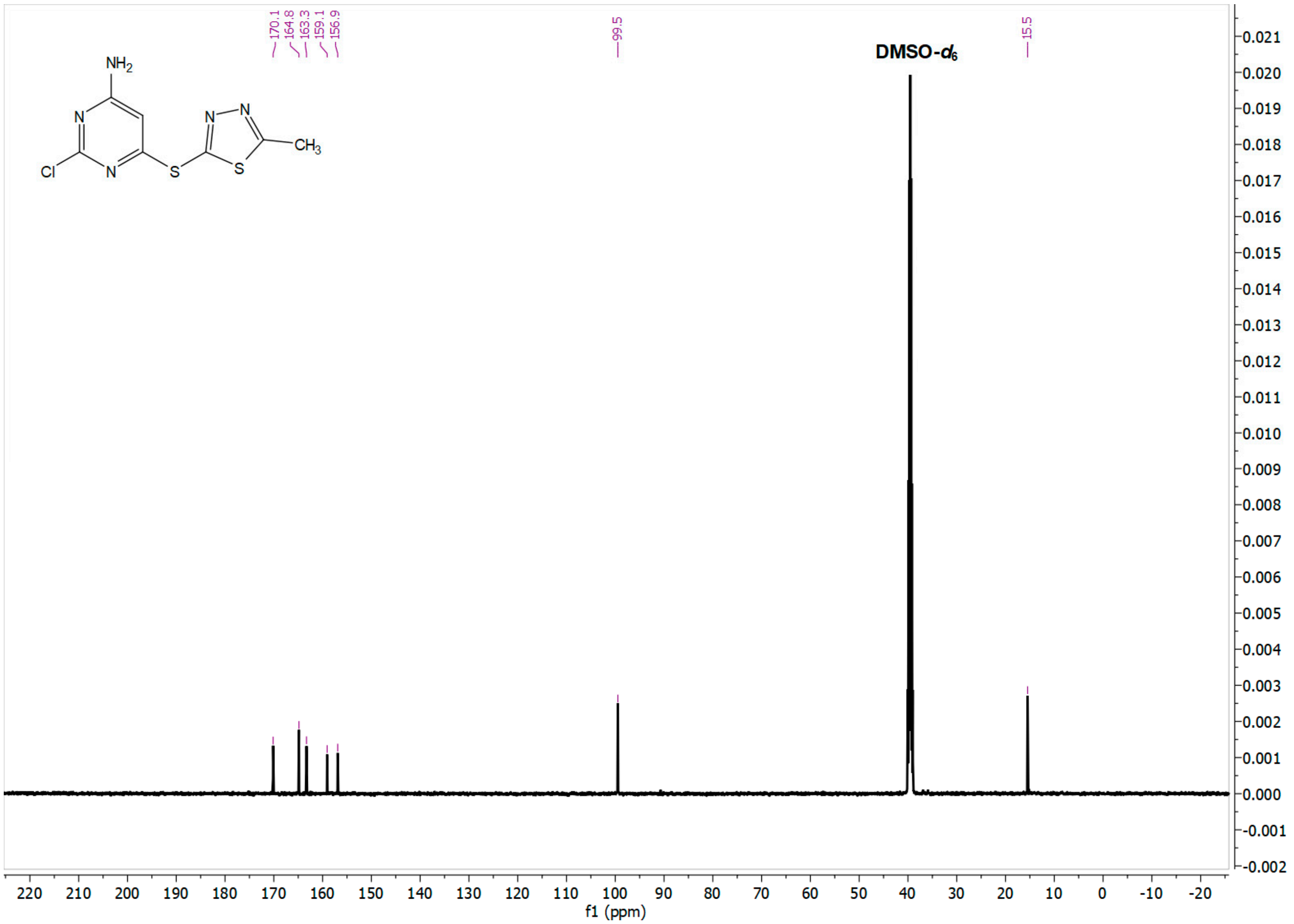

Figure S12. $^{13}C\{^1H\}$ NMR spectrum (126 MHz) of compound **C3** in DMSO-d_6.

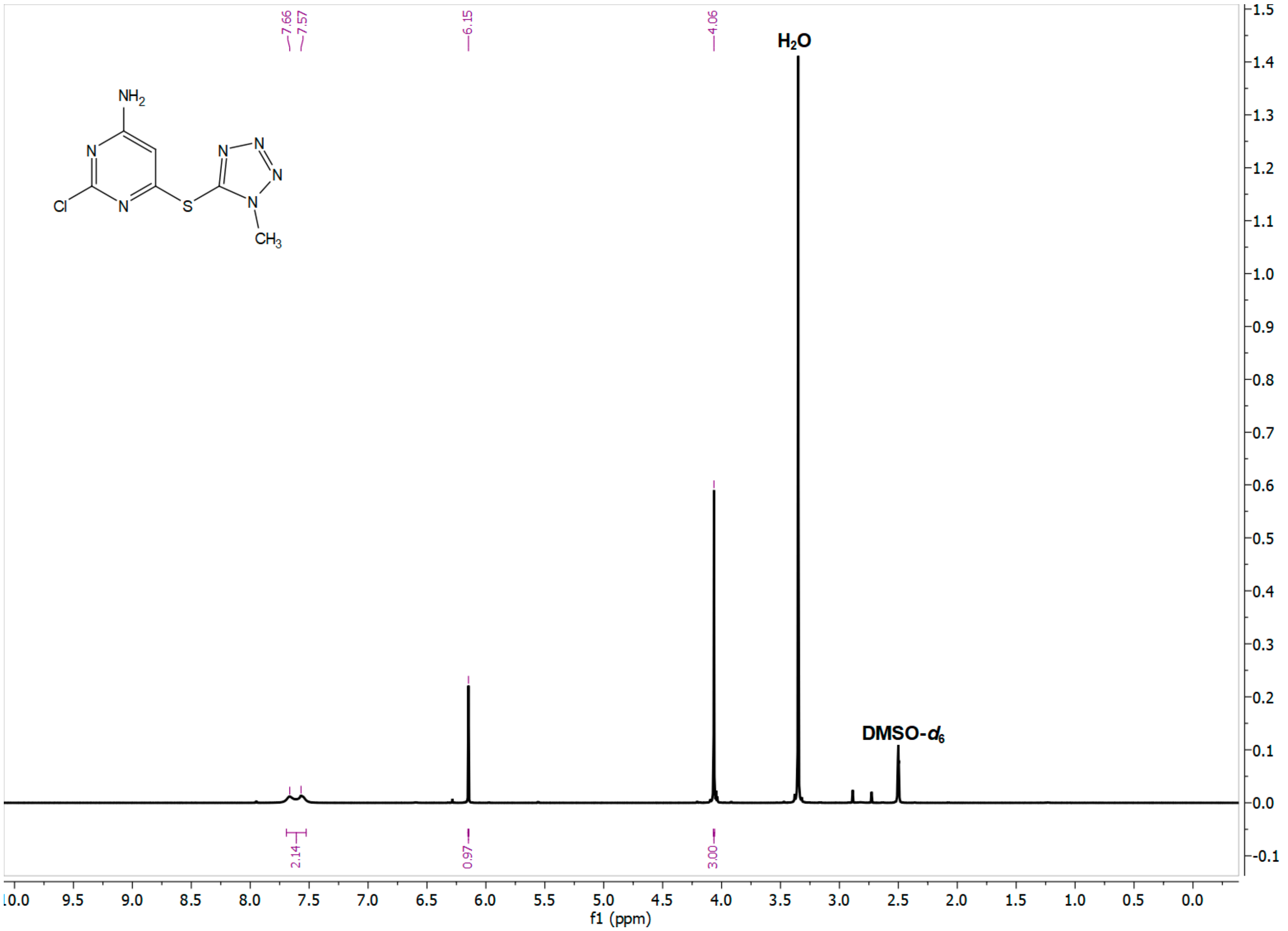

Figure S13. ^{1}H NMR spectrum (500 MHz) of compound **C4** in DMSO-*d*6.

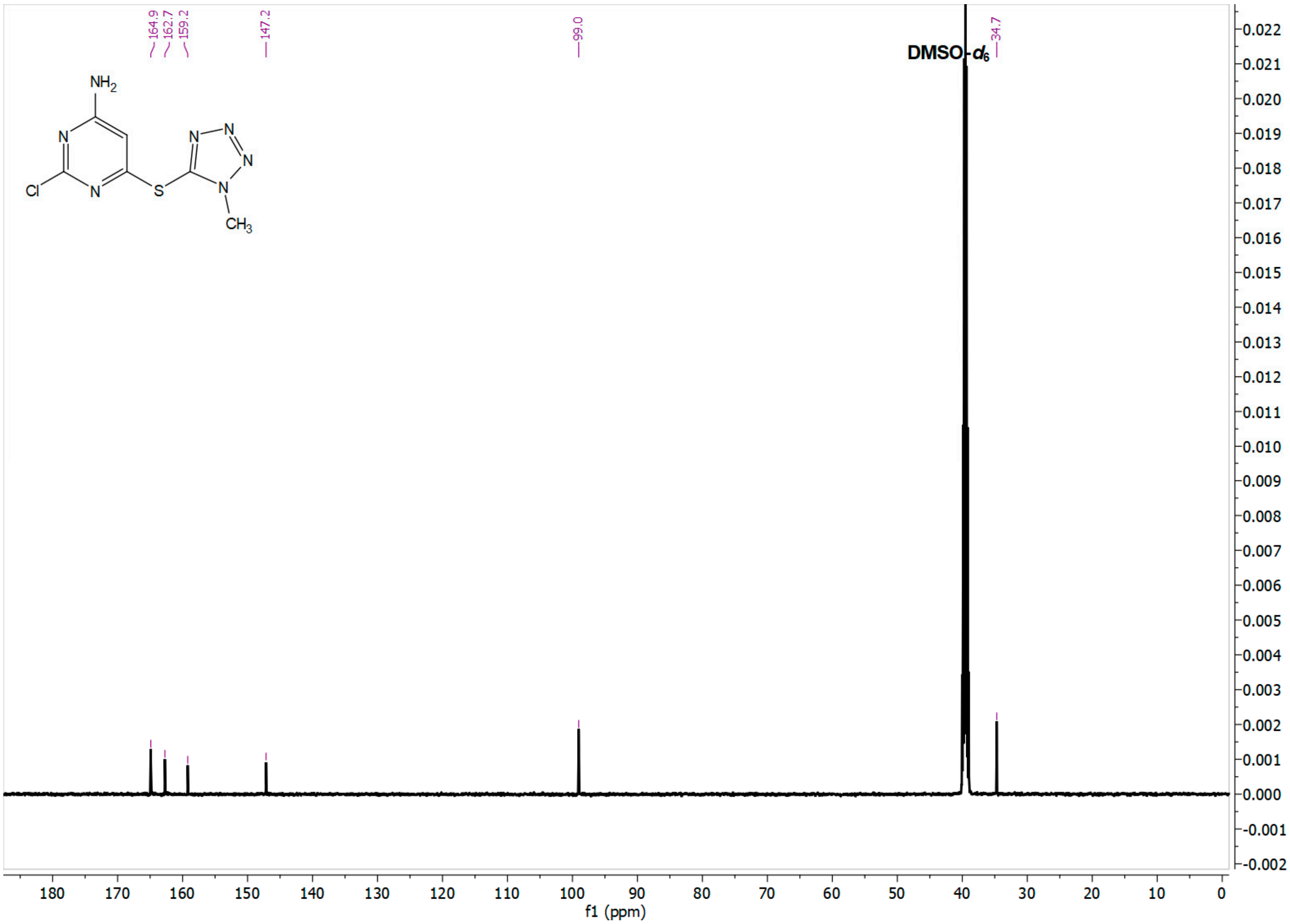

Figure S14. $^{13}C\{^1H\}$ NMR spectrum (126 MHz) of compound **C4** in DMSO-d_6.

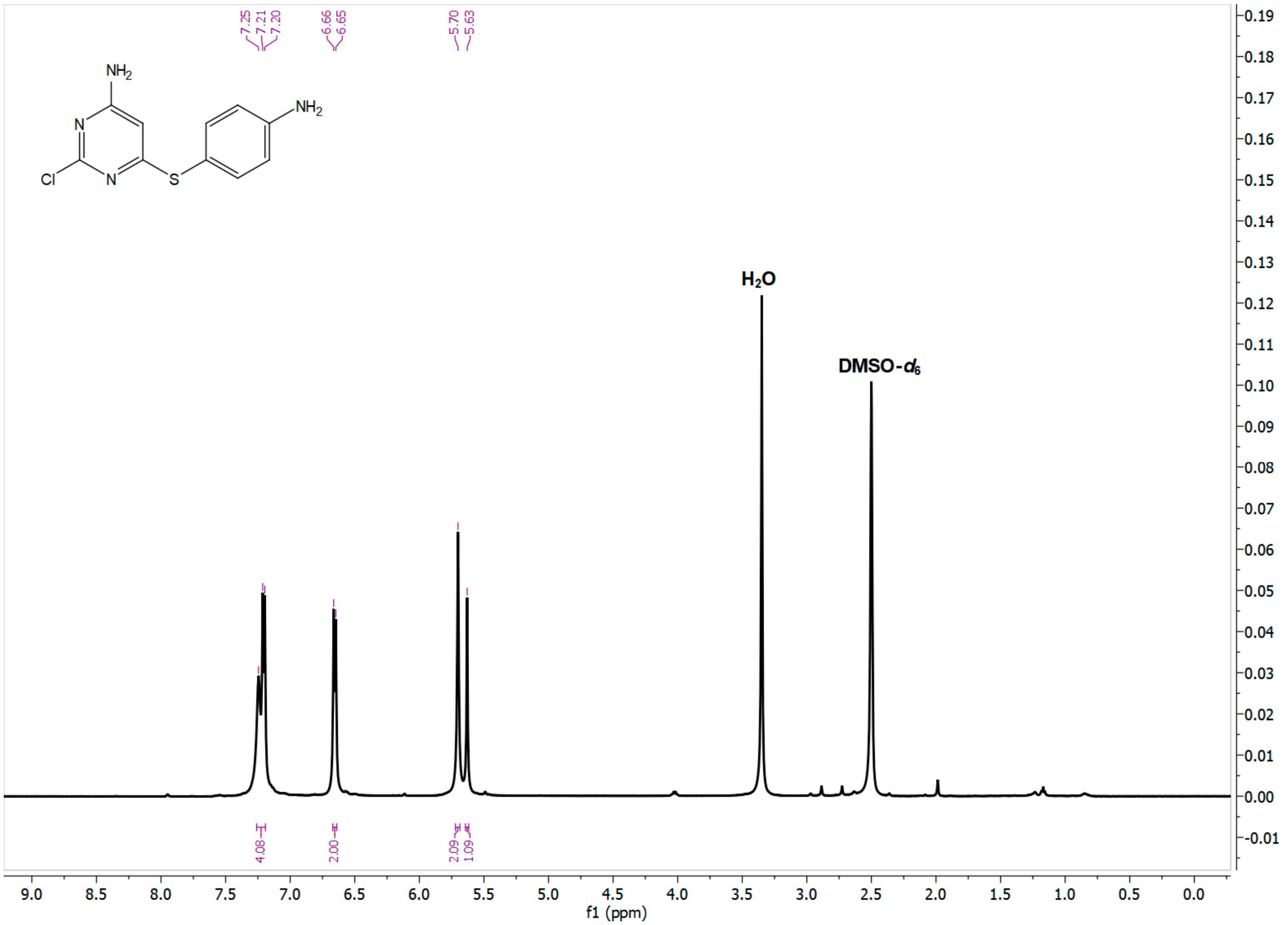

Figure S15. ^{1}H NMR spectrum (500 MHz) of compound **C5** in DMSO-d_6.

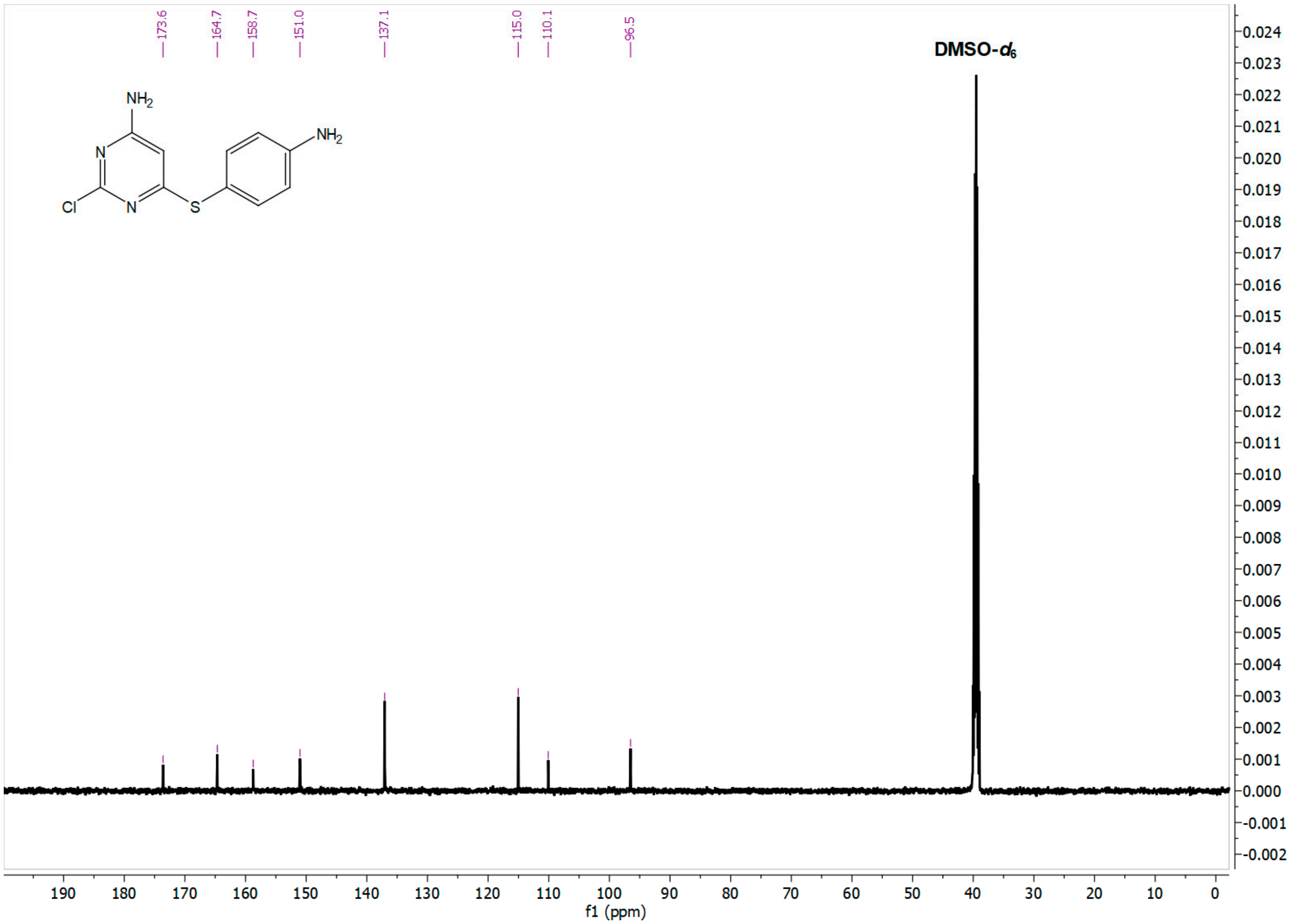

Figure S16. $^{13}C\{^1H\}$ NMR spectrum (126 MHz) of compound **C5** in DMSO-d_6.

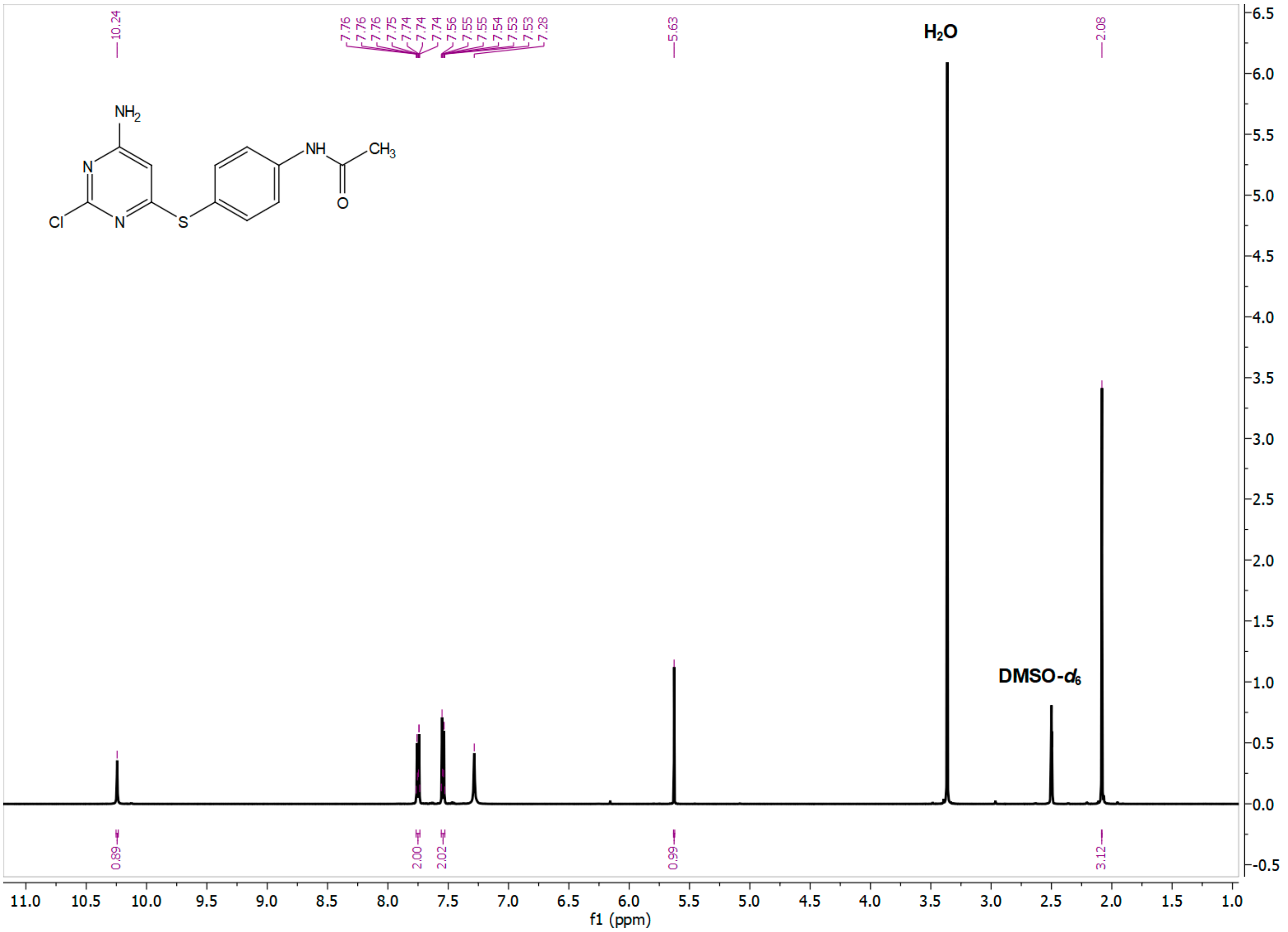

Figure S17. ^{1}H NMR spectrum (500 MHz) of compound **C6** in DMSO-d_6.

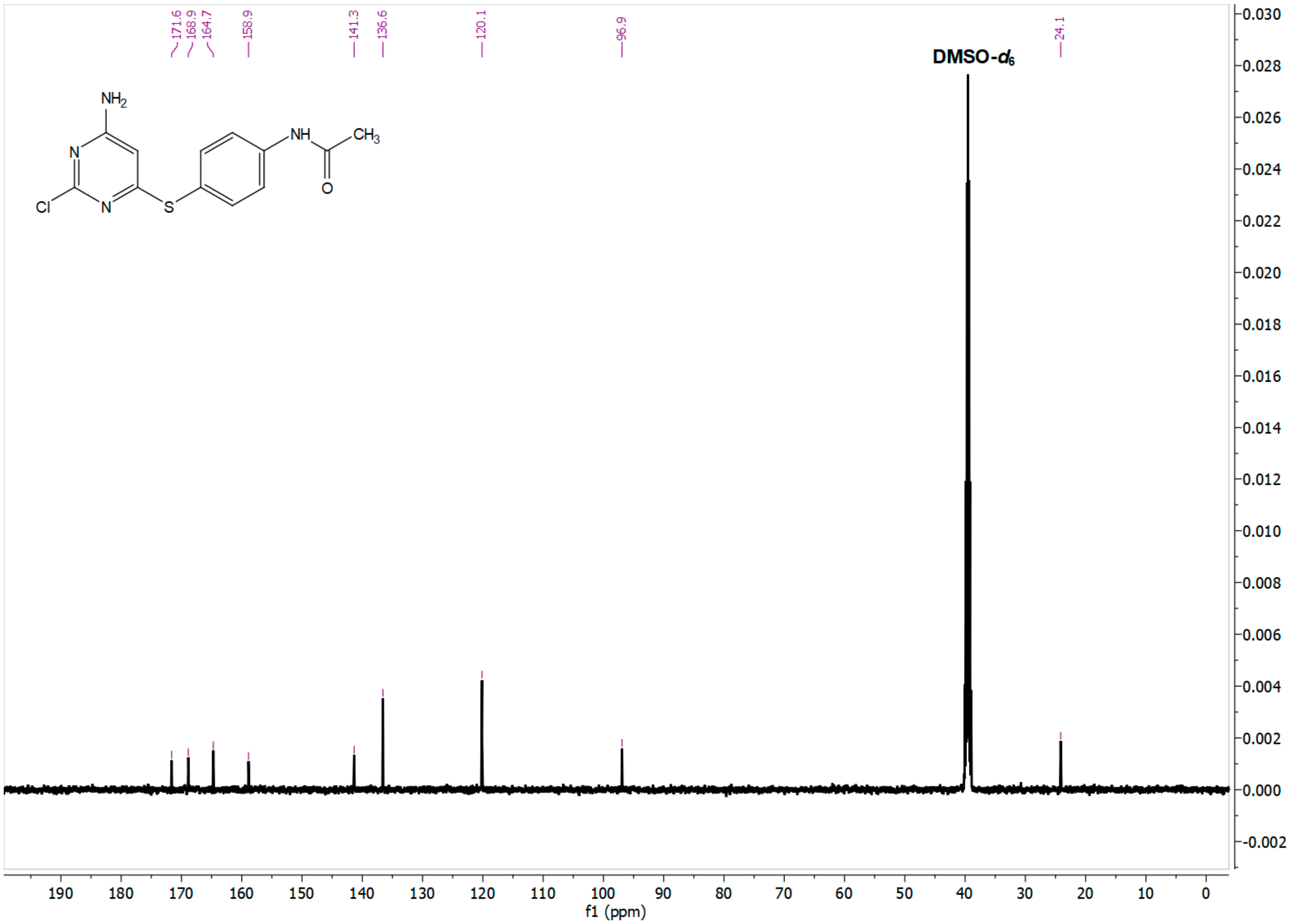

Figure S18. $^{13}C\{^1H\}$ NMR spectrum (126 MHz) of compound **C6** in DMSO-d_6.

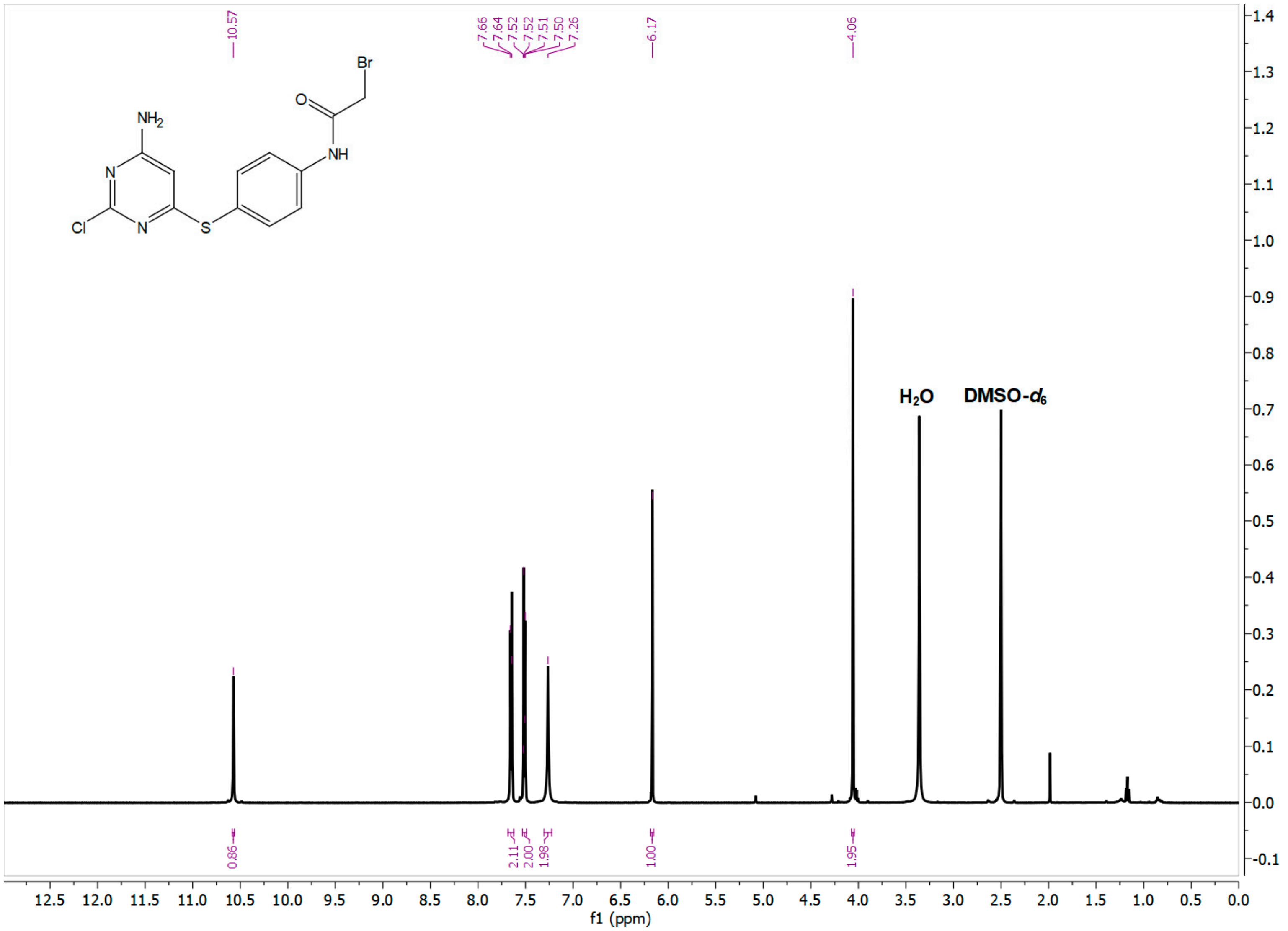

Figure S19. ^{1}H NMR spectrum (500 MHz) of compound **C7** in DMSO-d_6.

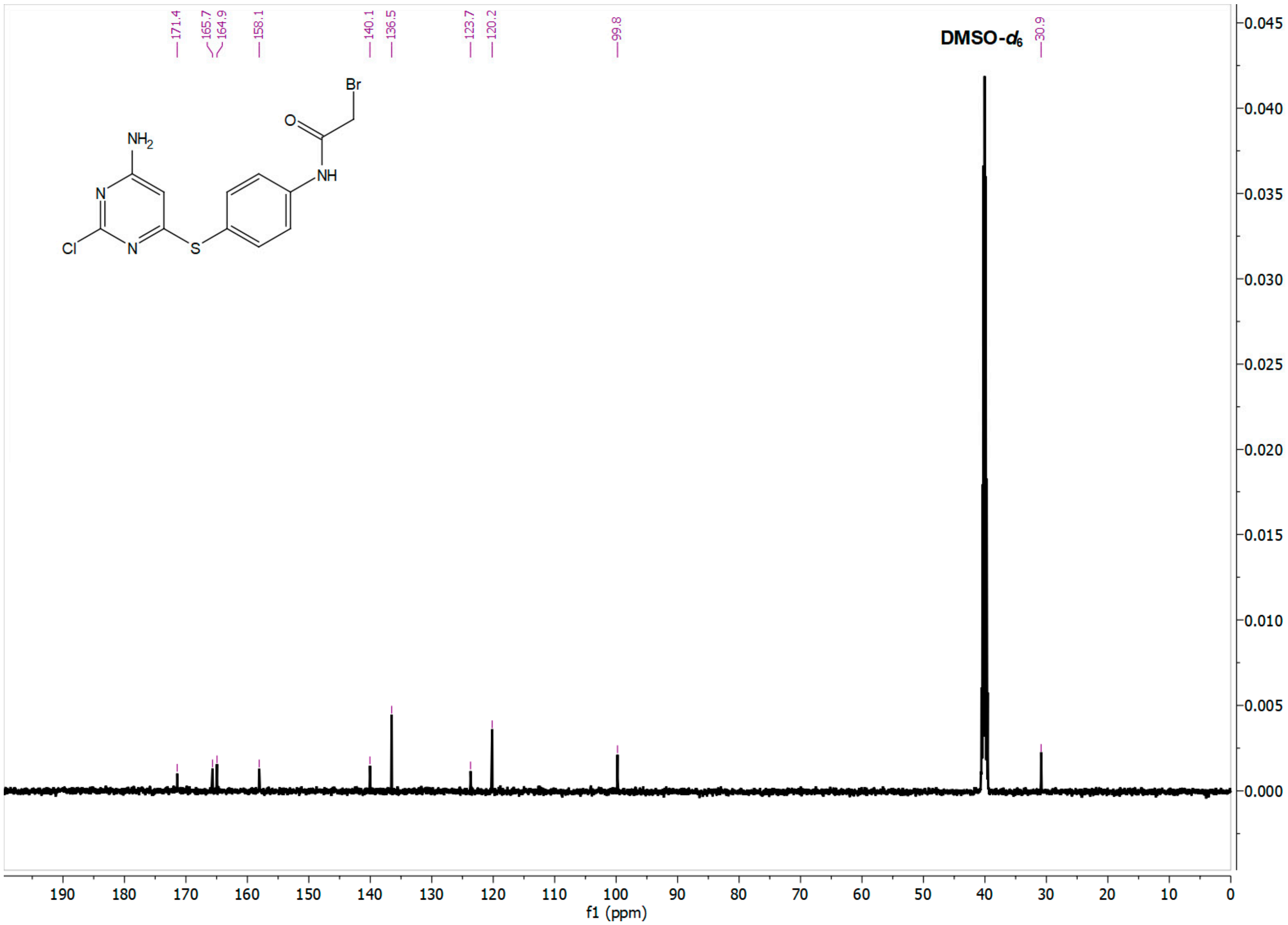

Figure S20. $^{13}C\{^1H\}$ NMR spectrum (126 MHz) of compound **C7** in DMSO-*d*$_6$.

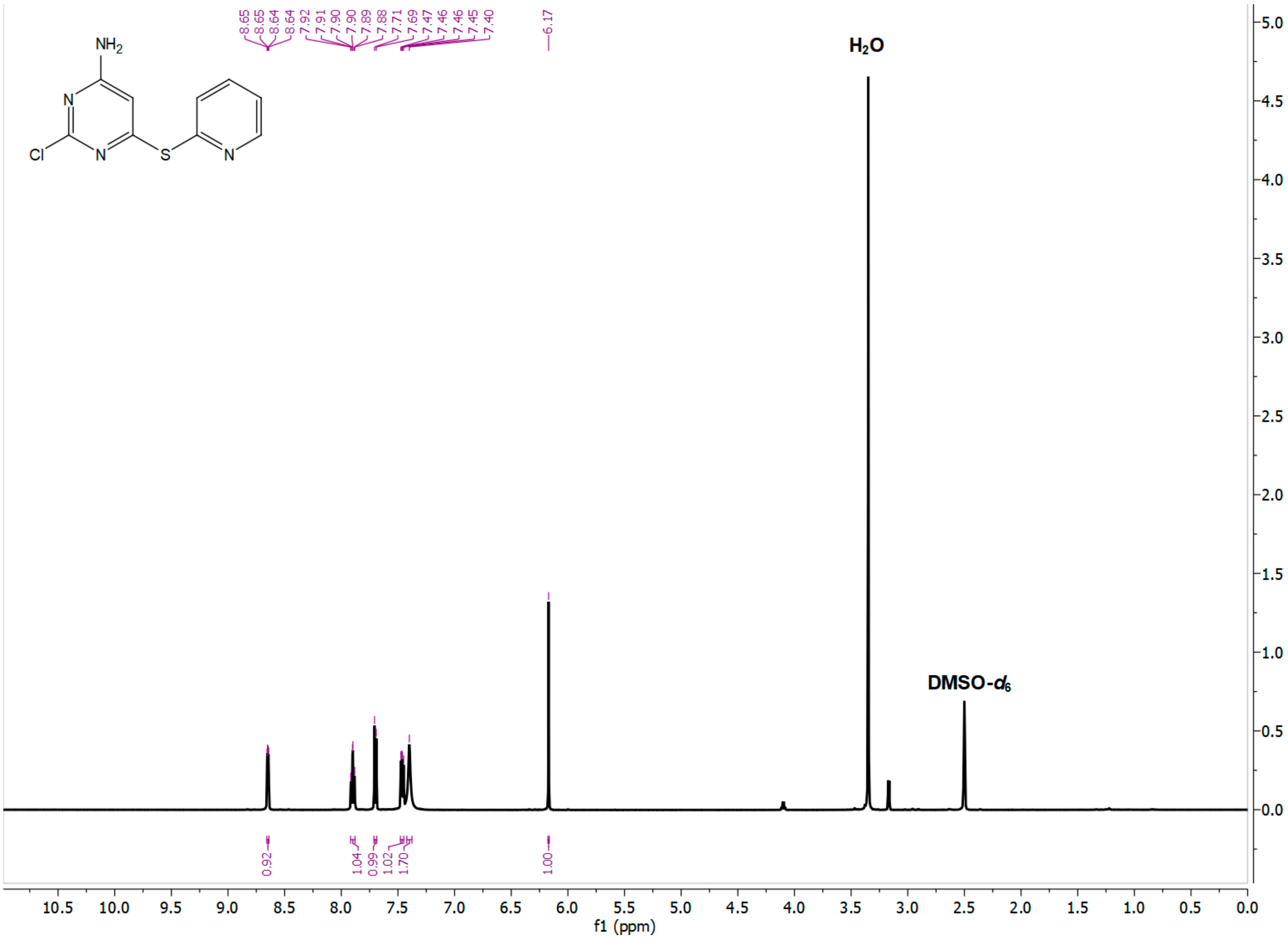

Figure S21. ^{1}H NMR spectrum (500 MHz) of compound **C8** in DMSO-d_6.

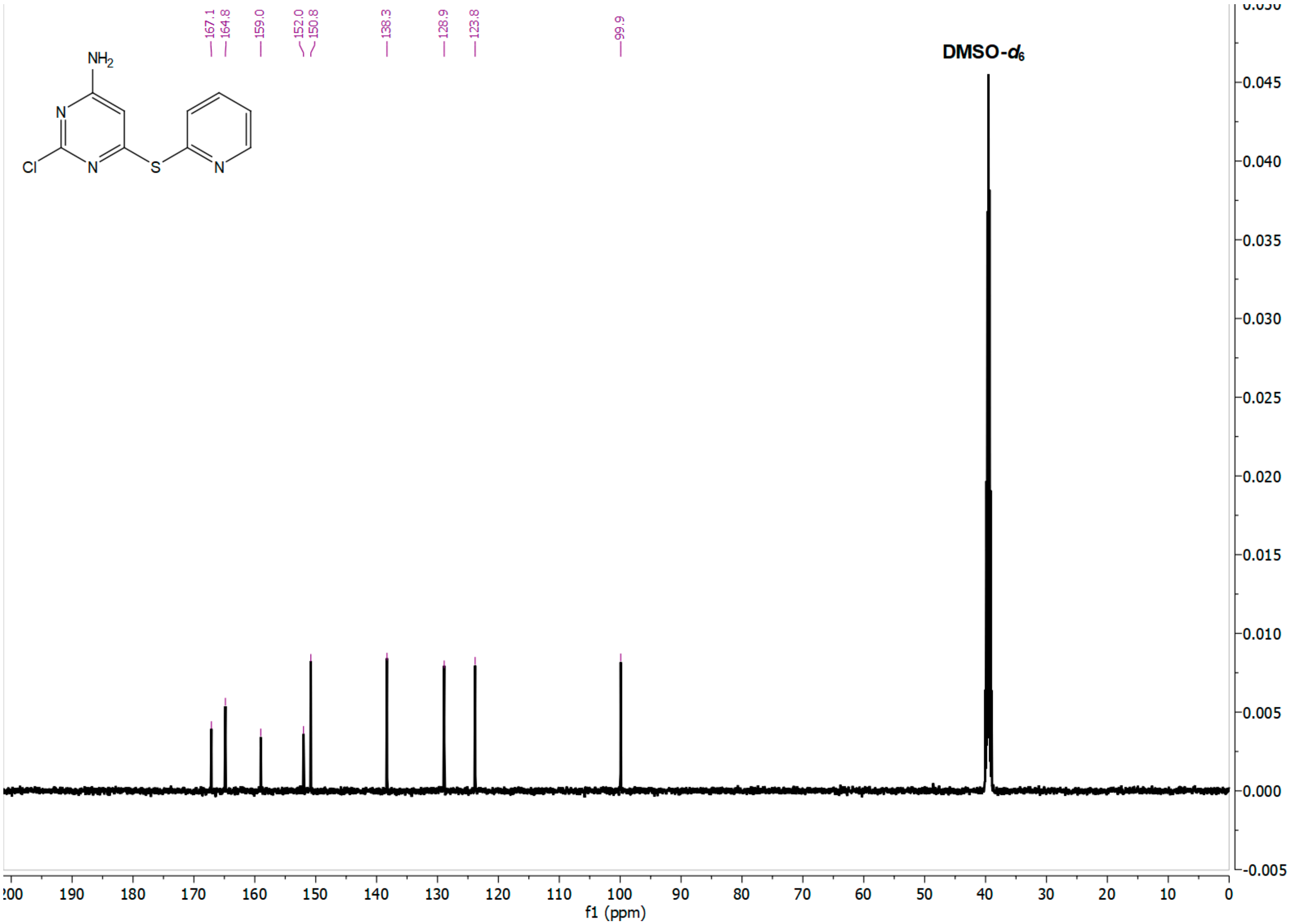

Figure S22. $^{13}C\{^{1}H\}$ NMR spectrum (126 MHz) of compound **C8** in DMSO-*d₆*.

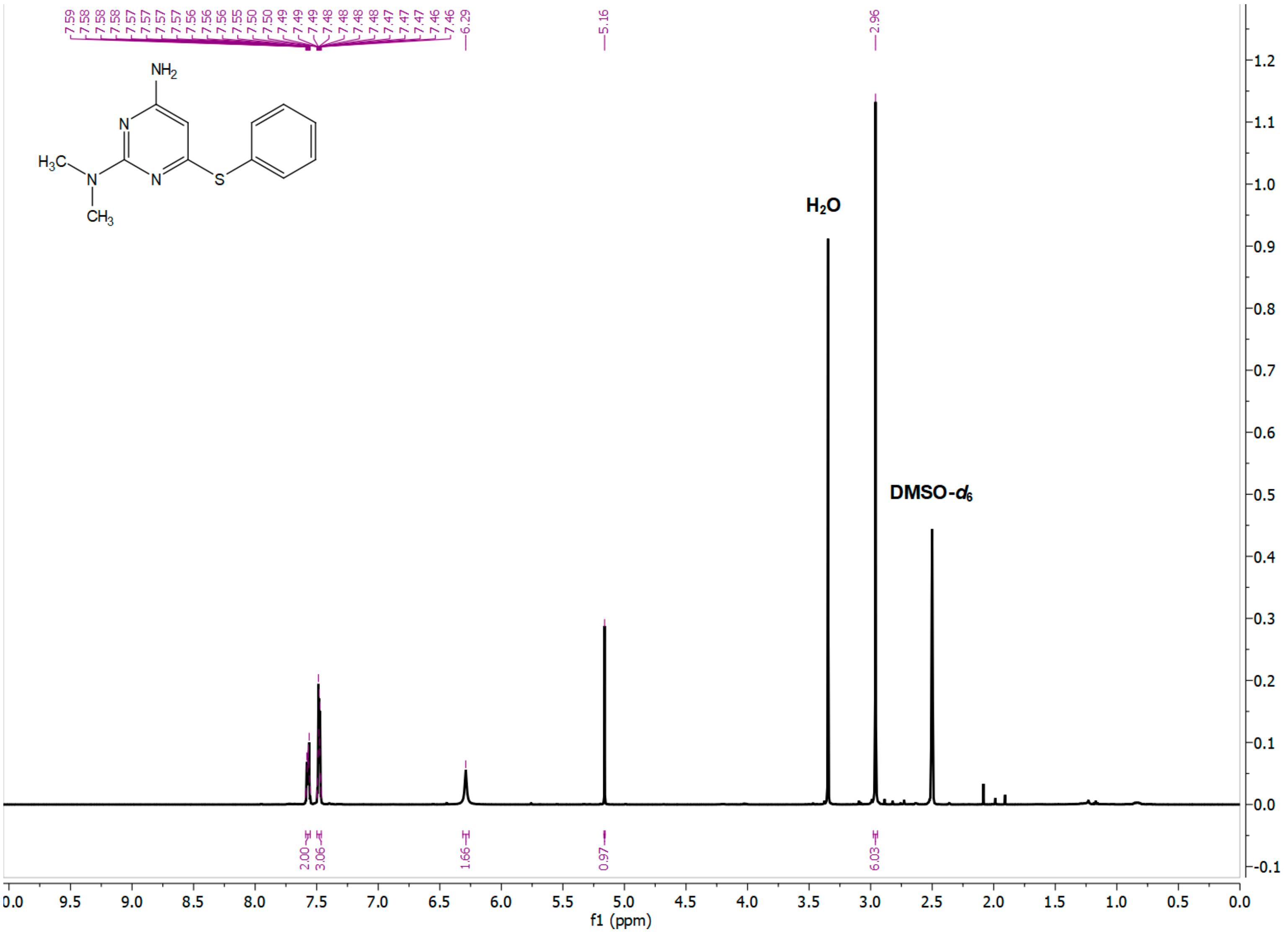

Figure S23. ^{1}H NMR spectrum (500 MHz) of compound **C9** in DMSO-d_6.

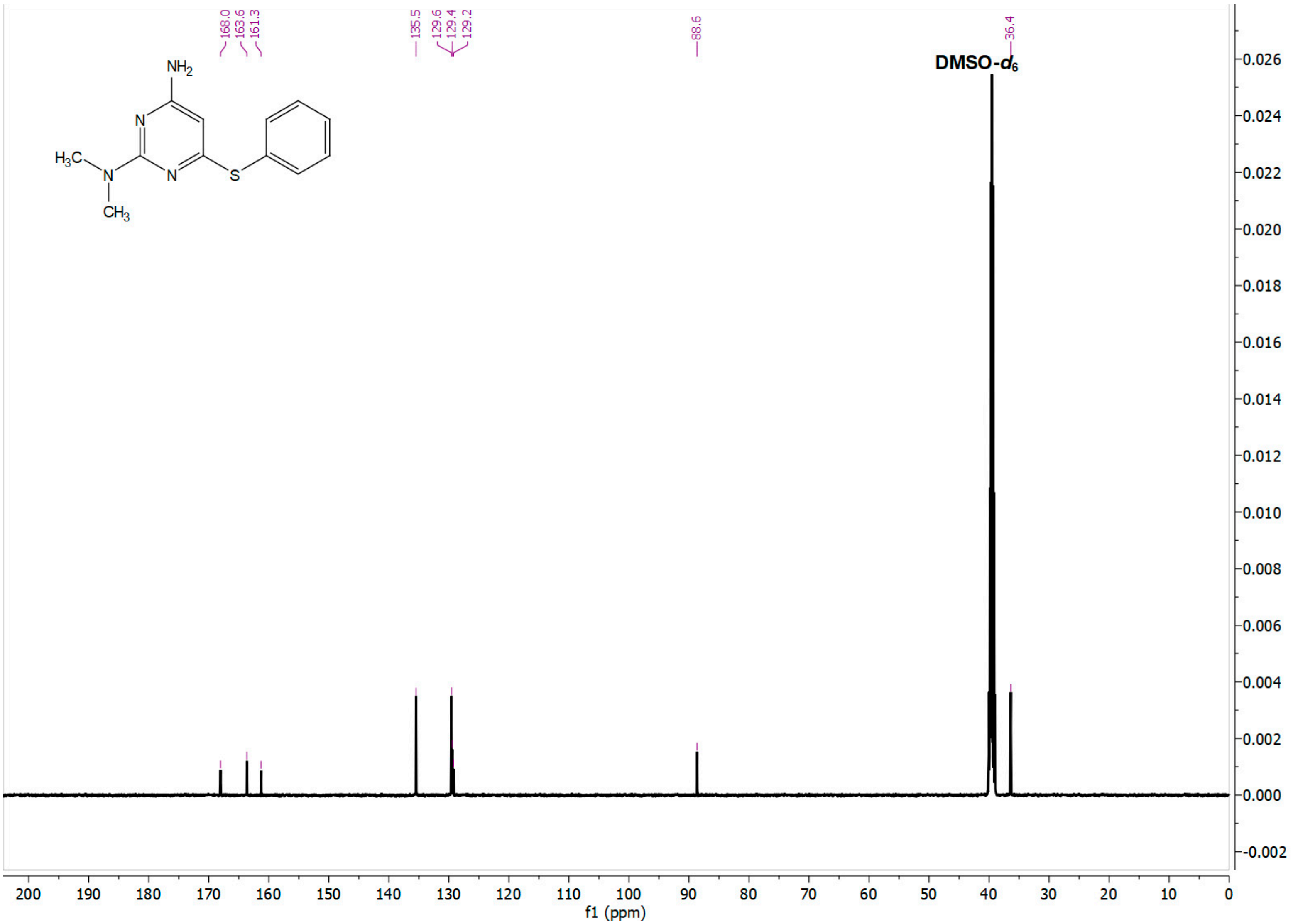

Figure S24. $^{13}C\{^1H\}$ NMR spectrum (126 MHz) of compound **C9** in DMSO-d_6.

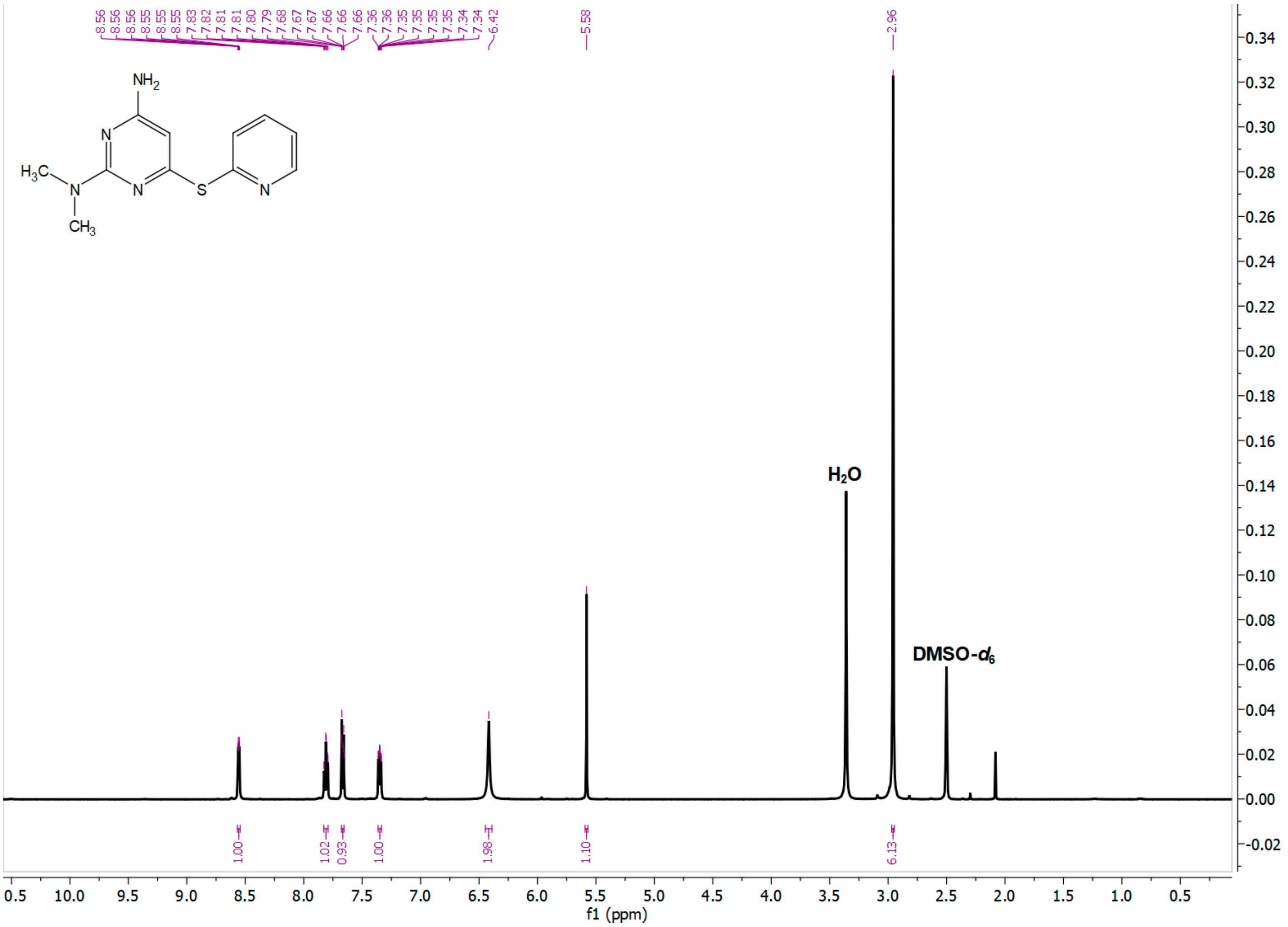

Figure S25. ^{1}H NMR spectrum (500 MHz) of compound **C10** in DMSO-*d*6.

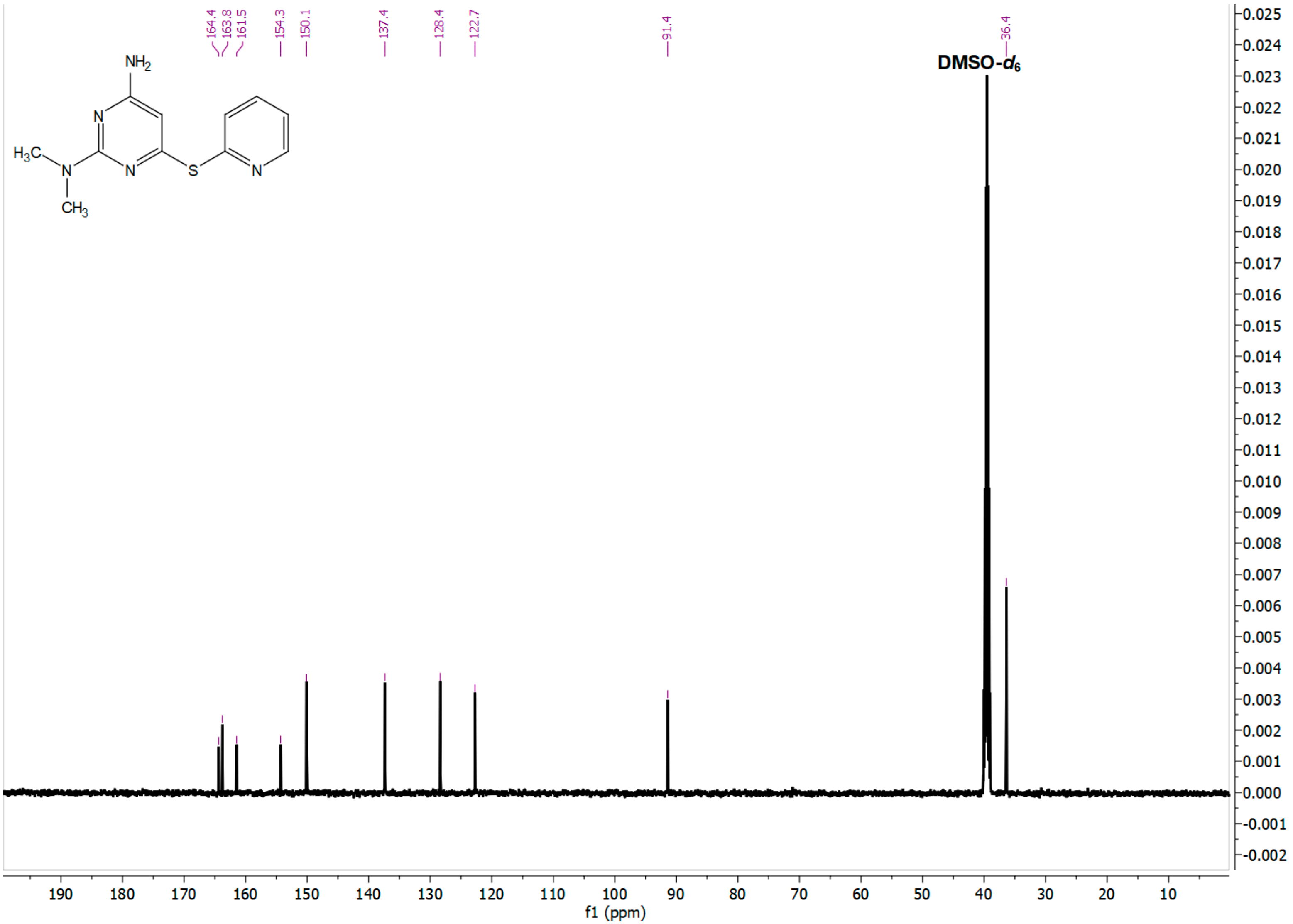

Figure S26. $^{13}C\{^1H\}$ NMR spectrum (126 MHz) of compound **C10** in DMSO-d_6.

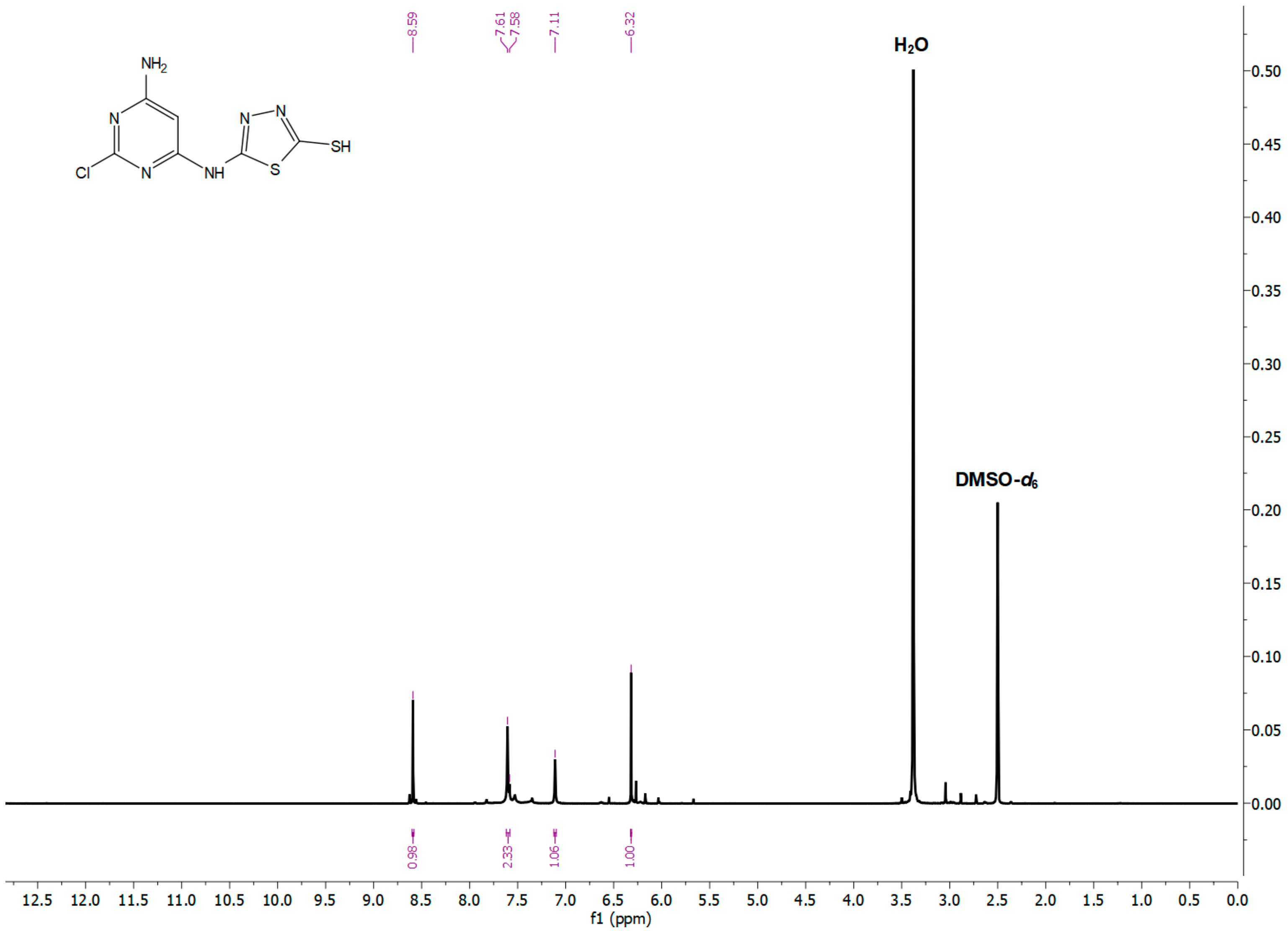

Figure S27. ^{1}H NMR spectrum (500 MHz) of compound **C12** in DMSO-d_6.

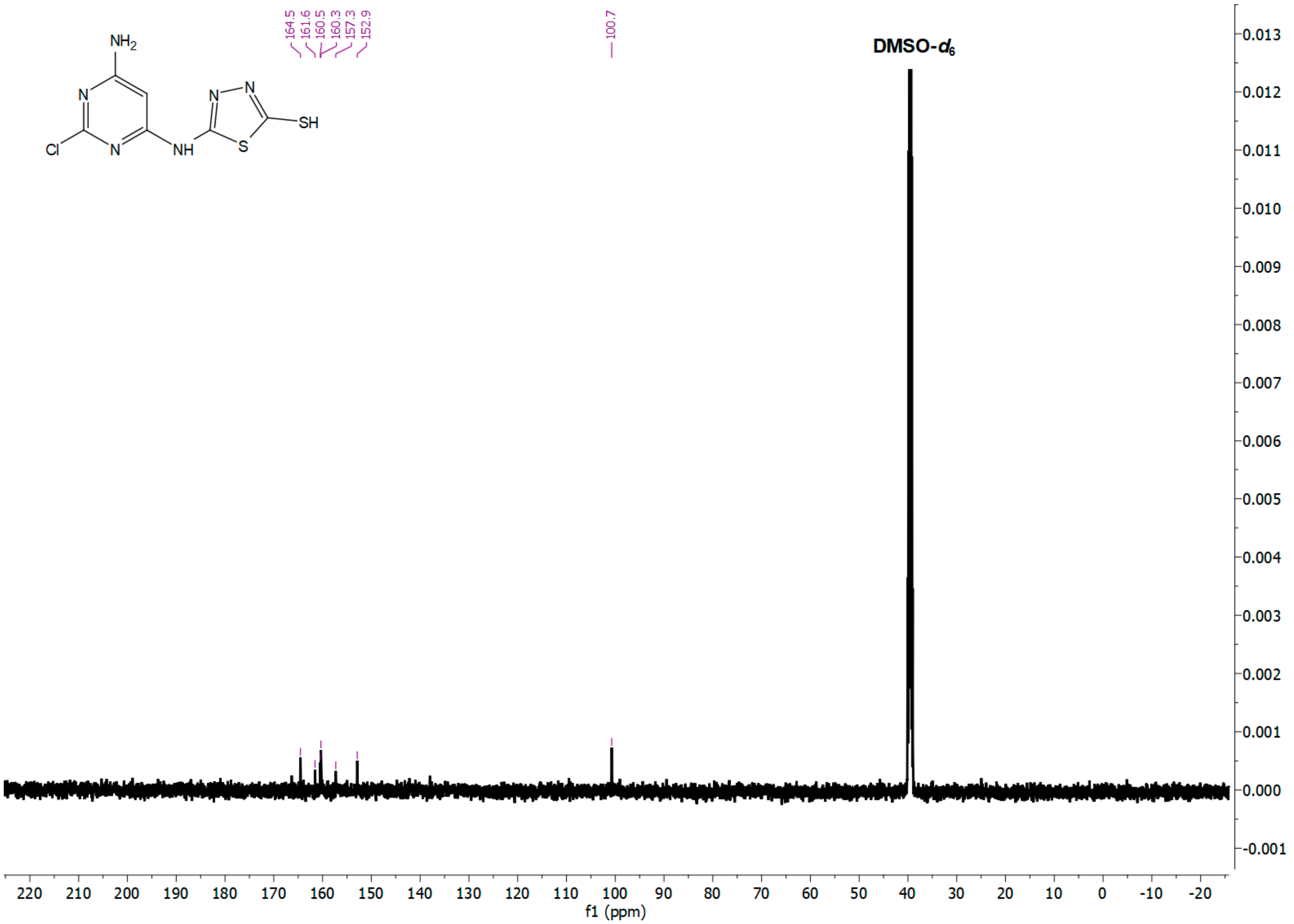

Figure S28. $^{13}C\{^{1}H\}$ NMR spectrum (126 MHz) of compound **C12** in DMSO-d_6.

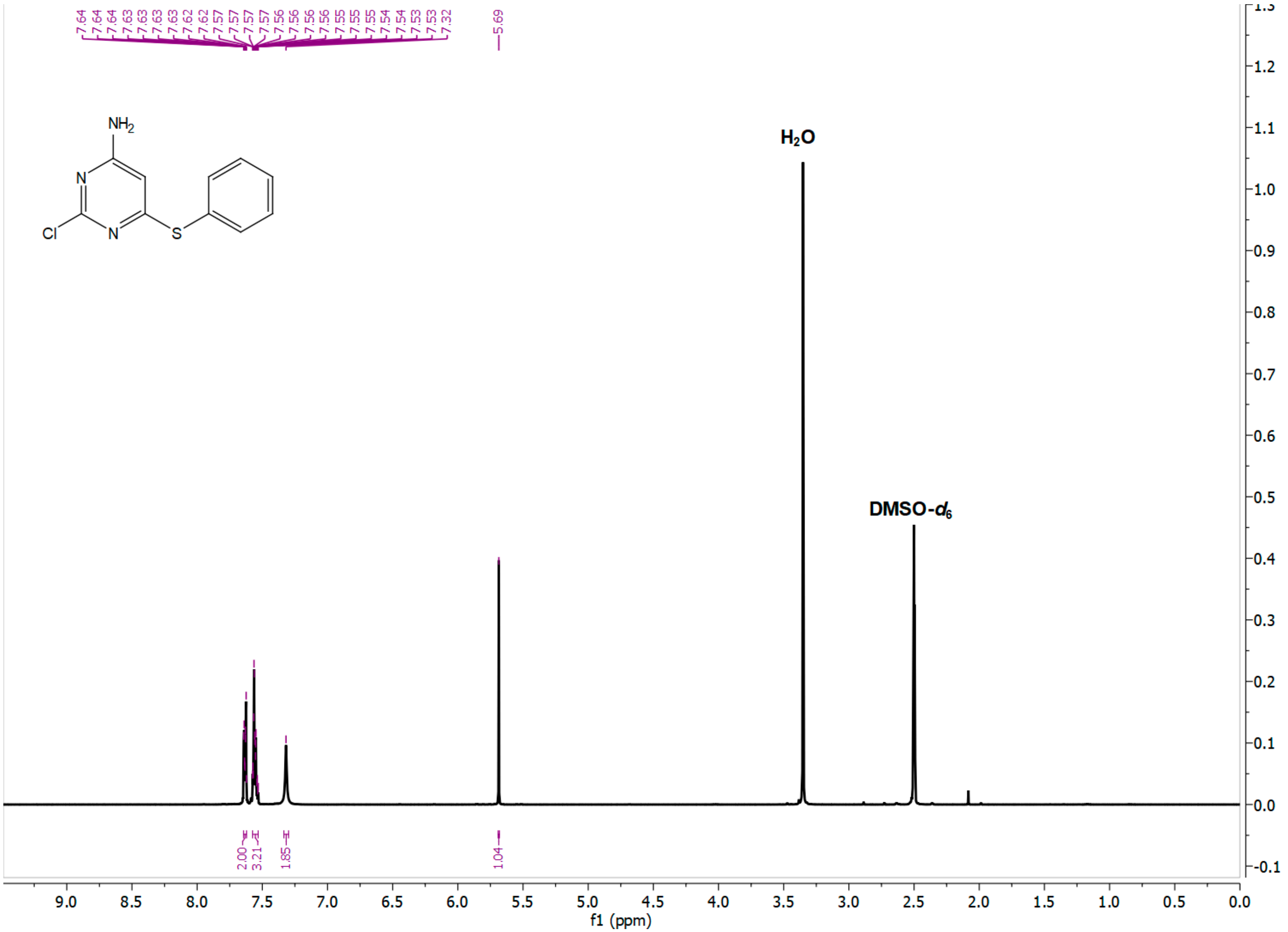

Figure S29. ^{1}H NMR spectrum (500 MHz) of compound **C13** in DMSO-*d*6.

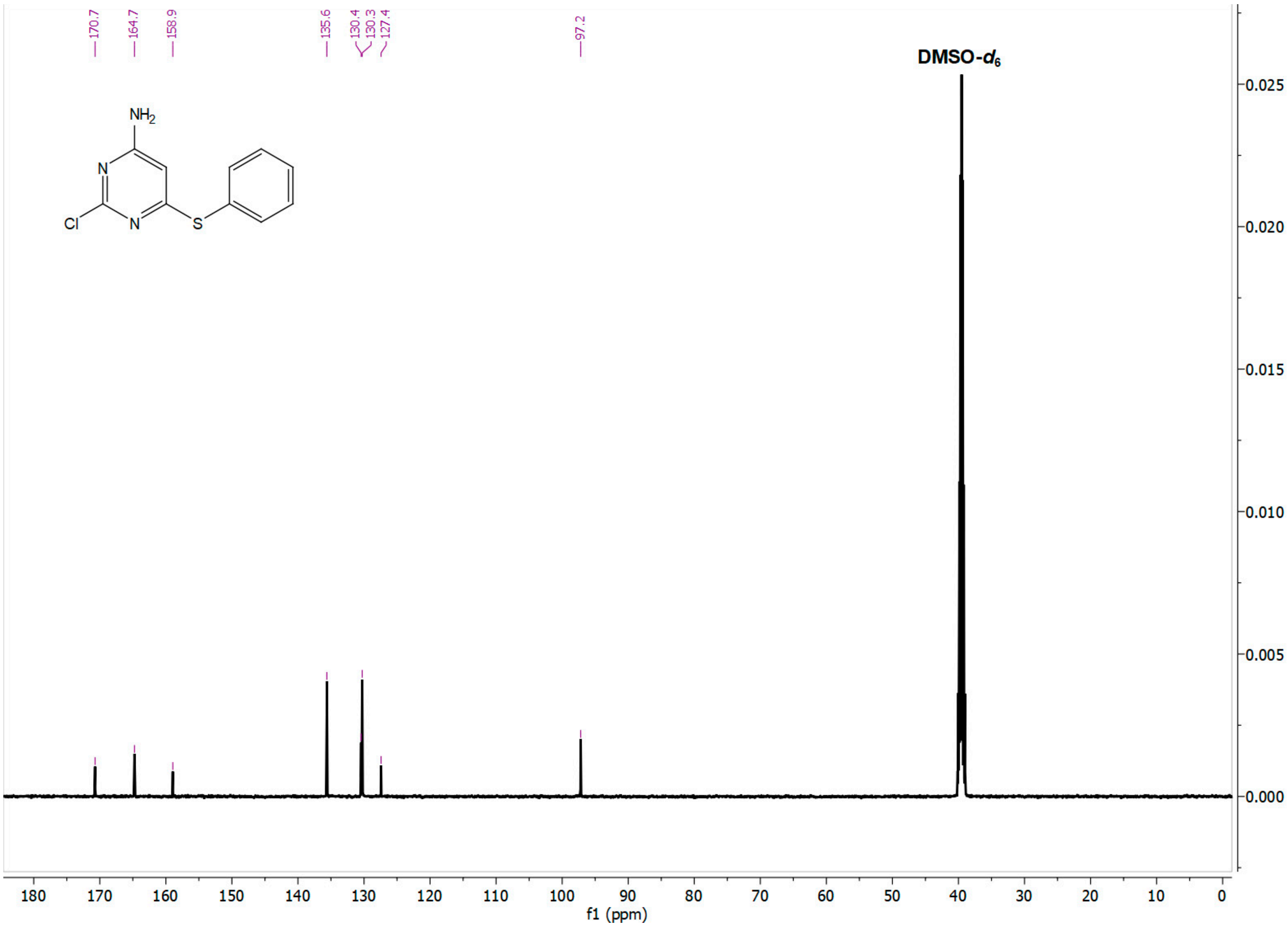

Figure S30. $^{13}C\{^1H\}$ NMR spectrum (126 MHz) of compound **C13** in DMSO-d_6.

S2. HRMS spectra of the synthesised compounds

Electrospray ionization mass spectrum in positive-ion mode (HRMS-ESI$^+$) of compound **1**

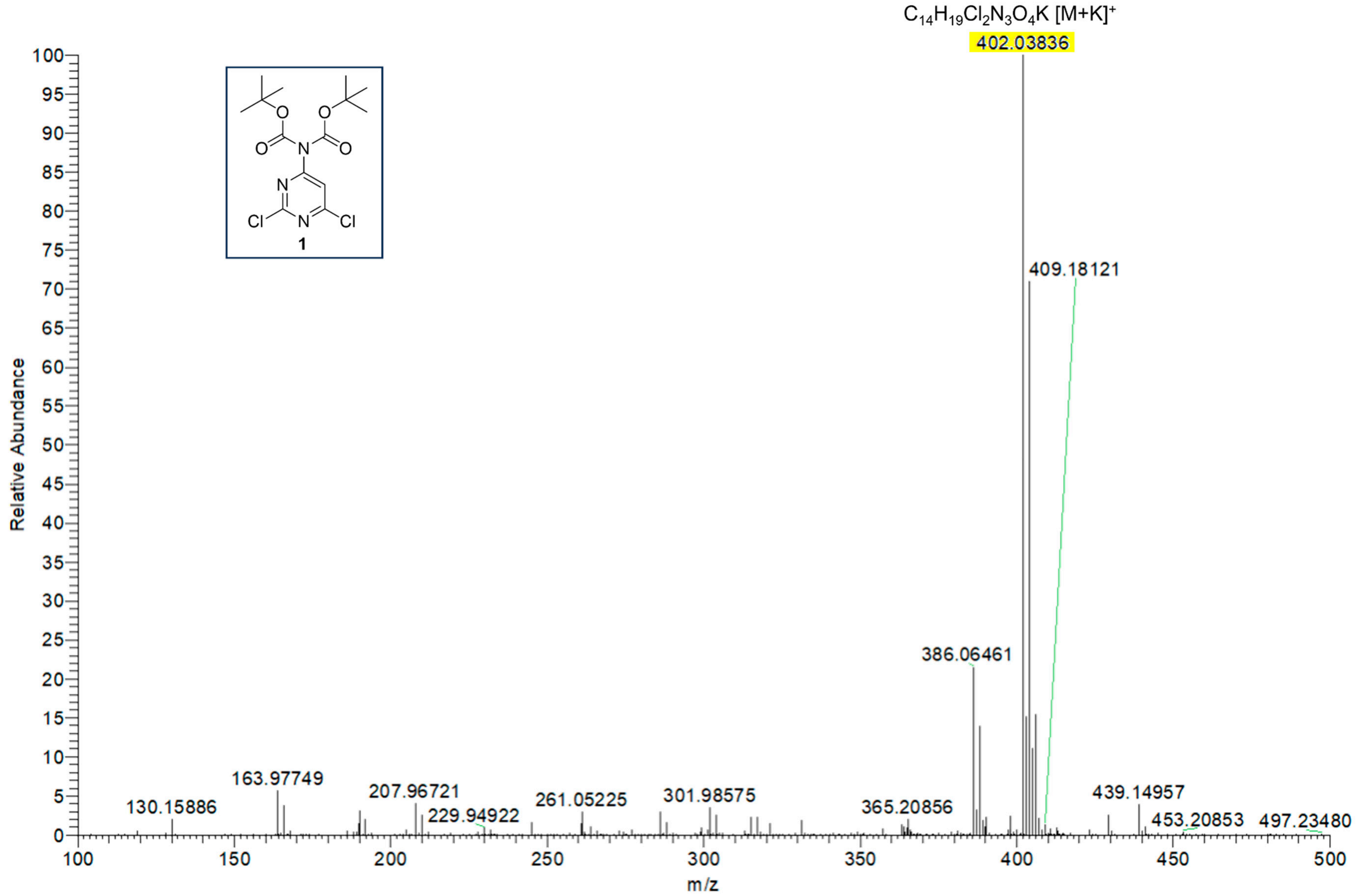

Electrospray ionization mass spectrum in positive-ion mode (HRMS-ESI$^+$) of compound **2**

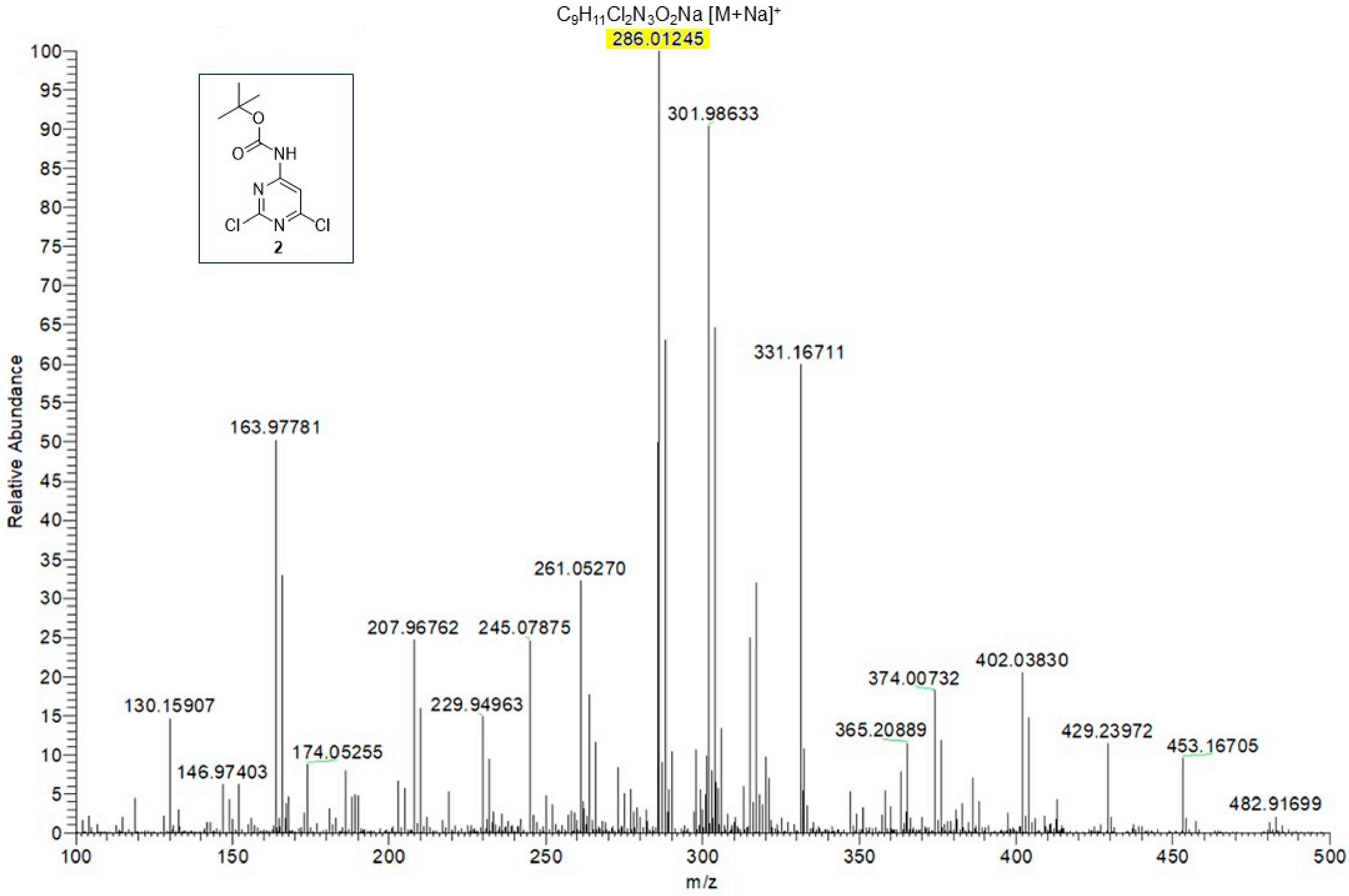

Electrospray ionization mass spectrum in positive-ion mode (HRMS-ESI$^+$) of compound **3**

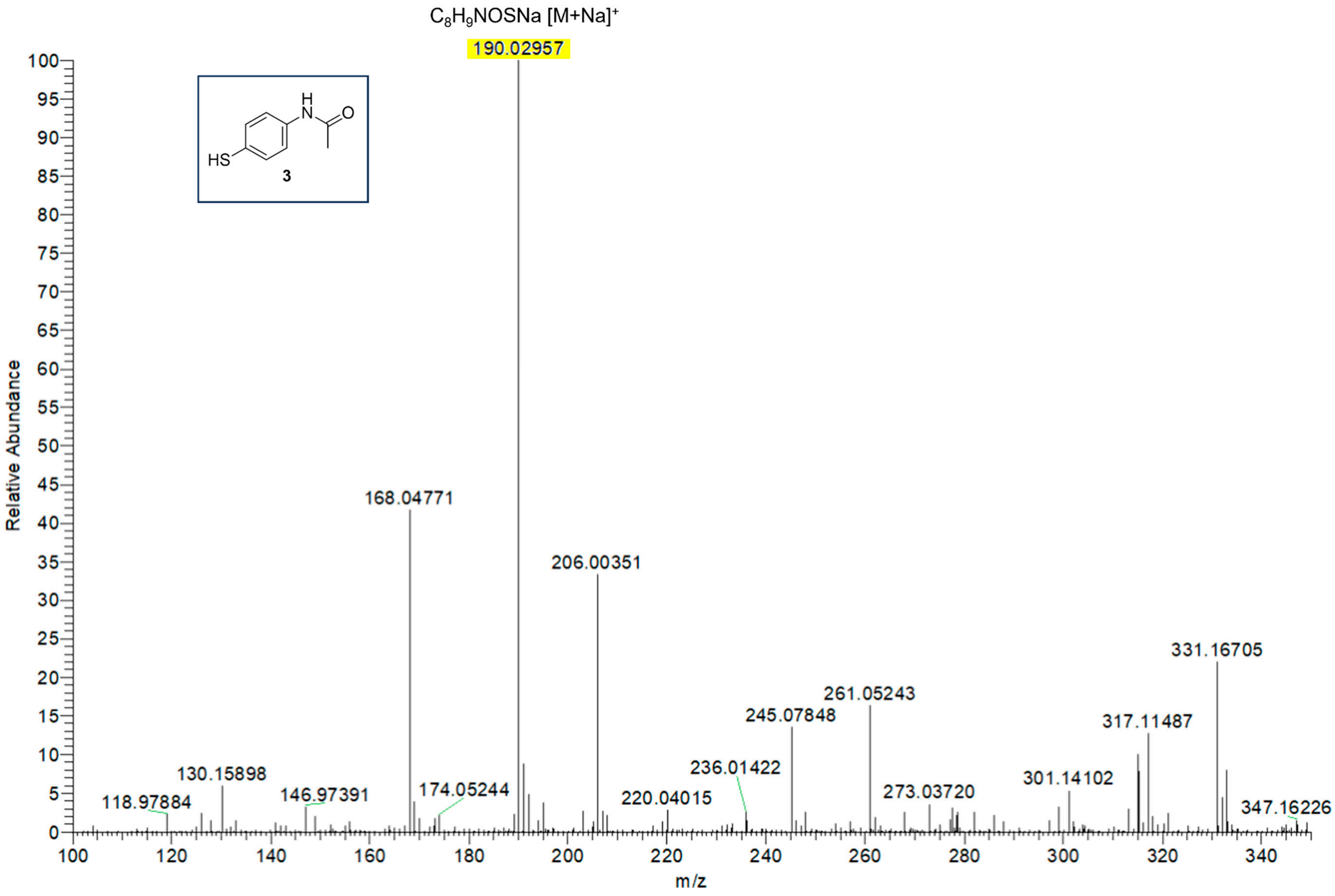

Electrospray ionization mass spectrum in positive-ion mode (HRMS-ESI$^+$) of compound **C1**

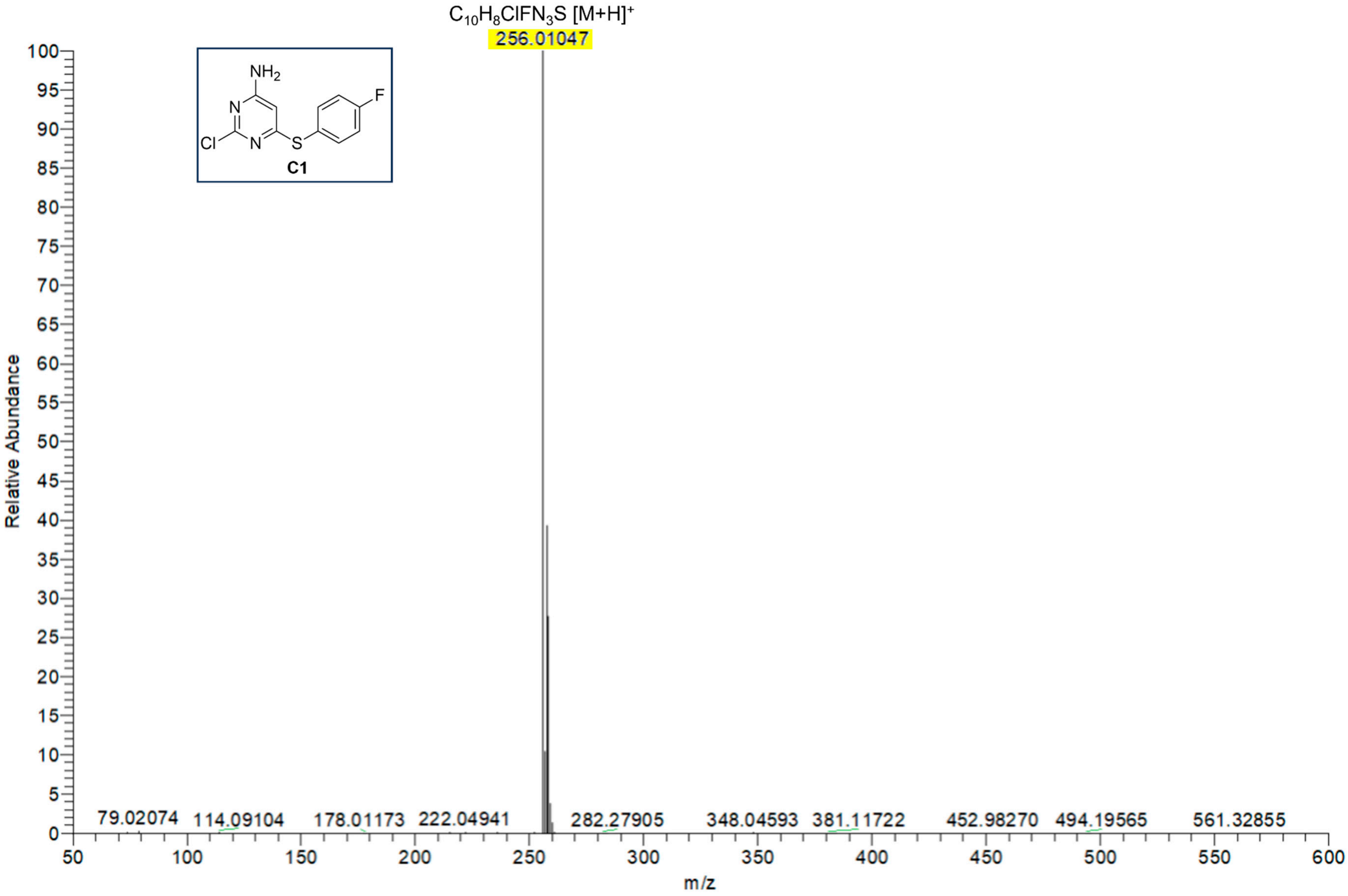

Electrospray ionization mass spectrum in positive-ion mode (HRMS-ESI$^+$) of compound **C2**

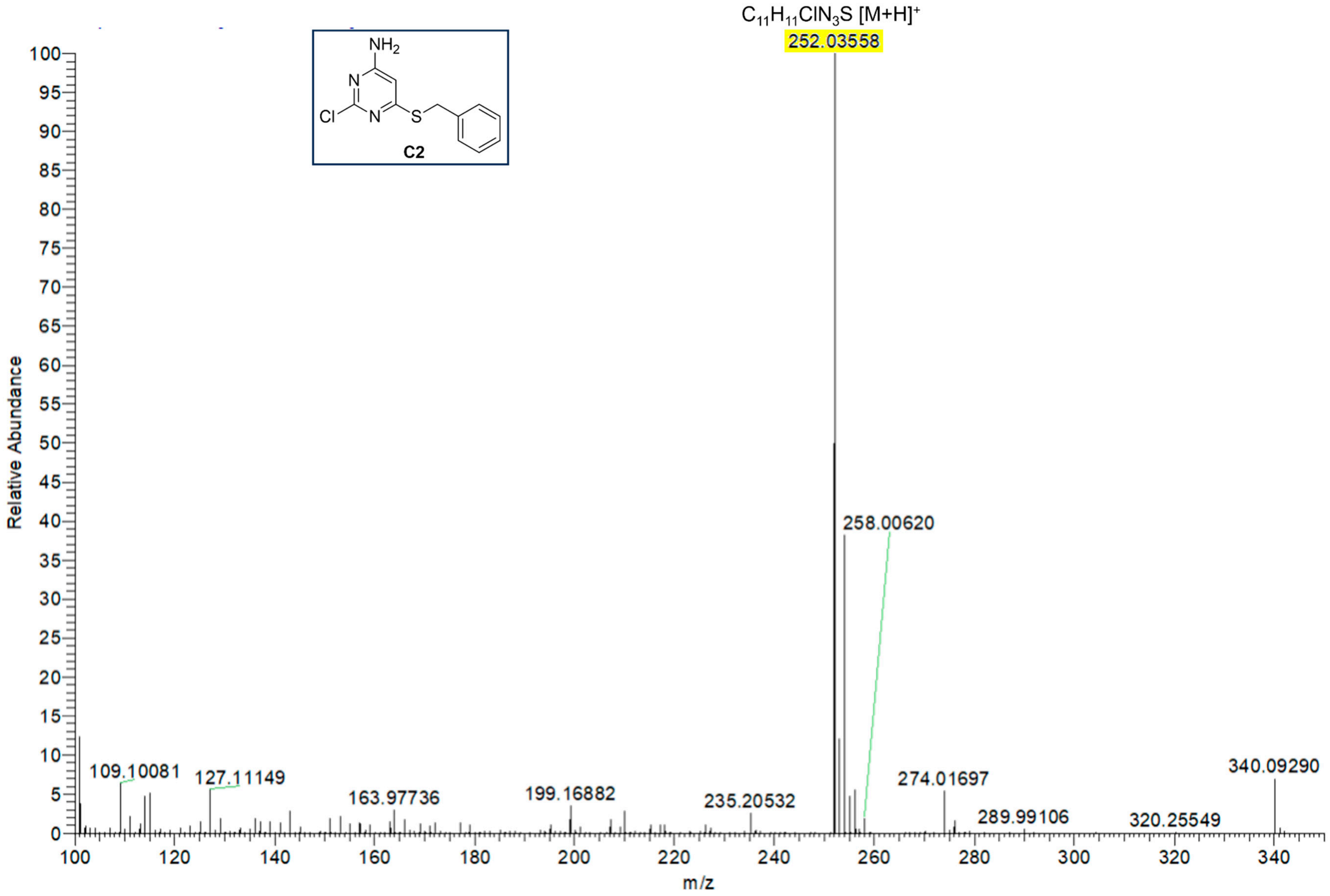

Electrospray ionization mass spectrum in positive-ion mode (HRMS-ESI$^+$) of compound **C3**

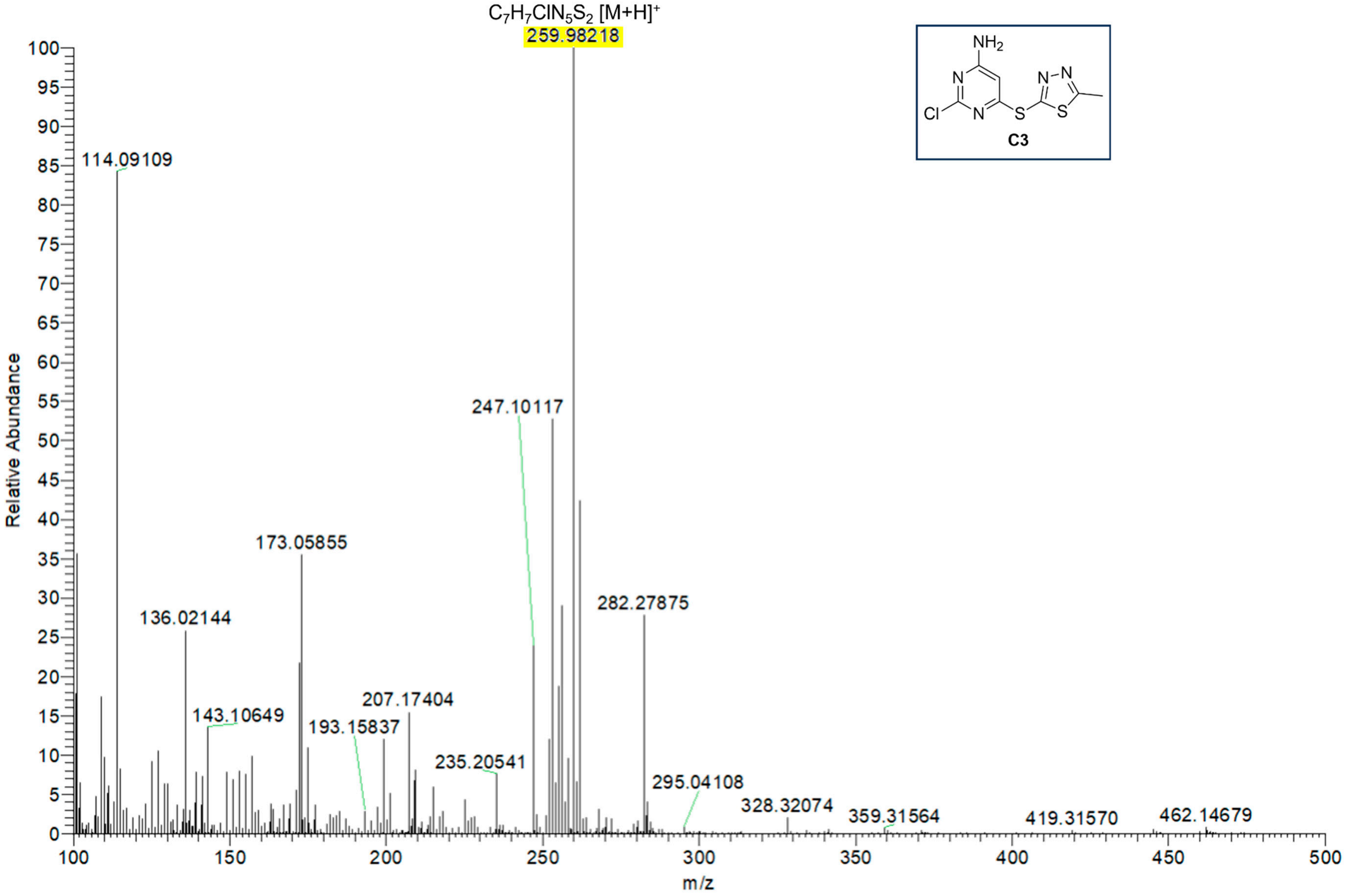

Electrospray ionization mass spectrum in positive-ion mode (HRMS-ESI+) of compound **C4**

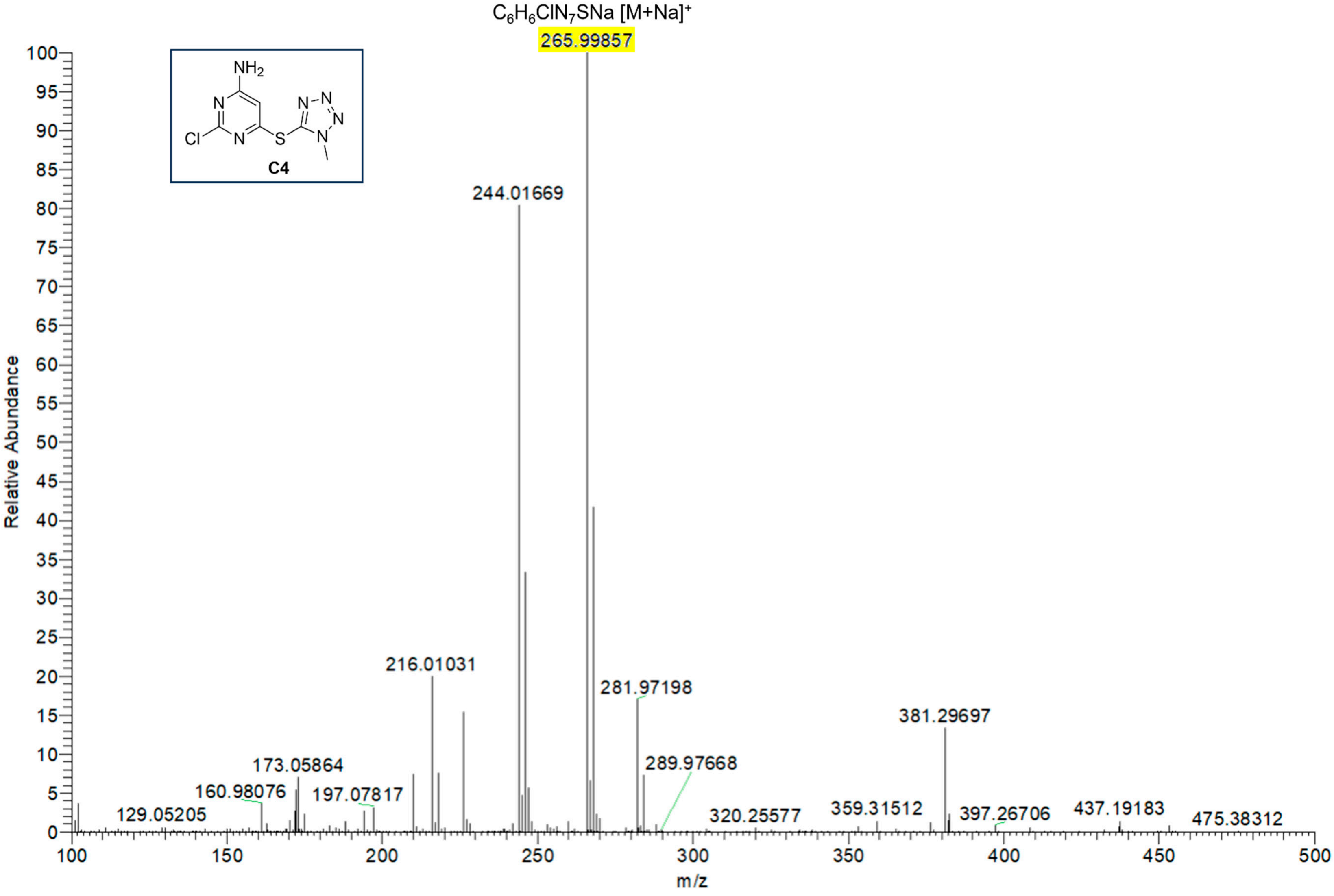

Electrospray ionization mass spectrum in positive-ion mode (HRMS-ESI$^+$) of compound **C5**

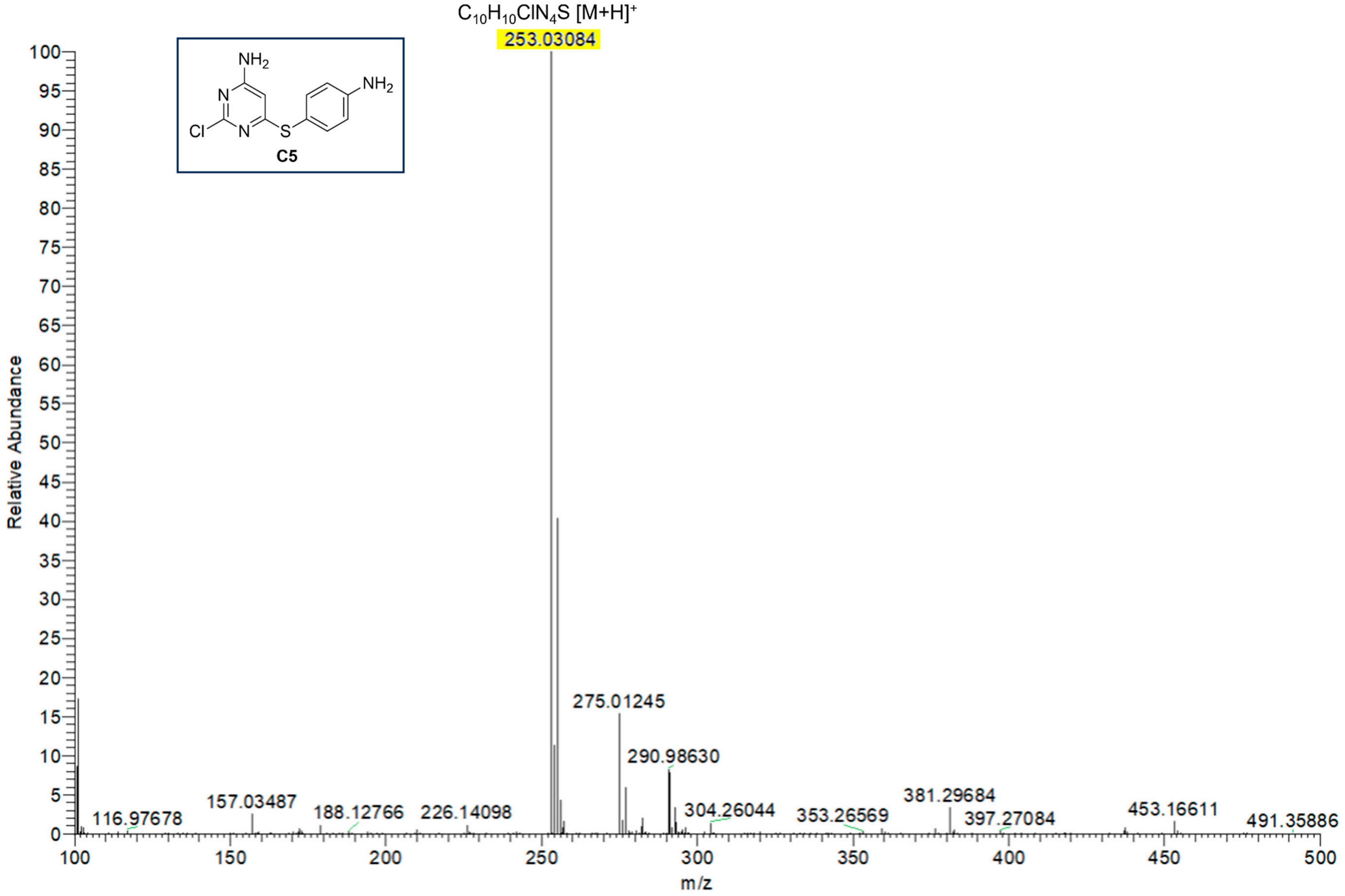

Electrospray ionization mass spectrum in positive-ion mode (HRMS-ESI$^+$) of compound **C6**

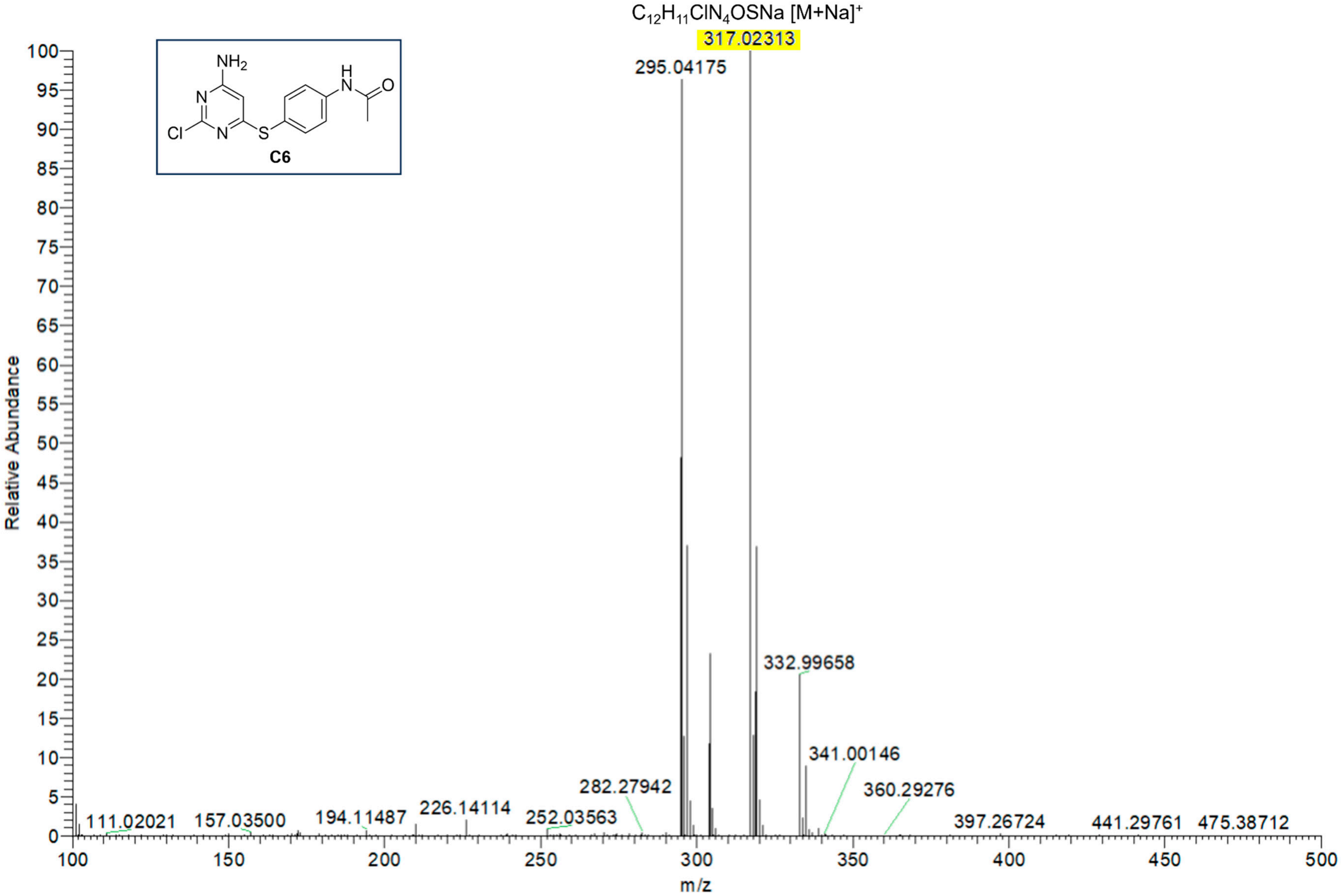

Electrospray ionization mass spectrum in positive-ion mode (HRMS-ESI$^+$) of compound **C7**

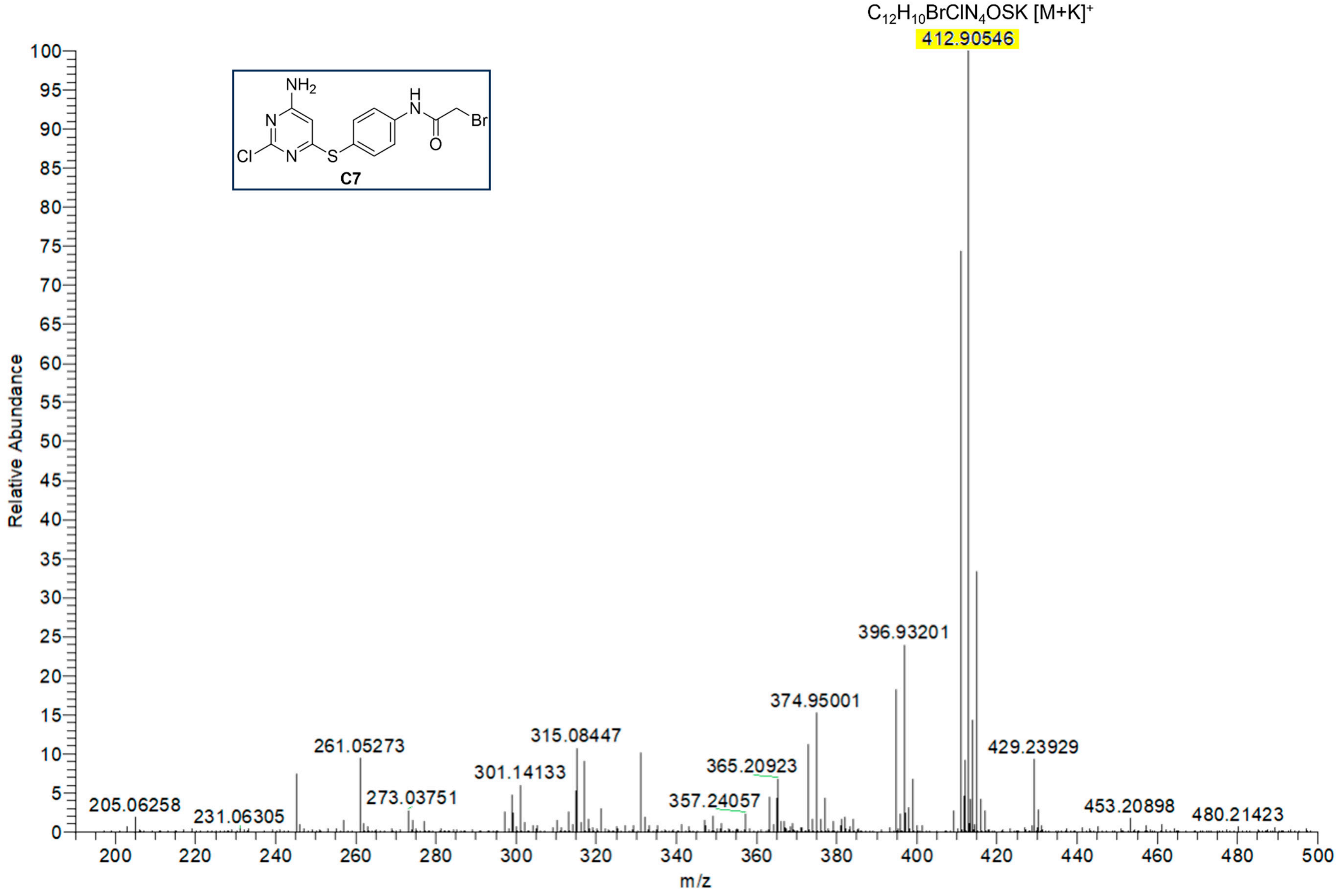

Electrospray ionization mass spectrum in positive-ion mode (HRMS-ESI$^+$) of compound **C8**

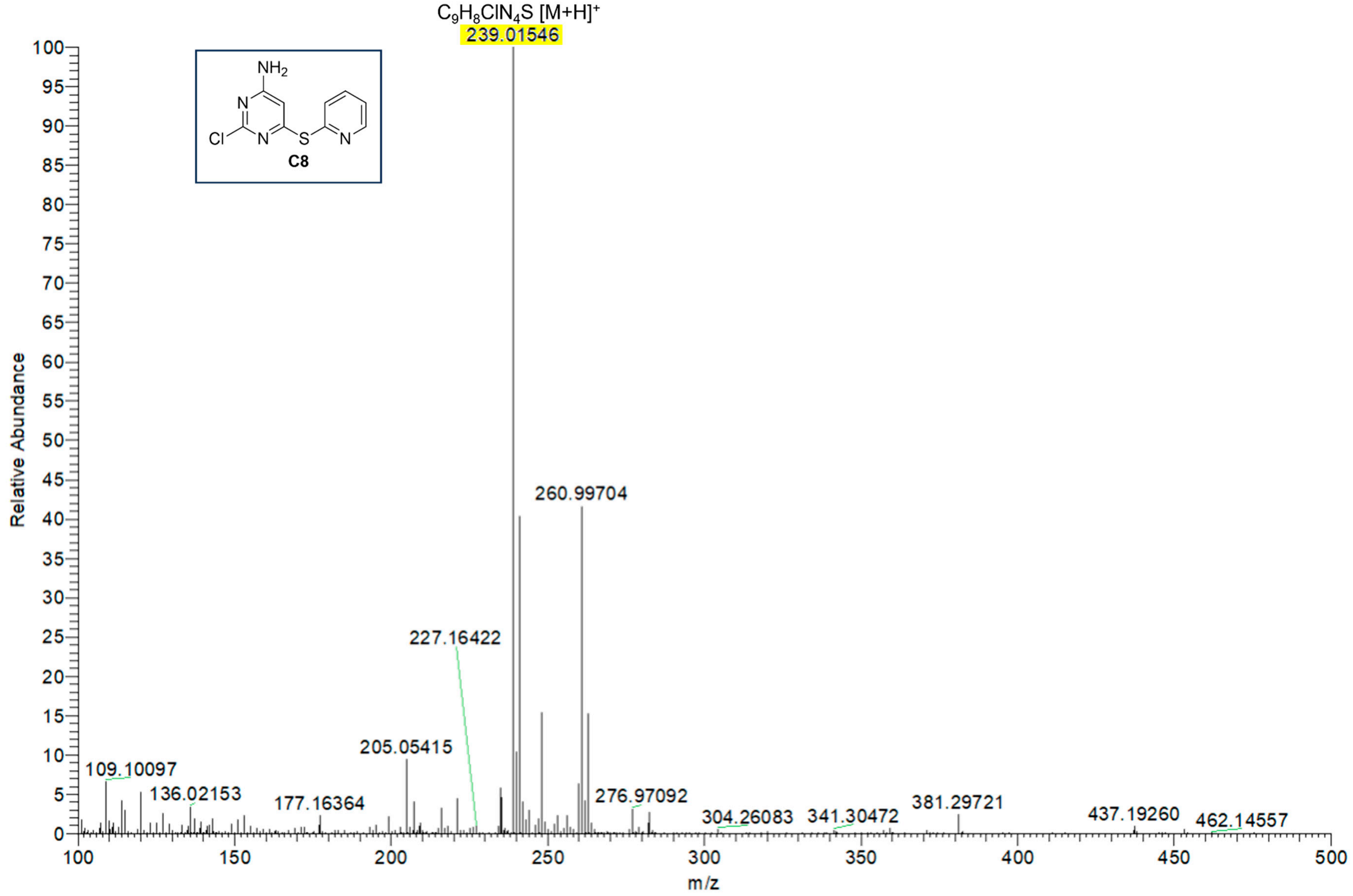

Electrospray ionization mass spectrum in positive-ion mode (HRMS-ESI+) of compound **C9**

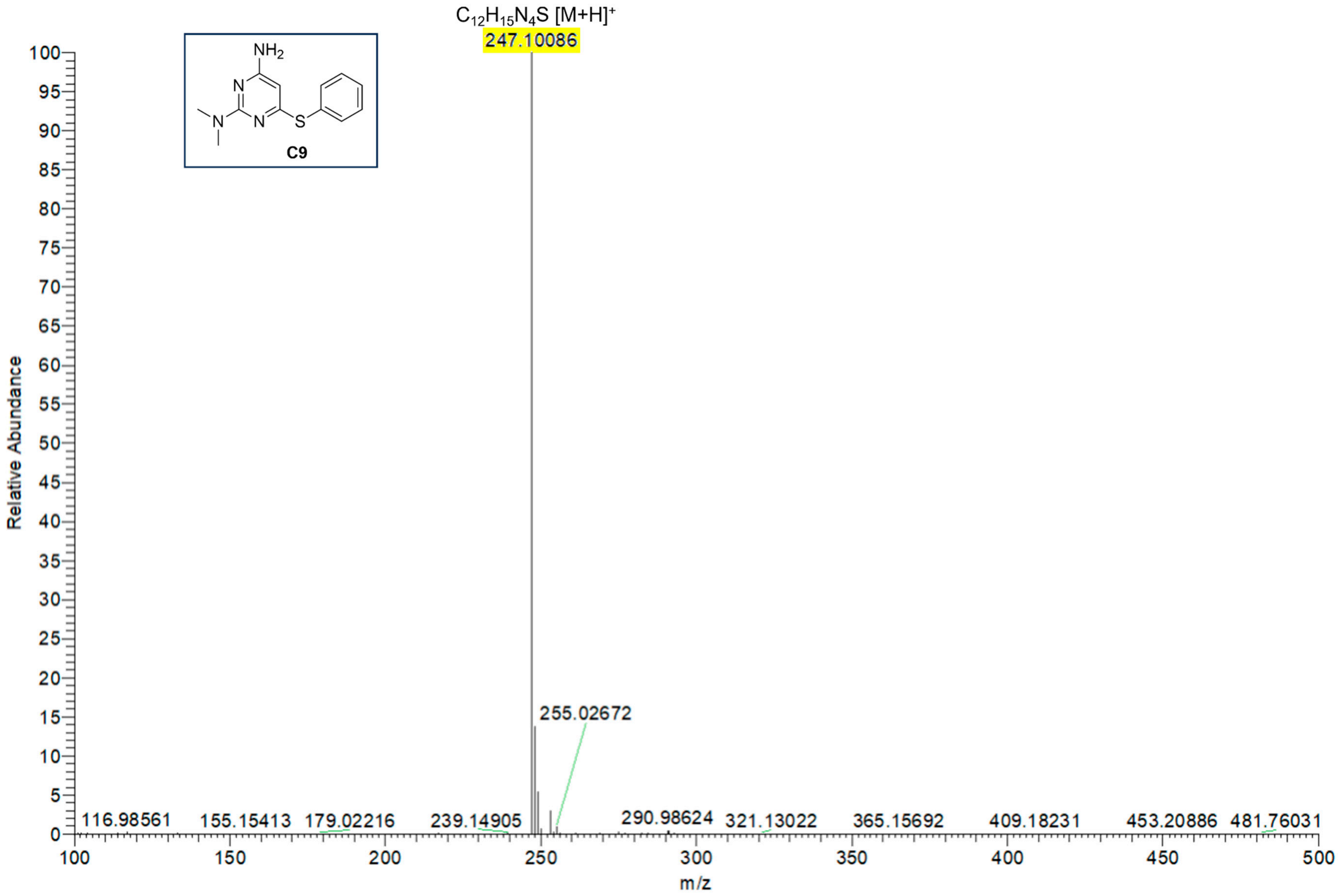

Electrospray ionization mass spectrum in positive-ion mode (HRMS-ESI$^+$) of compound **C10**

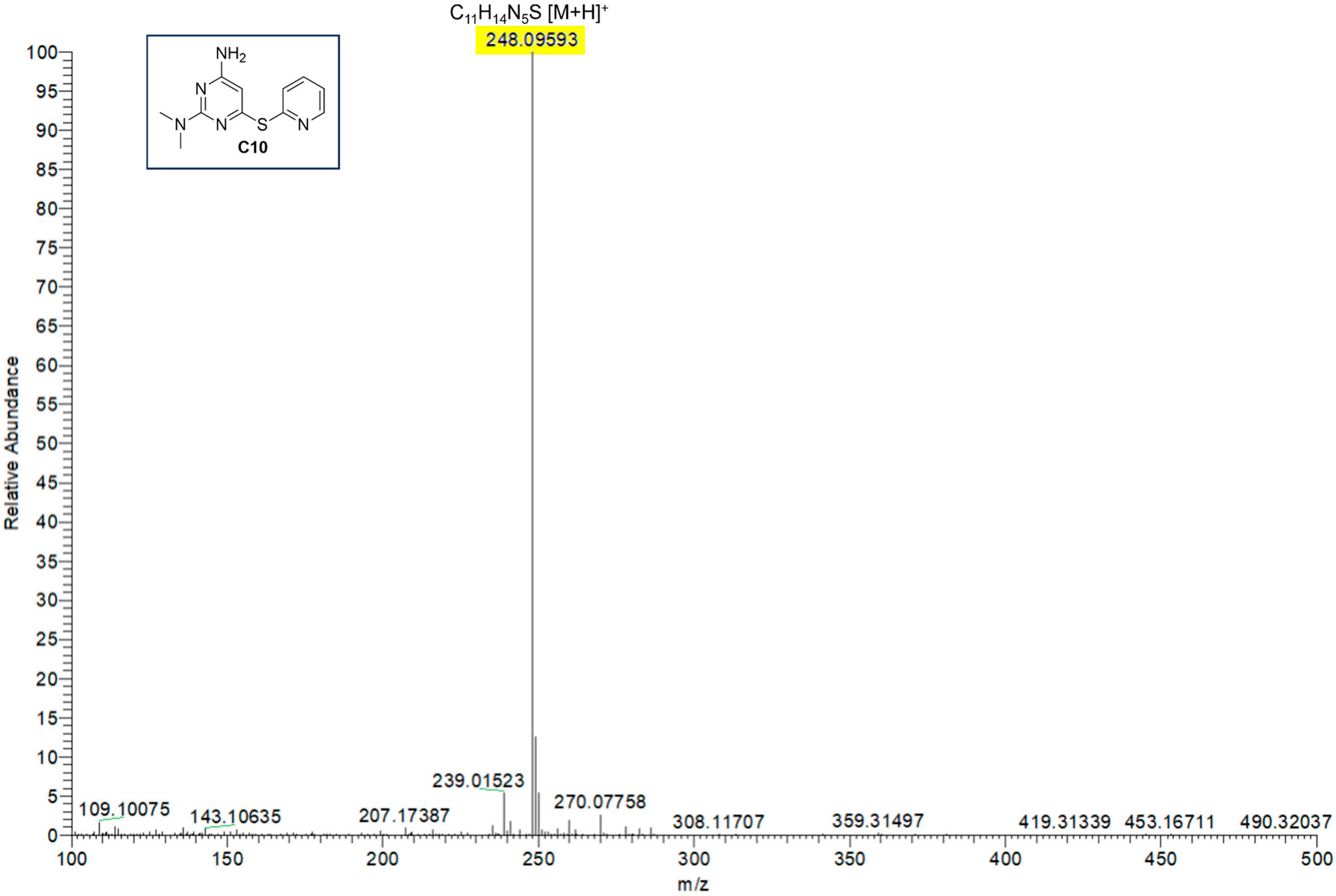

Electrospray ionization mass spectrum in positive-ion mode (HRMS-ESI$^+$) of compound **C12**

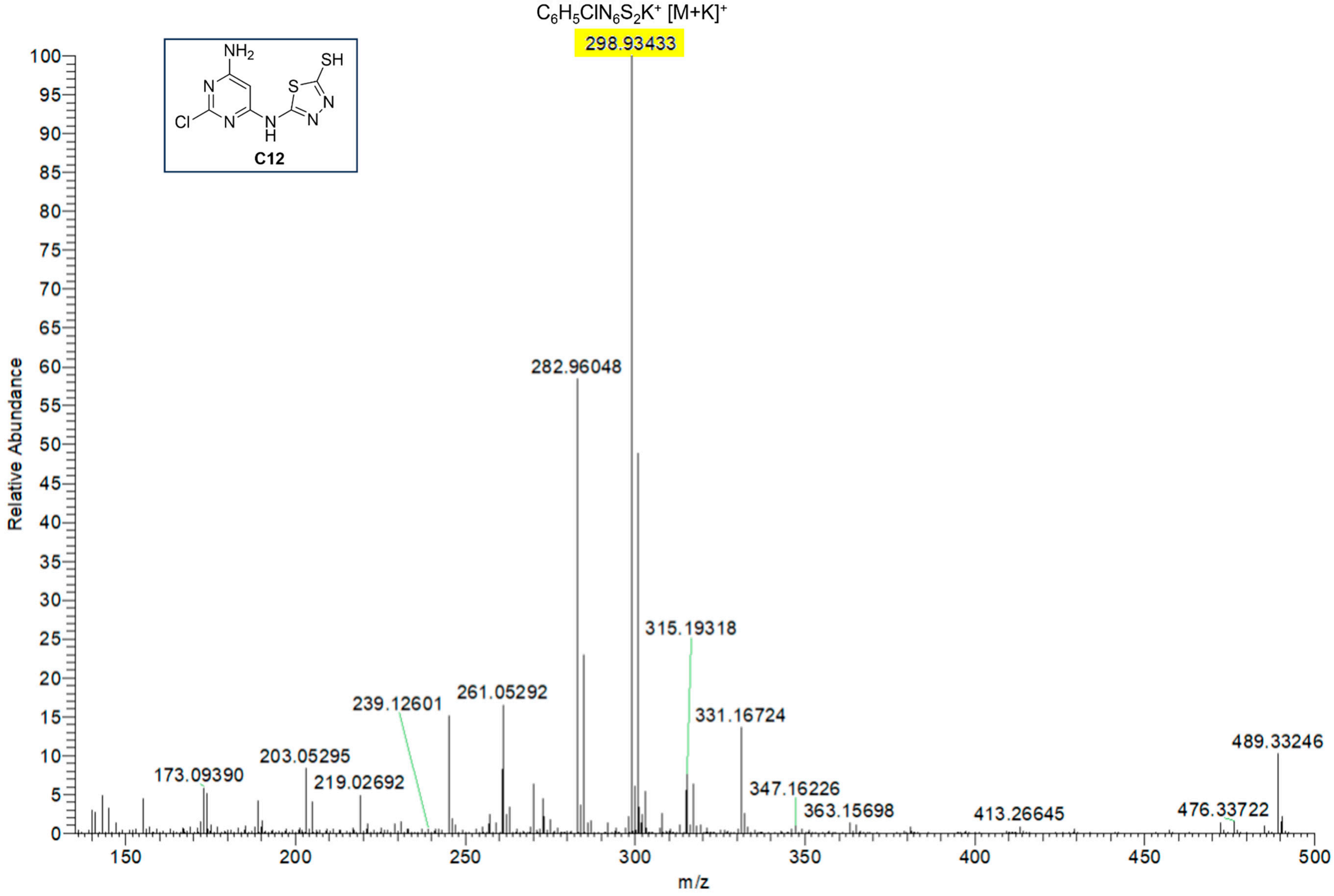

Electrospray ionization mass spectrum in positive-ion mode (HRMS-ESI$^+$) of compound **C13**

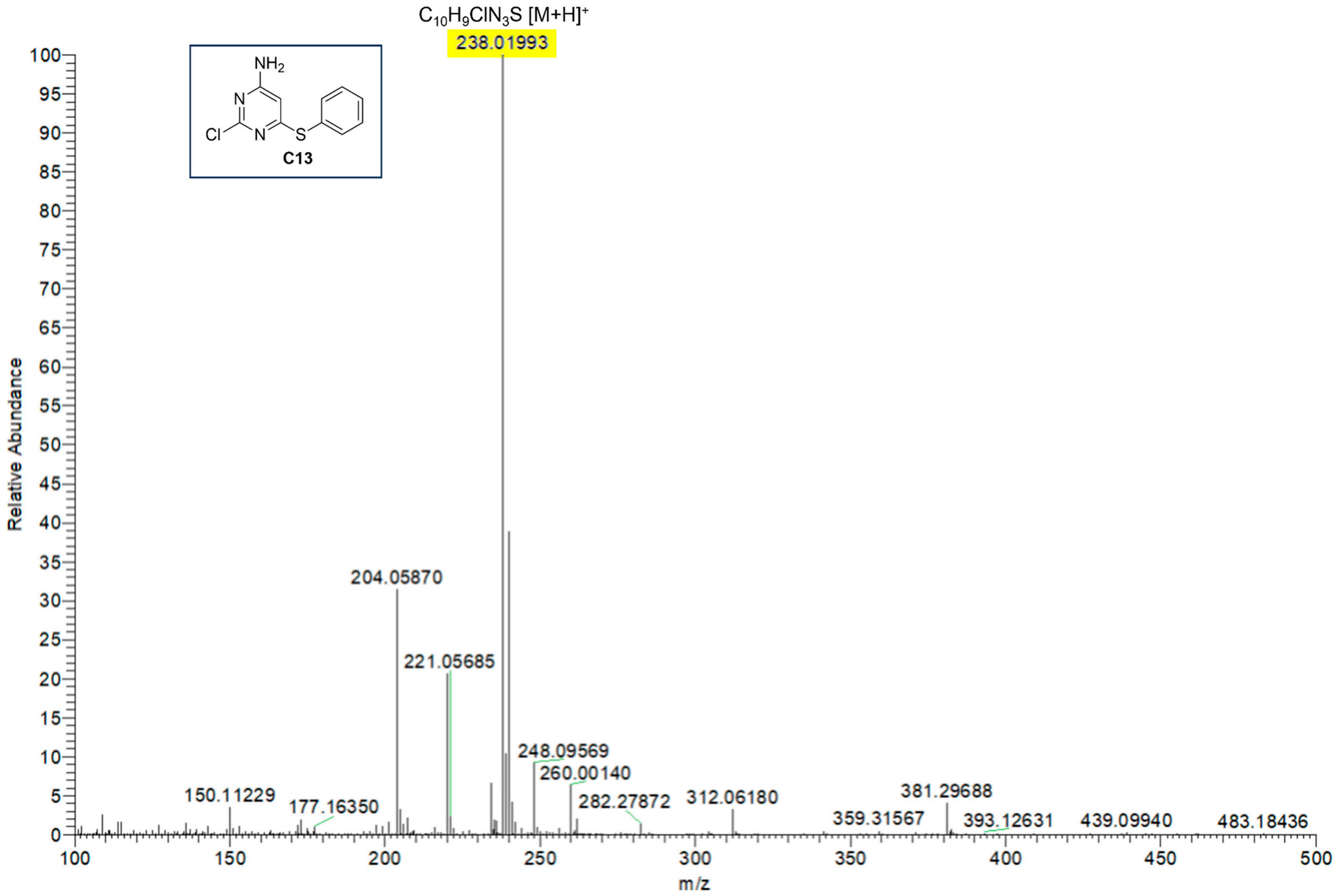

Supplement: Supplementary file 1 [file antioxidants-15-00841-s001.zip › antioxidants-4359458-supplementary.pdf]
